# Gender-equitable caregiver attitudes and education and safety of adolescent girls in South Kivu, DRC: A secondary analysis from a randomized controlled trial

**DOI:** 10.1371/journal.pmed.1003619

**Published:** 2021-09-28

**Authors:** Ilana Seff, Kathryn Falb, Gary Yu, Debbie Landis, Lindsay Stark

**Affiliations:** 1 Brown School at Washington University in St. Louis, Missouri, United States of America; 2 International Rescue Committee, Washington, District of Columbia, United States of America; 3 New York University, New York, New York, United States of America; 4 CARE International, Washington, District of Columbia, United States of America; The Hospital for Sick Children, CANADA

## Abstract

**Background:**

Adolescent girls face myriad threats to their well-being and safety as a result of gender-inequitable attitudes and norms, and these risks are often exacerbated during humanitarian emergencies. While humanitarian actors have begun to address caregivers’ behaviors and gender attitudes as an approach to support and meet the needs of adolescent girls, best practices for working with caregivers to improve adolescent girls’ well-being in these settings have yet to be identified.

**Methods and findings:**

This study uses panel data from a program evaluation to analyze associations between changes in gender-equitable attitudes among caregivers and changes in schooling and violence victimization for girls ages 10 to 14 years old in the Democratic Republic of the Congo (DRC). Participants were recruited in May 2015 for baseline (May to July 2015) and endline (August to October 2016) data collection. Baseline and endline data for both caregivers and girls were available for 732 girls. The average ages of adolescents and caregivers were 12 and 40.7, respectively, and 92% of caregivers were female. The predictor of interest was the change in caregivers’ gender-equitable attitudes between the 2 points in time, where attitudes were measured using 10 underlying survey questions. The primary outcomes of interest were dichotomous and included improvement in schooling participation and declines in physical, sexual, and emotional violence and feeling uncared for. Logistic regression was used to estimate the association between changes in caregivers’ attitudes and 5 outcomes of interest and revealed that an increase in a caregiver’s gender-equitable attitude score was associated with significantly greater odds of a girl experiencing an improvement in schooling participation (aOR = 1.08, CI [1.005, 1.154], *p* = 0.036) and of a girl experiencing a marginal decline in physical violence victimization (aOR = 1.07, CI [0.989, 1.158], *p* = 0.092). Analyses also revealed that older girls had lower odds of experiencing an improvement in schooling participation (aOR = 0.77, CI [0.686, 0.861], *p* < 0.001), physical violence (aOR = 0.86, CI [0.757, 0.984], *p* = 0.028), sexual violence (aOR = 0.86, CI [0.743, 1.003], *p* = 0.055), or emotional violence (aOR = 0.98, CI [0.849, 1.105], *p* = 0.005). Important limitations in this study include the self-reported nature of outcomes, use of single questionnaire items to construct the outcome variables, and potential self-selection bias.

**Conclusions:**

Results suggest that supporting caregivers to increase gender equitable attitudes may be associated with benefits in dual outcomes of education and safety for adolescent girls in eastern DRC. Further research is needed to better understand how to induce a shift in these attitudes in multisectoral programming.

**Trial registration:**

NCT02384642.

## Abstract 2

In an additional analysis of COMPASS intervention trial findings, Ilana Seff and colleagues study the associations between changes in gender attitudes among caregivers and violence victimization and schooling of adolescent girls in South Kivu, Democratic Republic of Congo.

## Author summary

**Why was this study done?:**

- Adolescent girls in humanitarian emergencies often face numerous risks to their safety and well-being, and these risks can be exacerbated by gender-inequitable attitudes and norms.
- Caregivers play important roles in the lives of adolescent girls, particularly with regard to girls’ access to education, safety, and the care they receive as dependent minors.
- This study aimed to understand if changes in gender attitudes among caregivers were associated with changes in violence victimization and schooling of their adolescent girls in South Kivu, Democratic Republic of Congo (DRC).

**What did the researchers do and find?:**

- The authors used panel data from an evaluation of the COMPASS program in the DRC to understand more about the relationship between changes in gender attitudes among caregivers and improvement in participation in schooling and violence victimization for adolescent girls.
- By analyzing data from 732 adolescent girls and caregivers at 2 points in time, the authors found that increases in gender-equitable attitudes of caregivers corresponded to statistically significant improvement in schooling participation and marginal declines in physical violence for adolescent girls.
- Importantly, the authors found that while adolescent girls of all ages experienced less violence and increased schooling participation as their caregivers built more gender-equitable attitudes, these benefits diminished for older girls.

**What do these findings mean?:**

- Based on their findings, the authors suggest that supporting caregivers to increase gender-equitable attitudes can be an important mechanism for improving adolescent girls’ participation in schooling and decreasing their rates of violence victimization.
- Findings that point to diminished benefits of increasingly gender-equitable attitudes of caregivers on the safety and school participation of older adolescent girls illustrate the importance of addressing gender attitudes when children and adolescents are as young as possible in order to reap the maximum benefit from these interventions.
- The authors note a few limitations to their study, the self-reported nature of outcomes, the use of single questionnaire items to construct the outcome variables, and potential self-selection bias.

## Introduction

Girls in adolescence, typically defined as the period from 10 to 19 years, face a multitude of threats to their well-being and safety as a result of gender-inequitable attitudes and norms. These vulnerabilities can range from limited educational and economic opportunities to exposure to child abuse, transactional sex, intimate partner violence (IPV), and child marriage [1–4]. For example, violence affects 1 in 4 girls under the age of 18 globally and is the second leading cause of death for adolescent girls [5–7]. Violence victimization during adolescence has been shown to cause numerous adverse health consequences both during adolescence and throughout adulthood, including sexually transmitted infections (STIs), unintended pregnancy, alcohol and substance abuse, depression, posttraumatic stress disorder, and suicide [8–15]. Additionally, approximately 29% of women ages 20 to 49 worldwide were married before age 18, and married girls are as much as 10 times more likely to drop out of school than their unmarried counterparts [16]. Moreover, adolescence itself is a particularly critical stage of development and susceptibility to social influence; girls exposed to gender-inequitable ideologies or consequences during this period are more likely to face similar experiences into adulthood [17,18].

Preexisting gender-inequitable beliefs and norms are often intensified during humanitarian emergencies, further exacerbating gender-inequitable vulnerabilities girls face during adolescence in these contexts [19–21]. Adolescent girls in humanitarian crises face an increased risk of exposure to violence, transactional sex, and forced marriage, while simultaneously being forced to cope with more limited supportive mechanisms [3,20,22–24]. For example, a multicountry study found that more than 60% of conflict-affected adolescent girls reported experiencing physical, sexual, or emotional violence [25]. Girls in crisis-affected environments may also face disruptions in schooling due to community instability as a result of conflict [4,26]. Further, conflict and displacement create familial financial constraints, and, in contexts where education is more valued for men, these constraints increase the likelihood that caregivers will prioritize schooling for their sons over their daughters [27]. In fact, girls in conflict-affected areas are more than 2.5 times more likely than boys to be out of school [28]. Inequitable gender expectations may also directly or indirectly pressure girls to drop out of school to help with homemaking or prioritize marriage or pregnancy [27,29].

Evidence from humanitarian and nonhumanitarian settings alike points to the influential role that the family sphere, and caregivers and parents in particular, can play in promoting adolescent girls’ well-being [30,31]. Cross-sectional data suggest that gendered attitudes of caregivers may determine when and how they provide their daughters with emotional support, influence the rules they enact around girls’ mobility, and shape girls’ own attitudes and norms [32]. Caregiver gender-inequitable attitudes also serve as a risk factor for adolescent girls’ exposure to violence, as previous cross-sectional research has found that caregiver acceptance of wife-beating and corporal punishment is associated with an increased risk of abuse of children [33]. Further, evidence from humanitarian settings suggests that mothers who promote accepting attitudes toward IPV are more likely to encourage their daughters to stay in abusive relationships [34]. A wide range of cross-sectional evidence also confirms the associations between caregivers’ attitudes and perceptions on girls’ safety and educational mobility. For example, research has shown that, when faced with resource constraints, caregiver perceptions of the economic return of investing in sons versus daughters may lead to inequitable gender differences in intrahousehold investments [35–37]. Educational outcomes associated with these differential investments may include later school enrollment, fewer years of schooling, and lower quality of education for girls as compared to boys [38–40].

In the same way that norms and attitudes that endorse gender inequity and violence against women and girls can be harmful to girls, more equitable gender norms can be transformative, promoting girls’ greater involvement in education and consequently improving overall development. Gender-equitable attitudes may increase caregivers’ likelihood of offering intentional support to their adolescent girls, which can, in turn, prevent girls’ risky behaviors and improve positive mental health and coping strategies [41]. Seminal research on the topic of gender-equitable households and risk of violence in low- and middle-income countries indicates that supporting gender-equitable attitudes among caregivers may also have spillover effects on reducing men’s perpetration of physical IPV [42–44]; one study conducted in Bangladesh found that men’s childhood exposure to gender-equitable parental decision-making was protective against physical IPV perpetration in adulthood [45]. The reduction of boys’ future perpetration of physical IPV may be an added benefit of gender-equitable interventions, above and beyond improvements in girls’ more immediate schooling and exposure to violence.

In recognition of the relationships between caregiver gender attitudes and violence victimization and schooling of adolescent girls, a burgeoning strategy within humanitarian settings has been to engage caregivers to better support and meet the needs of their adolescent girls by addressing both parenting behaviors as well as gender attitudes and norms among caregivers [12,20]. However, while a small but growing number of approaches are being implemented and evaluated [46,47], promising practices for working with caregivers to improve adolescent girls’ well-being in humanitarian settings have yet to be identified; practitioners working to protect adolescent girls in these contexts have called for an increased understanding of how caregivers can contribute to girls’ well-being and safety [48–50]. A prior study using the cross-sectional baseline data alone found that more equitable gender attitudes on the part of girls and caregivers were associated with girls’ participation in formal education [51]. While this and other cross-sectional evidence as outlined above points to the associations between caregivers’ gender equitable attitudes and girls’ well-being, little is known about how changes in attitudes might lead to changes in experiences for girls, particularly in humanitarian settings. For example, in settings with significant resource constraints, a number of factors may contribute to girls being out of school; parents who rely on girls to complete household tasks may not suddenly have the ability to prioritize their daughters’ schooling after developing more gender-equitable attitudes [20]. Developing caregiver programs that ultimately are able to improve girls’ lived experiences requires a more nuanced understanding of mutable caregiver characteristics and family dynamics.

The present study adds to existing literature about the associations between caregivers’ gender-equitable attitudes and improved outcomes of well-being for girls. While the cross-sectional nature of these associations is better documented, less is known about whether changes in caregivers’ attitudes can engender corresponding changes in experiences for girls. This study seeks to fill these gaps in knowledge by analyzing the associations between improved gender equitable attitudes among caregivers and changes in schooling and violence victimization for girls in a conflict-affected setting in the Democratic Republic of the Congo (DRC). Furthermore, the literature on these family relationships in humanitarian settings specifically are still developing; the findings presented here add to this growing evidence base.

## Methods

### Study setting and design

Data analyzed for this study were collected as part of a mixed-methods, cluster-randomized controlled trial (registration #NCT02384642) evaluating the Creating Opportunities through Mentorship, Parental Involvement, and Safe Spaces (COMPASS) program. The evaluation sought to examine the incremental impact of adding a caregiver curriculum to a 12-month girls’ life skills and empowerment program. Adult and adolescent participants were recruited in May 2015, with the initial data collection phase taking place from May to July 2015 and endline data being collected from August to October 2016. COMPASS was implemented in 14 village sites across conflict-affected South Kivu, the current home to nearly 15% of DRC’s internally displaced population [49,52]. The risks of violence for adolescent girls in protracted conflicts in general, and in the Kivus specifically, are well documented; approximately 50% of girls ages 10 to 14 years in this setting report having experienced at least some form of violence in the last 12 months [53].

Study participants comprised caregivers and girls ages 10 to 14 years who spoke Swahili, French, or Mashi. Separate survey questionnaires were employed for girls and caregivers. The girls’ questionnaire collected information on a range of topics, including, but not limited to, physical, emotional, and sexual violence exposure, schooling, life aspirations, and other health- and sexual-related behaviors; the caregiver questionnaire captured information on harsh discipline practices, parental acceptance and rejection, and gender-inequitable attitudes. Baseline questionnaires were administered from May to July 2015, and the surveys were readministered to the same sample at endline from August to October 2016. Both the girls’ and caregivers’ surveys were available in Swahili, French, or Mashi, and trained interviewers comprised native speakers of all 3 languages. The caregiver questionnaire and the majority of the girl questionnaire were administered by an interviewer using computer-assisted personal interviewing (CAPI). More sensitive questions for girls, such as those on sexual behaviors and exposure to violence, were self-administered using audio computer-assisted self-interview (ACASI) software [54]. Further, some of the sensitive questions around marriage and sexual violence were not administered to 10- to 12-year-olds due to ethical and cultural concerns. All data were collected in private rooms provided by local community-based organizations.

Informed consent was obtained for all married participants and those aged 18 and older; parental consent and adolescent assent were collected for all unmarried girls under 18. Baseline and endline data for both caregivers and girls were available for 732 girls. Further information on the study design, procedures, participant selection, and follow-up, including sample size calculations, is detailed elsewhere [46,49].

This study is reported as per the Strengthening the Reporting of Observational Studies in Epidemiology (STROBE) guideline (S1 Checklist).

### Predictor and outcomes of interest

This study presents secondary analyses of the COMPASS data, for which the analytical plan was developed following completion of the trial. In order to examine how changes in caregiver attitudes were associated with changes in outcomes for girls, the present study leveraged the panel structure of the data. Given the post-conflict nature of the study setting, the a priori hypothesized trajectory was that girls would exhibit improvement in outcomes, if they exhibited any change at all. The predictor of interest was the change in caregivers’ gender-equitable attitudes between baseline and endline. Caregivers’ gender-equitable attitudes were measured at each point in time using 10 underlying survey questions [54]. Caregivers were asked to agree or disagree with 10 statements related to gender roles and dynamics, such as “it is more important that sons have education than daughters,” “if there is a limited amount of money to pay for schooling/tutoring, it should be spent on sons first,” and “a good woman never questions her husband’s opinions, even if she is not sure she agrees with them,” among others. A caregiver’s gender-equitable attitude score was created to equal the number of statements a caregiver disagreed with and could thus assume a value from 0 to 10. As such, higher scores reflect more gender-equitable attitudes. The Cronbach alpha for the scale at baseline and endline was 0.799 and 0.821, respectively, indicating acceptable internal consistency at both time points. Changes in gender-equitable attitudes were calculated by subtracting baseline scores from endline scores, where the change may range from −10 to 10. Girl’s age was also included in the analytical model as age in years.

The primary outcomes of interest comprised improvement in schooling participation and declines in physical, sexual, and emotional violence, and feeling uncared for. In the cross-section, school participation was operationalized as a categorical variable grouping girls into 1 of 4 categories: (1) those who reported never attending school; (2) those who attended school some point but missed the entire last year; (3) those who reported current enrollment in school but missed at least 1 day in the past week; and (4) those who reported currently attending school and did not miss any days in the last week [26]. For the regression analysis, “improvement in school participation” was defined in the binary; respondents received a value of “1” if they experienced an improvement in the categorical schooling variable and received a “0” otherwise.

Exposure to past-year physical violence was constructed from a single item and defined in the binary; girls who reported that someone had “hit or beat and hurt [their] body” in the past 12 months were categorized as having experienced physical violence. For 13- to 14-year-olds, past-year sexual violence was defined as a “1” if the respondent reported having been “touched in a sexual way without permission,” been in a situation where someone “tried to use their influence or authority to threaten or pressure [the respondent] into having sex,” or had “sex unwillingly” within the last 12 months; the outcome included coerced sex and unwanted sexual touching only for 10- to 12-year-olds, as younger girls were not asked about their exposure to forced sex. Past-year exposure to emotional violence was constructed from a single question item and captured whether anyone had “screamed or aggressively yelled” at the girl respondent in the past 12 months, and feeling uncared for was defined when a girl reported “feeling uncared for” by the person responsible for their caregiving. For analysis, an improvement in each of the 4 outcomes was defined in the binary, where respondents received a “1” if they reported experiencing the outcome in the past 12 months at baseline but not at endline and received a “0” otherwise.

### Analysis

Descriptive statistics for all variables of interest were first summarized for baseline and endline, and differences between the 2 time points for the full sample were assessed using chi-squared and paired *t* tests for dichotomous and continuous variables, respectively. Logistic regression was used to estimate the difference in the outcomes of interest referenced above. Independent variables included in each regression comprised the change in caregivers’ equitable attitudes between baseline and endline, girls’ age, caregivers’ age, caregivers’ gender, treatment status, and village fixed effects. Data were missing for approximately 10% of the sample at each time point. We implemented sensitivity analyses on imputed data in order to ensure that findings were robust to any potential bias deriving from missingness. A multiple imputation approach was utilized to address missing predictors and outcomes. Using “Mi impute” in Stata, a set of 10 imputations was generated for each missing value. All analyses were conducted using Stata14.

### Ethics approval

This study was approved by the Columbia University Medical Center Institutional Review Board (protocol number AAAP6855) and the Ministry of Gender in South Kivu, DRC. Written consent was obtained for all participants.

## Results

Table 1 presents baseline and endline characteristics for the sample. On average, girls and caregivers were 12 and 40.7 years old, respectively, at baseline, and approximately 92% of caregivers were female. Overall, girls’ school participation improved between baseline and endline; while 20.7% of the sample had never attended school at baseline, this figure decreased to 14.6% at endline. Improvements for girls who had been in school at some point, but who missed all of the previous year, or who had missed one or more days of school in the past week, were also observed between the 2 points of data collection. There was evidence of a decline in physical and sexual violence (41.9% to 25.8% (*p* = 0.014) and 24.3% to 18.9% (*p* = 0.014), respectively), and apparent declines in feeling uncared for and emotional violence (48.1% to 43.5% (*p* = 0.087) and 43.2% to 38.8% (*p* = 0.076), respectively) were also observed but did not reach statistical significance. Finally, caregivers’ gender-equitable attitudes increased from 3.35 to 4.05 between baseline and endline (*p* < 0.001).

**Table 1:** Variables of interest, baseline and endline (*n =* 732).

	Baseline	Missing (n)	Endline	Missing (n)	*p*-value of *t* test or chi-squared
Girls					
Age	12.03	0	---	---	---
	[1.50]				
School participation		0		0	<0.001
Never attended	160		113		
	(20.7%)		(14.6%)		
Ever but missed all of last year	270		185		
	(34.9%)		(23.9%)		
Currently attending, missed > = 1 day in last week	136		216		
	(17.6%)		(27.9%)		
Currently attending, missed 0 days in last week	208		260		
	(26.9%)		(33.6%)		
Physical violence	286	55	244	47	0.014
	(41.9%)		(35.8%)		
Sexual violence	155	89	121	68	0.014
	(24.3%)		(18.9%)		
Feeling uncared for	315	82	285	48	0.087
	(48.1%)		(43.5%)		
Emotional violence	284	69	255	60	0.076
	(43.2%)		(38.8%)		
Caregivers					
Age	40.70	0	---	---	---
	[42.49]				
Female	780	0	---		
	(92.0%)				
Gender-equitable attitudes	3.35	18	4.05	27	<0.001
	[2.61]		[2.68]		

Note: Statistics are mean [SD] or n (%). Samples for each outcome are limited to those with nonmissing observations at both baseline and endline. Differences between baseline and endline were assessed using *t* tests for continuous variables and chi-squared tests for categorical and dichotomous variables.

Table 2 shows improvements in the predictor and outcomes of interest at the individual level. A little over 35% of girls reported an improvement along the continuum of school participation; the remaining sample saw either no change or a decline in school participation. Additionally, approximately one-fifth to one-fourth of the sample reported experiencing either physical, sexual, emotional violence, or feeling uncared for at baseline but not at endline.

**Table 2:** Changes between baseline and endline.

Girls	n (%)
Improvement in schooling participation	272
	(35.1%)
Improvement in physical violence	166
	(24.3%)
Improvement in sexual violence	112
	(17.5%)
Improvement in feeling uncared for	169
	(25.8%)
Improvement in emotional violence	148
	(22.5%)
Caregivers	Mean [SD]
Change in gender-equitable attitudes	0.71
	[2.60]

Note: Statistics are mean [SD] or n (%). Improvement in schooling is defined as “1” if respondent’s categorical school participation increased between baseline and endline and is “0” otherwise. Improvements in types of violence and feeling uncared for are defined as “1” if respondent experienced outcome at baseline and not at endline and “0” otherwise.

Findings from the adjusted regression analyses are presented in Table 3 (see S1 Table for unadjusted results). Each one-point increase from baseline to endline in a caregiver’s gender-equitable attitude score was found to be associated with 1.08 greater odds of a girl experiencing an improvement in school participation (95% CI [1.005, 1.154]; *p* = 0.036) and marginally greater odds of a girl experiencing a decline in physical violence victimization (aOR = 1.07; 95% CI [0.989, 1.158]; *p* = 0.092). Changes in caregivers’ attitudes were not found to be associated with sexual (95% CI [0.927, 1.112]; *p* = 0.746) or emotional violence (95% CI [0.946, 1.114]; *p* = 0.524) or feeling uncared for (95% CI [0.946, 1.114]; *p* = 0.510).

**Table 3:** Estimating changes in girls’ outcomes, adjusted odds ratios.

	Improvement in:				
	School participation aOR [95% CI] *p*-value	Physical violence aOR [95% CI] *p*-value	Sexual violence aOR [95% CI] *p*-value	Feeling uncared for aOR [95% CI] *p*-value	Emotional violence aOR [95% CI] *p*-value
Change in caregiver’s gender attitudes (higher = more equitable)	1.08	1.07	1.02	1.03	1.03
	[1.005, 1.154] 0.037	[0.989, 1.158] 0.092	[0.927, 1.112] 0.746	[0.948, 1.111] 0.510	[0.946, 1.114] 0.524
Girl’s age	0.77	0.86	0.86	0.98	0.82
	[0.686, 0.861] <0.001	[0.757, 0.984] 0.028	[0.743, 1.003] 0.055	[0.849, 1.105] 0.636	[0.717, 0.942] 0.005
Caregiver’s age	1.00	0.99	1.00	1.00	1.00
	[0.997, 1.003] 0.975	[0.976, 1.012] 0.500	[0.998, 1.005] 0.338	[0.998, 1.004] 0.486	[0.989, 1.007] 0.567
Caregiver’s gender (female)	0.91	0.832	1.443	1.032	1.234
	[0.473, 1.745] 0.773	[0.398, 1.708] 0.605	[0.533, 3.889] 0.473	[0.497, 2.144] 0.932	[0.565, 2.697] 0.600

Note: Each column represents a different regression. Logistic regression was used to estimate adjusted odds ratios. Unadjusted odds ratios are presented in S1 Table. All models control for girl’s age, caregiver’s age, caregiver’s gender, treatment status, and village fixed effects.

Regression analyses also revealed that older girls had lower odds of experiencing an improvement in school participation (aOR = 0.77; 95% CI [0.686, 0.861]; *p* < 0.001), physical violence (aOR = 0.86; 95% CI [0.757, 0.984]; *p* = 0.028), or emotional violence (aOR = 0.82; 95% CI [0.717, 0.942]; *p* = 0.005). All findings remained robust to sensitivity analysis using imputed data (see S2 Table).

## Discussion

Overall, these analyses reveal that over time, adolescent girls’ school participation improved, their experiences of violence reduced, and caregiver gender-equitable attitudes improved. Changes observed in gender-equitable attitudes among caregivers were also associated with marginally significant reductions in physical violence against adolescent girls and significant improvements in school participation, while no statistically significant observed effects were noted for other outcomes. These findings build on the baseline analysis of these data that suggested that targeting caregivers’ gender-equitable attitudes may be a useful strategy as part of a comprehensive approach for improving school participation [51]. By detailing how improvements in gender-equitable attitudes continued to be associated with higher levels of school participation over time, these findings support this earlier work and align with previous research demonstrating that caregiver attitudes and support toward schooling are critical components of retention [55]. These approaches may be grafted to more traditional educational programming that seeks to improve student academic and psychosocial outcomes through establishing cooperative and supportive learning environments within school settings. Importantly, these findings continue to support the importance of addressing the home environment for student success, particularly for girl children, in addition to school and teacher-centered interventions seeking to improve educational outcomes that have been previously evaluated in DRC [56,57]. Holistic attention to actors throughout the ecological model beyond caregivers may be particularly important for adolescent girls in conflict-affected settings, given the increased barriers to retention due to displacement, instability or underinvestment, and poor quality of schools in these settings [58,59].

Further, addressing caregiver gender-equitable attitudes may also lead to overall decreased likelihood of violence, particularly physical violence, which may be perpetrated by a caregiver, partner, or other person. Potential impact of caregiver-focused programs for adolescent girls may be strengthened through the use of cash incentives [47], which may also improve participation in programming, cognitive bandwidth for caregivers, reduce stress, or operate through other potential pathways. It is also important to recognize that, given the known associations between community-level gender norms and risk of violence for women, holistic attention to actors throughout the ecological model is needed in order to induce sustainable change in outcomes for girls [60–64]. While the regression models in this study accounted for community influence through the inclusion of village-level fixed effects, further research could usefully examine the comparative impact of caregiver and community norms and attitudes on girls’ outcomes. Additionally, research is needed to understand how to maximize the potential for caregiver gender-equitable attitudes on improving the safety and well-being of adolescent girls as part of a broader strategy to influence community norms and males’ attitudes and behaviors in the community, particularly for sexual violence outcomes. A small but growing body of research is working to develop parenting programs to improve adolescent outcomes [65,66], but this remains a substantial knowledge gap in the violence prevention field [67].

It is also important to note that an improvement in gender-equitable attitudes was not associated with improvements in sexual violence, emotional violence, or feeling uncared for. While caregivers may be more likely to perpetrate physical abuse, sexual violence by caregivers (including male caregivers) has a different etiology. As such, it is perhaps not unsurprising that improvements in attitudes for caregivers were not associated with declines in sexual violence for girls. Similarly, male partners, who might be more likely to perpetrate IPV, would not necessarily be influenced by caregiver attitudes. Emotional violence, while sometimes perpetrated by caregivers, can also be committed by others, such as older siblings or peers [68]. Additional research is needed to understand the added value for girls of targeting gender attitudes among other family members and individuals involved in girls’ lives.

Notably, older girls were more likely to experience decreased odds of improved school participation and increases in violence between baseline and endline. This points to the importance of reaching girls at younger ages, as early as possible, to maximize potential effectiveness of violence prevention and educational retention programming. Simultaneously, given approximately 2 in 5 and 1 in 4 girls reported experiencing violence at baseline, findings point to the need for comprehensive response services to mitigate any negative impacts of experiencing violence. Future studies should examine for changes over time and effect modification of age. For example, a recent study in DRC found strengthened change for younger girls between 10 and 13 years as compared to older girls (14 to 15 years) on outcomes related to asset building through pathways of economic empowerment and increased opportunities for younger girls [69]. On the other hand, older adolescent girls were more likely to demonstrate higher school attendance than younger girls. Nuanced disaggregation and analysis of programming impacts are needed during this critical period of development.

Data presented in this study were from a randomized controlled trial evaluating the incremental impact of a caregiver component attached to an adolescent girl life skills program. Results from the parent trial revealed that this additional component did not confer additional reductions of sexual and other forms of violence or improvements in gender attitudes, though some caregiver parenting behaviors such as warmth toward their adolescent girl improved [49]. Limitations of this trial included an overlap of intervention delivery and follow-up period for the endline outcomes as well as evaluation of a program that was not fully piloted by programmatic teams before rigorous evaluation at the request of the donor. Additionally, it is important to note that girls chose the caregiver they wanted to participate in the program, of which more than 90% selected a female caregiver. It is reasonable to assume that improved gender attitudes among fathers may also confer additional benefits, but more research is needed to ascertain whether male caregivers’ attitudes are simply correlated with female caregivers’ attitudes, whether male caregivers drive their female partners’ attitudes or whether this causal relationship operates in the opposite direction. Further, this group-based program was randomized within communities that were not highly geographically dispersed in order to increase statistical power of the study. Given that the present longitudinal analysis demonstrates improvement in caregiver gender-equitable attitudes over time, possible spillover between caregiver groups who received the program and those who were waitlisted was possible. In addition, all adolescent girls participated in the life skills program. It is also possible that adolescent girls brought information home to discuss with their caregivers, as all girls were part of the program, which, in turn, improved caregiver gender-equitable attitudes, regardless of one’s own participation in the caregiver component of the program. Future studies should assess for potential spillover, including more robust process evaluations to triangulate findings. Additionally, while we note change over time in both schooling and gender attitudes, it is also plausible that caregiver’s attitudes on gender equality improved as their adolescent girls went to school, which may have also decreased their corporal punishment against girls.

Inferences from this study are limited due to a few main factors. First, data were only collected at 2 time points, and it remains unclear whether these trends attenuate or strengthen over time. Second, given the a priori hypothesis, the outcomes of interest were operationalized such that only detection of positive changes were possible. Future research might consider how regression in caregivers’ gender-equitable attitudes might lead to a worsening of outcomes for adolescent girls. Third, all outcomes were self-reported; results may be biased due to underreporting related to the sensitive nature of these outcomes. Fourth, the sample for this analysis was drawn using convenience sampling from community members who were interested in participating in a life skills program for adolescent girls. Selection bias may have been present in which girls and their caregivers already on the pathway to improved outcomes were more likely to want to participate in such programming, limiting our ability to generalize the findings more widely in DRC. Lastly, the outcome variables for feeling uncared for and for physical, emotional, and sexual violence were constructed from single items in the questionnaire. Given that a validated or normed scale that consists of multiple questions to assess exposure to violence was not used, the single keyword questions used to construct the outcomes may have underestimated the true occurrence of violence, potentially attenuating results toward the null.

## Conclusions

In summary, early intervention for adolescent girls and supporting caregivers to increase gender-equitable attitudes may be an important strategy to improve dual outcomes of educational and safety outcomes for adolescent girls in eastern DRC. Further research is critical to generate a better understanding of how to actually induce a shift in these attitudes in multisectoral programming that seeks to improve educational and safety outcomes for adolescent girls in humanitarian settings.

## Supporting information

S1 Strobe checklist Outline of items included in study and their location in the article. (DOCX)

S1 Table Estimating changes in girl’s outcomes, unadjusted odds ratios. (DOCX)

S2 Table Estimating changes in girl’s outcomes on imputed data, beta coefficients. (DOCX)

S1 Questionnaire Girls’ survey—French. (PDF)

S2 Questionnaire Girls’ survey—Mashi. (PDF)

S3 Questionnaire Girls’ survey—Swahili. (PDF)

S4 Questionnaire Caregivers’ survey—French. (PDF)

S5 Questionnaire Caregivers’ survey—Mashi. (PDF)

S6 Questionnaire Caregivers’ survey—Swahili. (PDF)

## Abbreviations

ACASI: audio computer-assisted self-interview
CAPI: computer-assisted personal interviewing
COMPASS: Creating Opportunities through Mentorship, Parental Involvement, and Safe Spaces
DRC: Democratic Republic of the Congo
IPV: intimate partner violence
STI: sexually transmitted infection
