# Supplementary material for: Gender-equitable caregiver attitudes and education and safety of adolescent girls in South Kivu, DRC: A secondary analysis from a randomized controlled trial
Source: PLoS Med. 2021 Sep 28;18(9):e1003619. doi: 10.1371/journal.pmed.1003619 (PMC8478225; doi:10.1371/journal.pmed.1003619)
Supplement: S1 Table — (DOCX) [file pmed.1003619.s002.docx]

| S1 Table. Estimating changes in girls' outcomes, unadjusted odds ratios | | | |  | |  | |  |
| --- | --- | --- | --- | --- | --- | --- | --- | --- |
|  | Improvement in: | | | | | | | |
|  | School participation  OR [95% CI] P-value | Physical violence  OR [95% CI] P-value | Sexual violence OR [95% CI]  P-value | | Feeling uncared for  OR [95% CI]  P-value | | Emotional violence  OR [95% CI]  P-value | |
| Change in caregiver’s gender attitudes (higher=more equitable) | 1.08 | 1.08 | 0.97 | | 1.03 | | 1.02 | |
|  | [1.018,1.151] | [1.006,1.164] | [0.897,1.058] | | [0.963,1.111] | | [0.943,1.097] | |
|  | 0.012 | 0.033 | 0.532 | | 0.359 | | 0.657 | |
| Girl’s age | 0.78 | 0.9 | 0.86 | | 0.99 | | 0.83 | |
|  | [0.707,0.864] | [0.801,1.012] | [0.747,0.982] | | [0.879,1.112] | | [0.736,0.940] | |
|  | <0.001 | 0.077 | 0.026 | | 0.848 | | 0.003 | |
| Caregiver’s age | 1.00 | 1.00 | 1.00 | | 1.00 | | 1.00 | |
|  | [0.997,1.003] | [0.988,1.006] | [0.998,1.005] | | [0.998,1.004] | | [0.983,1.009] | |
|  | 0.933 | 0.518 | 0.394 | | 0.498 | | 0.515 | |
| Caregiver’s gender (female) | 1.00 | 0.95 | 1.63 | | 1.18 | | 1.17 | |
|  | [0.558,1.780] | [0.482,1.878] | [0.624,4.248] | | [0.589,2.382] | | [0.570,2.413] | |
|  | 0.99 | 0.886 | 0.32 | | 0.634 | | 0.665 | |
| Note: Each column represents a different regression. Logistic regression was used to estimate odds ratios. All models control for girl’s age, caregiver’s age, caregiver’s gender, treatment status, and village fixed effects. | | | | | | | |  |
