# Supplementary material for: Gender-equitable caregiver attitudes and education and safety of adolescent girls in South Kivu, DRC: A secondary analysis from a randomized controlled trial
Source: PLoS Med. 2021 Sep 28;18(9):e1003619. doi: 10.1371/journal.pmed.1003619 (PMC8478225; doi:10.1371/journal.pmed.1003619)
Supplement: S2 Table — (DOCX) [file pmed.1003619.s003.docx]

| S2 Table. Estimating changes in girls' outcomes on imputed data, Beta coefficients | | | | | |  |
| --- | --- | --- | --- | --- | --- | --- |
|  | Improvement in: | | | | | |
|  | School participation  B [95% CI] P-value | Physical violence  B [95% CI] P-value | Sexual violence  B [95% CI]  P-value | Feeling uncared for  B [95% CI]  P-value | Emotional violence  B [95% CI]  P-value | |
| Change in caregiver’s gender attitudes (higher=more equitable) | 0.075 | 0.061 | 0.016 | 0.033 | 0.016 | |
|  | [0.006,0.145]  0.034 | [-0.018,0.139]  0.128 | [-0.072,0.104]  0.721 | [-0.043,0.109]  0.394 | [-0.061,0.093]  0.682 | |
| Girl’s age | -0.230 | -0.105 | -0.146 | -0.014 | -0.167 | |
|  | [-0.344,-0.116]  <0.001 | [-0.228,0.017]  0.092 | [-0.288,-0.004]  0.045 | [-0.146,0.118]  0.832 | [-0.311,-0.022]  0.025 | |
| Caregiver’s age | 0.002 | -0.004 | 0.002 | 0.001 | -0.004 | |
|  | [-0.003,0.004]  0.891 | [-0.016,0.009]  0.564 | [-0.002,0.005]  0.304 | [-0.002,0.004]  0.534 | [-0.017,0.009]  0.552 | |
| Caregiver’s gender (female) | -0.148 | -0.099 | 0.427 | 0.119 | 0.272 | |
|  | [-0.778,0.409]  0.542 | [-0.796,0.597]  0.779 | [-0.461,1.315]  0.345 | [-0.568,0.807]  0.733 | [-0.472,1.016]  0.473 | |
| Note: Each column represents a different regression. Logistic regression was used and findings are reported as Beta coefficients. All models control for girl’s age, caregiver’s age, caregiver’s gender, treatment status, and village fixed effects. | | | | | |  |
