# Supplementary material for: Gender-equitable caregiver attitudes and education and safety of adolescent girls in South Kivu, DRC: A secondary analysis from a randomized controlled trial
Source: PLoS Med. 2021 Sep 28;18(9):e1003619. doi: 10.1371/journal.pmed.1003619 (PMC8478225; doi:10.1371/journal.pmed.1003619)
Supplement: S1 Questionnaire — (PDF) [file pmed.1003619.s004.pdf]

**Girls' survey- French**

| Girl survey (DRC) | | | |
|---|---|---|---|
| **Question #** | **Question** | **Response options** | **Instructions** |
| **A. Questions administratives** | | | |
| A1 | Nom du village/site | _________________ | Record |
| A2 | Quartier | _________________ | Record from listing |
| A3 | Sous village du quartier | _________________ | Record from listing |
| A4 | Code d'enquêteur | ___________ | Record your ID here |
| A5 | Date de l'enquête | ________/________/________ ____ ____ | jj/mm/aaaa |
| A6 | Heure du début de l'enquête | ___________: ___________ | 24 heures |
| A7 | Heure de la fin de l'enquête | ___________: ___________ | 24 heures |
| A8 | Code d'identification du parent | _______________ | |
| A9 | Code d'identification de la fille | ________________ | |
| A10 | Age de la fille | | |
| **B. Informations Démographiques** | **Bonjour encore, et merci pour nous rencontrer pour cette interview. J'aimerais vous rappeler au sujet de ce que nous avons discuté la dernière fois qu'on s'est parlé. Nous allons vous poser des questions au sujet de votre vie et vos expériences, y compris votre famille, votre école (si vous êtes a l'école), votre sécurité, vos relations avec les autres filles et garçons, et vos espoirs pour l'avenir. Bien que vous ne bénéficiera pas personnellement de votre participation dans cette interview, ni recevra de l'argent ou autres avantages matériels, nous récoltons cette information pour améliorer des programmes pour les filles en DRC, et nous espérons que vous serez réfléchi et honnête en répondant.**<br><br>**Il n'y a aucune pression sur vous pour nous parler aujourd'hui. Vous pouvez dire que vous ne voulez pas nous parler aujourd'hui et nous ne serons pas offensés. Vous pouvez également refuser de répondre à toute question à la quelle vous ne voulez pas répondre si vous vous sentez gêné ou mal à l'aise, et rien de mauvais ne vous arrivera; nous passerons tout simplement à la question suivante.**<br><br>**L'information que vous nous donnerez sera utilisée exclusivement pour le but de la recherche, et personne (y compris votre famille, amis, ou autres personnes dans votre communauté) ne saura ce que vous avez dit. Tout ce que vous nous dites sera gardé en secret, et nous allons utiliser une ordinateur pour s'assurer que même moi je ne saurai pas certaines de vos réponses.**<br><br>**Avant que nous commencions, je veux m'assurer que vous êtes a l'aise et que vous comprenez tout au sujet de ce que nous avons parlé. Est-ce que vous avez des questions? Est-ce que vous consentez à me parler aujourd'hui?**<br><br>**Génial. Nous commençons avec des questions faciles au sujet de vous.** | | |
| B1 | Êtes-vous deja allé à l'école? | 1=Oui<br>2=Non<br>888=Ne sait pas | Si « Non » ou « ne sais pas », passer à B8 |

| Girl survey (DRC) | | | |
|---|---|---|---|
| **Question #** | **Question** | **Response options** | **Instructions** |
| | | 999 = Pas de réponse | |
| B3 | Quel était le niveau le plus élevé que vous avez atteint à l'école? | _____ _____<br><br>888=Ne sait pas<br>999 = Pas de réponse | |
| B4 | Etiez-vous inscrite à l'école durant la dernière année scolaire (2015-2016)? | 1=Oui<br>2=Non<br>888=Ne sait pas<br>999=Pas de réponse | Si oui, procéder à B6<br>Si non, passer à B5 |
| B5 | Quelle est la raison principale pour laquelle vous ne puissiez-pas être inscrite à l'école pendant l'année scolaire la plus récente année (2015-2016)? | 1=La famille n'avait pas les moyens,<br>2=S'est mariée,<br>3=Trop des responsabilités domestiques<br>4=L'école était trop loin/y'avait pas d'école dans les environs,<br>5=La famille n'a pas approuvé/n'y a pas trouvé de bénéfice,<br>5=Y'avait pas des places disponible à l'école (l'école était pleine)<br>7=Etait enceinte,<br>7=Terminé l'école,<br>8=Echoué à l'école<br>10=Autre<br>888=Ne sait pas<br>999 = Pas de réponse | Passer à B8 |
| B6 | Pendant la dernière semaine de classe, aviez-vous raté des jours? Si oui, combien? | 0=0<br>1=1<br>2=2<br>3=3<br>4=4<br>5=5<br>6=6<br>888=Ne sait pas<br>999 = Pas de réponse | |
| B7 | La dernière fois que vous étiez absente à l'école, pourquoi n'y êtes-vous pas allée? | 1=Malade,<br>2=Je devais faire les travaux ménagers,<br>3=Je devais m'occuper d'un enfant ou d'un malade,<br>4=Je devais aller travailler pour gagner la vie,<br>5=J'étais trop fatiguée,<br>6=J'avais de plans avec les amis,<br>7=Je ne voulais pas y aller,<br>8=Menstruation,<br>9=Autre<br>888=Ne sait pas<br>999 = Pas de réponse | Si « Autre », preciser |
| B8 | A part vos travaux ménagers de routine, avez-vous déjà travaillé pour gagner de l'argent ou autre forme de paiement? | 1=Oui<br>2=Non<br>888=Ne sait pas<br>999 = Pas de réponse | Si « non » ou « ne sait pas, passer à B10 |

| Girl survey (DRC) | | | |
|---|---|---|---|
| **Question #** | **Question** | **Response options** | **Instructions** |
| B9 | Quelle sorte de travail avez-vous réalisé dans les 12 mois passés pour gagner de l'argent ou autres paiements ? | 1=Aucun,<br>2=Travail dans un magasin/boutique,<br>3=Des travaux ménagers pour une autre famille,<br>4=S'occuper du bébé d'une autre famille,<br>5=Travaux de champ<br>6=Construction<br>7=Faire des objets artisanaux<br>8=La collecte les bois de chauffage<br>9=Petit commerce<br>10=Autre<br>888=Ne sait pas<br>999 = Pas de réponse | Coche tout ce qui s'applique |
| B10 | A part vos travaux ménagers de routine, avez-vous fait d'autre travail dans les 12 mois passés pour lequel on ne vous a pas payé? | 1=Aucun<br>2=travail dans un magasin/boutique<br>3=des travaux ménagers pour une autre famille<br>4=s'occuper du bébé d'une autre famille<br>5=travaux de champ<br>6=construction<br>7=autre<br>888=Ne sait pas<br>999 = Pas de réponse | Coche tout ce qui s'applique |
| B11 | Maintenant, je voudrais vous poser quelques questions à propos de vos parents biologiques, Lequel de tes parents biologique vit avec toi ? | 1= Père<br>2=Mère<br>3 = Les Deux<br>4 = Aucun<br>888=Ne sait pas<br>999 = Pas de réponse | |
| **C. Relations avec les parents et autres adultes** | **Vous faites un bon travail!**<br><br>**Pour les prochaines deux questions, je vais dire quelque chose et j'aimerais que vous me disiez si c'est vraiment vrai, un peu vrai, ou pas de tout vrai. Ca va ci vous ne savez pas ou vous ne voulez pas repondre. Est-ce que vous comprennez ?** | | |
| C1 | Il y a un adulte dans votre vie qui vous donne les conseils.<br><br>Diriez-vous que cette asserstion est…. | 3=Vraiment vrai<br>2=Un peu vrai<br>1=Pas du tout vrai<br>888=Ne sait pas<br>999 = Pas de réponse | |
| C2 | Il y a un adulte dans votre vie que vous considérez comme un mentor ou encadreur.<br><br>Par mentor/encadreur, je veux dire une personne qui vous oriente, qui vous soutient moralement, et vous donne des bons conseils qui vous aident à avoir du succès dans la vie. | 3=Vraiment vrai<br>2=Un peu vrai<br>1=Pas du tout vrai<br>888=Ne sait pas<br>999 = Pas de réponse | Si « pas de tout vrau » ou « ne sais pas », passer a C5 |

| Girl survey (DRC) | | | |
|---|---|---|---|
| **Question #** | **Question** | **Response options** | **Instructions** |
| | Diriez-vous que cette asserstion est…. | | |
| C3 | Qui considérez-vous comme votre mentor ou encadreur ? | 1=Maman<br>2=Papa<br>3=Grand-mère<br>4=Grand-père<br>5=Sœur<br>6=Frère<br>7=Tante<br>8=Oncle<br>9=Mari<br>10=Copain<br>11=Autre adulte<br>12=Leader du programme avec les filles<br>888=Ne sait pas<br>999 = Pas de réponse | |
| C4 | A propos de quoi vous entretenez-vous avec votre mentor/encadreur? | 1=Projets d'avenir<br>2=Problèmes de santé<br>3=Leçons de l'école<br>4=Pairs à l'école<br>5=Problèmes familiaux<br>6=Conflits avec vos parents/responsables<br>7=Conflits avec votre mari, hommes, ou copains<br>8=Conflits avec les amis et les voisins<br>9=Conflits avec ses frères et sœurs<br>10=Conflits au travail<br>11=Plan financier/caisse d'épargne<br>12=Projet professionnel<br>13=Projets que vous avez avec votre copain ou mari<br>888=Ne sait pas<br>999 = Pas de réponse | Ne pas lire toutes les réponses, juste cocher toutes les réponses mentionnées par l'enquêtée |
| | **Ensuite je vais vous demander comment vous vous sentez en parlant de certains sujets spécifiques avec votre parent/responsable qui est inscrit dans le programme. Pour chaquevvsujet que je dit, je veux que vous me disiez si vous vous sentez a l'aise ou mal a l'aise en parler de ce sujet avec votre parent(s)/responsables(s). Ca va si vous ne savez pas ou vous ne voulez pas répondre. Est-ce que vous comprenez?** | | |
| C5a | Est-ce que vous etes a l'aise ou mal a l'aise en parlent au sujet de vos etudes avec vos parent(s)/responsable(s) ? | 1=à l'aise<br>2=mal à l'aise<br>888=Ne sait pas<br>999 = Pas de réponse | |

| Girl survey (DRC) | | | |
|---|---|---|---|
| **Question #** | **Question** | **Response options** | **Instructions** |
| C5b | Et au sujet de comment vous allez gagner votre vie dans l'avenir ? | 1=à l'aise<br>2=mal à l'aise<br>888=Ne sait pas<br>999 = Pas de réponse | |
| C5c | Et au sujet du mariage et quand vos parents / responsables envisagent que vous allez vous marier ? | 1=à l'aise<br>2=mal à l'aise<br>888=Ne sait pas<br>999 = Pas de réponse | |
| C5d | Et au sujet de la puberté (c.a.d les changements qui s'observent chez les enfants quand ils grandissent) | 1=à l'aise<br>2=mal à l'aise<br>888=Ne sait pas<br>999 = Pas de réponse | |
| C5e | Est-ce que vous etes a l'aise ou mal a l'aise en parlent des sujets relatifs au sexe avec vos parent(s)/responsable(s) ? | 1=à l'aise<br>2=mal à l'aise<br>888=Ne sait pas<br>999 = Pas de réponse | |
| C5f | Et au sujet de comment faire pour éviter de tomber enceinte ? | 1=à l'aise<br>2=mal à l'aise<br>888=Ne sait pas<br>999 = Pas de réponse | |
| C5g | Et au sujet du VIH/SIDA ou d'autres maladies sexuellement transmissibles ? | 1=à l'aise<br>2=mal à l'aise<br>888=Ne sait pas<br>999 = Pas de réponse | |
| **D. Sécurité** | **Bon travail jusqu'à maintenant ! Les questions suivantes sont relatives à vos sentiments à propos de votre sécurité : dans votre vie, votre communauté et les endroits où vous vous sentez en sécurité.** | | |
| D1 | Vous sentez-vous en sécurité à la maison? | 1=Oui<br>2=Non<br>888=Ne sait pas<br>999=Pas de réponse | |
| D2 | Vous sentez-vous en sécurité à l'école? | 1=Oui<br>2=Non<br>888=Ne sait pas<br>999=Pas de réponse | Si elles ont dit non à la question B2, ne posez pas cette question |
| D3 | Vous sentez-vous en sécurité chez un ami? | 1=Oui<br>2=Non<br>888=Ne sait pas<br>999=Pas de réponse | |
| D4 | Vous sentez-vous en sécurité chez un voisin? | 1=Oui<br>2=Non<br>888=Ne sait pas<br>999=Pas de réponse | |

| Girl survey (DRC) | | | |
|---|---|---|---|
| **Question #** | **Question** | **Response options** | **Instructions** |
| D5 | Y'a-t-il dans la communauté un endroit où vous vous sentez à l'aise pour rencontrer les autres filles? | 1=Oui<br>2=Non<br>888=Ne sait pas<br>999=Pas de réponse | |
| | **Maintenant, je voudrais vous poser quelques questions à propos de vos amis. Je vais dire certaines assertions ; pour chaqu'une, merci de me dire si vous etes d'accord ou pas d'accord. Ca va si vous ne savez pas ou vous ne voulez pas répondre. Est-ce que vous comprenez?** | | |
| D6 | J'ai des amis avec qui je peux parler des choses importantes. Etes-vous d'accord ou pas d'accord? | 1=D'accord<br>2=Pas d'accord<br>888=Ne sait pas<br>999=Pas de réponse | |
| D7 | J'ai des amis sur qui je peux compter pour un soutien emotionel. | 1=D'accord<br>2=Pas d'accord<br>888=Ne sait pas<br>999=Pas de réponse | |
| D8 | Avez-vous des amies du sexe féminin de votre âge hors de la famille ? | 1=Oui<br>2=Non<br>888=Ne sait pas<br>999=Pas de réponse | Si "non" ou « ne sait pas », passer à D10 |
| D9 | Combien de ces amies du sexe féminin avez-vous? | 0=0<br>1=1-3 amies<br>2=4-10 amies<br>3=Plus de 10 amies<br>888=Ne sait pas<br>999=Pas de réponse | |
| D10 | A part quelqu'un de votre famille, y-a-t-il dans la communauté un personnage féminin vers qui vous pouvez aller régulièrement raconter vos problèmes ? | 1=Oui<br>2=Non<br>888=Ne sait pas<br>999=Pas de réponse | |
| **E. Attitudes et aspirations** | **Maintenant, je vais vous poser quelques questions à propos de ce qui arrive à une fille pendant sa vie, comme l'école et le mariage.** | | |
| E1 | Quel niveau scolaire une fille devrait-elle accomplir avant de quitter l'école ? | ___________(niveau scolaire)<br>888=Ne sait pas<br>999=Pas de réponse | |
| E2 | A quel âge pensez-vous qu'une fille devrait se marier? | ___________(ans)<br>888=Ne sait pas<br>999=Pas de réponse | |
| E3 | A quel âge pensez-vous qu'une fille devrait accoucher de son premier bébé? | ___________(ans)<br>888=Ne sait pas<br>999=Pas de réponse | |
| **G. Estime de soi** | **Bienvenu à utiliser la tablette ACASI. Je m'appelle… ........................ et je vais vous lire les questions. Souvenez-vous que vous pouvez appeler l'enquêteur a n'importe quelle moment si vous avez des questions ou des problèmes avec l'ordinateur/ la tablette.**<br><br>**En premier, je vais vous lire 10 déclarations qui pourraient s'appliquer à vous peut-être. S'il vous plaît dites-moi combien vous êtes d'accord avec chaque déclaration: si vous êtes fortement d'accord, d'accord, en désaccord ou fortement en désaccord. Ca va si vous ne savez pas ou si vous ne voulez pas répondre. Est-ce que vous comprenez?** | | |

| Girl survey (DRC) | | | |
|---|---|---|---|
| **Question #** | **Question** | **Response options** | **Instructions** |
| | | | |
| G1 | Je me sens que je suis egale aux autres. | 4= Fortement d'accord<br>3=D'accord<br>2=Pas d'accord<br>1=Fortement pas d'accord<br>888=Ne sait pas<br>999=Pas de réponse | |
| G2 | Je sens que j'ai plusieurs bonnes qualités. | 4= Fortement d'accord<br>3=D'accord<br>2=Pas d'accord<br>1=Fortement pas d'accord<br>888=Ne sait pas<br>999=Pas de réponse | |
| G3 | Je suis disposée à sentir que je suis un échec. | 4= Fortement d'accord<br>3=D'accord<br>2=Pas d'accord<br>1=Fortement pas d'accord<br>888=Ne sait pas<br>999=Pas de réponse | |
| G4 | Je suis capable de faire des choses aussi bien que la plupart des autres personnes. | 4= Fortement d'accord<br>3=D'accord<br>2=Pas d'accord<br>1=Fortement pas d'accord<br>888=Ne sait pas<br>999=Pas de réponse | |
| G5 | Je sens que j'ai des raisons d'être fier de moi-même. | 4= Fortement d'accord<br>3=D'accord<br>2=Pas d'accord<br>1=Fortement pas d'accord<br>888=Ne sait pas<br>999=Pas de réponse | |
| G6 | J'ai une attitude positive vers moi-même. | 4= Fortement d'accord<br>3=D'accord<br>2=Pas d'accord<br>1=Fortement pas d'accord<br>888=Ne sait pas<br>999=Pas de réponse | |
| G7 | Je suis satisfaite de moi-même. | 4= Fortement d'accord<br>3=D'accord<br>2=Pas d'accord<br>1=Fortement pas d'accord<br>888=Ne sait pas<br>999=Pas de réponse | |
| G8 | J'ai beaucoup de respect pour moi-même. | 4= Fortement d'accord<br>3=D'accord<br>2=Pas d'accord<br>1=Fortement pas d'accord<br>888=Ne sait pas<br>999=Pas de réponse | |
| G9 | Je me sens suis une personne utile. | 4= Fortement d'accord<br>3=D'accord<br>2=Pas d'accord | |

| Girl survey (DRC) | | | |
|---|---|---|---|
| **Question #** | **Question** | **Response options** | **Instructions** |
| | | 1=Fortement pas d'accord<br>888=Ne sait pas<br>999=Pas de réponse | |
| G10 | Je pense que je suis une personne de valeur. | 4= Fortement d'accord<br>3=D'accord<br>2=Pas d'accord<br>1=Fortement pas d'accord<br>888=Ne sait pas<br>999=Pas de réponse | |
| **I. Attitudes concernant la Violence Domestique** | **Parfois le mari est énervé par certaines choses que fait sa femme. Je vais vous lire quelques situations. Pour chac'une de ces situations, j'aimerais que vous me disiez si vous pensez que le mari a raison de frapper sa femme. Rappelez-vous qu'il n'y a pas de bonnes ou de mauvaises réponses et que vous pouvez dire que vous ne savez pas ou que vous ne voulez pas répondre à n'importe quel moment.** | | |
| I1 | Est-il acceptable pour un mari de fapper sa femme si elle sort sans lui dire ? | 1=Oui<br>2=Non<br>888=Ne sait pas<br>999=Pas de réponse | |
| I2 | Est-il acceptable pour un mari de fapper sa femme si elle ne s'occupe pas bien de ses enfants? | 1=Oui<br>2=Non<br>888=Ne sait pas<br>999=Pas de réponse | |
| I3 | Est-il acceptable pour un mari de fapper sa femme si elle se dispute avec lui? | 1=Oui<br>2=Non<br>888=Ne sait pas<br>999=Pas de réponse | |
| I4 | Est-il acceptable pour un mari de fapper sa femme si elle refuse de faire des rapports sexuels avec lui? | 1=Oui<br>2=Non<br>888=Ne sait pas<br>999=Pas de réponse | |
| I5 | Est-il acceptable pour un mari de fapper sa femme si elle brule la nourriture? | 1=Oui<br>2=Non<br>888=Ne sait pas<br>999=Pas de réponse | |
| **J. Violence physique et l'abus émotionnel et verbal** | **La série suivante des questions, il s'agit des autres choses qui pourraient vous être arrivées. Nous comprenons que certaines de ces questions sont personnelles et pourraient vous mettre mal à l'aise, mais nous espérons que vous partagez des informations avec nous pour que nous puissions mieux comprendre les besoins des filles comme vous-même. Gardez en tête que votre nom n'est pas écrit sur l'enquête et comme vous utilisez cet ordinateur aucune autre personne ne saura vos réponses. Souvenez-vous que les choses faites à vous ne sont pas de votre faute. Sentez-vous à l'aise de répondre ouvertement et honnêtement et vous n'êtes pas obligé de répondre à une question si vous ne vous sentez pas à l'aise.** | | |
| J1 | Dans les 12 derniers mois, est-ce que quelqu'un vous a déjà frappé ou claqué pour vous faire mal? | 1=Oui<br>2=Non<br>888=Ne sait pas<br>999=Pas de réponse | Si « non » ou « ne sais pas », passer J3 |
| J2 | Qui vous a blessé de cette manière? | 1=Copain ou mari<br>2=Parent ou responsable<br>3=Autre membre de la famille<br>4=Ami ou voisin<br>5=Membre d'un groupe armé | Cochez TOUT ce qui convient |

| Girl survey (DRC) | | | |
|---|---|---|---|
| **Question #** | **Question** | **Response options** | **Instructions** |
| | | 6=Authorité<br>7=Autre<br>999=Pas de réponse | |
| J3 | Dans les 12 mois passés, est-ce que quelqu'un a crié ou hurlé sur vous d'une manière forte ou agressive? | 1=Oui<br>2=Non<br>888=Ne sait pas<br>999=Pas de réponse | Si « non » ou « ne sait pas », passer a J5 |
| J4 | Qui a crié sur vous d'une manière forte ou agressive? | 1=Copain ou mari<br>2=Parent ou responsable<br>3=Autre membre de la famille<br>4=Ami ou voisin<br>5=Membre d'un groupe armé<br>6=Authorité<br>7=Autre<br>999=Pas de réponse | Cochez TOUT ce qui convient |
| J5 | Dans les 12 mois passés, est ce que quelqu'un vous a insulté, vous a dit des mauvaises choses, ou vous a maudit? | 1=Oui<br>2=Non<br>888=Ne sait pas<br>999=Pas de réponse | Si « non » ou « ne sait pas », passer a J7 |
| J6 | Qui vous a insulté, vous a dit des mauvaises choses, ou vous a maudit? | 1=Copain ou mari<br>2=Parent ou responsable<br>3=Autre membre de la famille<br>4=Ami ou voisin<br>5=Membre d'un groupe armé<br>6=Authorité<br>7=Autre<br>999=Pas de réponse | Cochez TOUT ce qui convient |
| J7 | Dans les 12 mois passés, avez-vous vous senti que celui qui devrait s'occuper de vous ne s'occupait pas de vous ? | 1=Oui<br>2=Non<br>888=Ne sait pas<br>999=Pas de réponse | Si « non » ou « ne sait pas », passer a la section K |
| J8 | Dans les 12 mois passés, avec quelle fréquence avez-vous senti qu'on ne s'occupait pas de vous? | 1=Beaucoup de fois<br>2=Quelques fois<br>3=Jamais<br>888=Ne sait pas<br>999=Pas de réponse | |
| **K. Violence Sexuelle** | **Merci pour avoir répondu à ces questions au sujet de choses que vous avez expérimentées. Je sais que répondre à cette sorte de question n'est pas facile. La prochaine section a aussi des questions difficiles, mais nous espérons qu'en utilisant cet ordinateur/cette tablette vous vous sentez à l'aise pour nous dire ce que vous avez expérimenté.**<br><br>**Les filles et femmes peuvent expérimenter le contact sexuel sans leur consentement par les gens qu'elles connaissent bien, tel qu'un partenaire romantique, membre de la famille ou ami, ou par les étrangers. Les questions dans ces sections sont personnelles, et peuvent être inconfortables à répondre, mais vous nous aiderez à comprendre l'expérience des gens de contact sexuel sans leur autorisation. Vos réponses sont confidentielles et vous pouvez sauter toutes questions que vous préférez ne pas répondre. Aussi, souvenez-vous que vous ne serez pas jugé et qu'il n'y a pas de bonnes ou mauvaises réponses.** | | |

| Girl survey (DRC) | | | |
|---|---|---|---|
| **Question #** | **Question** | **Response options** | **Instructions** |
| | **Souvenez-vous que tout ce qui a été fait sur vous sans votre autorisation explicite ou fait par un adulte n'est pas votre faute.** | | |
| K1 | Est-ce quelqu'un vous jamais a touché d'une façon sexuelle sans votre permission ?<br><br>Toucher de façon sexuelle inclus caresser, pincer, empoigner. | 1=Oui<br>2=Non<br>888=Ne sait pas<br>999=Pas de réponse | Si « non » ou « ne sais pas », passer a K4 |
| K2 | Qui a fait ça? | 1=Copain ou mari<br>2=Parent ou responsable<br>3=Autre membre de la famille<br>4=Ami ou voisin<br>5=Membre d'un groupe armé<br>6=Authorité<br>7=Autre<br>999=Pas de réponse | Cochez TOUT ce qui convient |
| K3 | Combien de fois dans les 12 mois passees quelqu'un vous touché d'une façon sexuelle sans votre permission ? | ____ _____<br><br>888=Ne sait pas<br>999=Pas de réponse | |
| K4 | Est-ce que quelqu'un a déjà essayer d'utiliser son influence ou son athorite de vous menacer ou faire pression sur vous de faire les rapports sexuels avec lui?<br><br>Par exemple, en disant qu'il/elle vous donnera une mauvaise note à l'école, ou qu'il/elle va faire quelque chose de mauvaise sur vous ? | 1=Oui<br>2=Non<br>888=Ne sait pas<br>999=Pas de réponse | Si « non » ou « ne sais pas », passer a K7 |
| K5 | Qui a fait ça? | 1=Copain ou mari<br>2=Parent ou responsable<br>3=Autre membre de la famille<br>4=Ami ou voisin<br>5=Membre d'un groupe armé<br>6=Authorité<br>7=Autre<br>999=Pas de réponse | Cochez TOUT ce qui convient |
| | Dans les 12 derniers mois, avez-vous eu des rapports sexuels avec quelqu'un parcequ'il/elle vous a menace ou fait pression sur vous en utilisant son influence ou son athorite? | 1=Oui<br>2=Non<br>888=Ne sait pas<br>999=Pas de réponse | |
| K6 | Dans les 12 derniers mois, combien de fois avez-vous eu des rapports sexuels | _____ _____ | |

| Girl survey (DRC) | | | |
|---|---|---|---|
| **Question #** | **Question** | **Response options** | **Instructions** |
| | avec quelqu'un parcequ'il/elle vous a menace ou fait pression sur vous en utilisant son influence ou son athorite? | 888=Ne sait pas<br>999=Pas de réponse | |
| K7 | Avez-vous déjà eu des rapports sexuels avec un homme avec qui vous habitez afin que vous puissiez avoir de l'argent ou autes choses comme la nourriture ? | 1=Oui<br>2=Non<br>888=Ne sait pas<br>999=Pas de réponse | Si « non » ou « ne sait pas » passer a K9 |
| K8 | Est-ce que ceci vous est arrivé dans les 12 mois passés? | 1=Oui<br>2=Non<br>888=Ne sait pas<br>999=Pas de réponse | |
| K9 | Quelqu'un vous a-t-il déjà donné de l'argent, à manger, des cadeaux ou n'importe quelle autre faveur en échange pour faire des rapports sexuels avec lui ? | 1=Oui<br>2=Non<br>888=Ne sait pas<br>999=Pas de réponse | Si « non » ou « ne sais pas », passer a K11 |
| K10 | Est-ce que ceci vous est arrivé dans les 12 mois passés? | 1=Oui<br>2=Non<br>888=Ne sait pas<br>999=Pas de réponse | |
| | **S'il vous plait, dites-moi si vous êtes d'accord ou pas d'accord avec chacune des déclarations suivantes. Rappelez-vous qu'il n'y a pas de bonnes ou mauvaises réponses et c'est acceptable si vous ne connaissez pas la réponse ou si vous ne voulez pas répondre.** | | |
| K11 | Ma famille me blamerait si j'etais forcé d'avoir les relations sexuels | 1=D'accord<br>2=Pas d'accord<br>888=Ne sait pas<br>999=Pas de réponse | |
| K12 | Ma communauté me forcerait de me marier à un homme s'il m'a forcé de faire des rapports sexuels avec lui | 1=D'accord<br>2=Pas d'accord<br>888=Ne sait pas<br>999=Pas de réponse | |
| K13 | J'ai quelqu'un dans la communauté en qui je peux faire confiance et vers qui j'irais parler si j'etais forcé à avoir de rapports sexuels | 1=D'accord<br>2=Pas d'accord<br>888=Ne sait pas<br>999=Pas de réponse | |
| **L. Services** | **Merci pour avoir répondu à ces questions difficiles, vous avez fait un excellent travail. Je vous promets qu'il n'y aura plus de questions comme cela au sujet de choses que vous avez expérimenté. Maintenant je veux vous poser des questions au sujet de services qui peuvent être disponible aux filles comme vous-meme.** | | |
| L1 | Si quelqu'un a eu ou a tenté d'avoir les relations sexuelles avec une fille quand elle ne le voulait pas, connaissez-vous un endroit où elle peut aller pour chercher de l'aide? | 1=Oui<br>2=Non<br>888=Ne sait pas<br>999=Pas de réponse | |
| L2 | Connaissez-vous un endroit où une fille peut aller si quelqu'un l'a frappé ? | 1=Oui<br>2=Non<br>888=Ne sait pas<br>999=Pas de réponse | |

| Girl survey (DRC) | | | |
|---|---|---|---|
| **Question #** | **Question** | **Response options** | **Instructions** |
| **M. Espoir et orientation futures** | **Maintenant je vais lire des déclarations sur comment vous pensez au sujet de vous-même. Je veux que vous me disiez pour chacun la fréquence avec laquelle vous pensez de cette manière - si vous ne pensez jamais comme ça, ou un peu du temps, quelques fois, souvent, ou tous le temps.**<br><br>**Comme toujours, souvenez-vous qu'il n'y a pas des bonnes ou mauvaises réponses et vous pouvez dire que vous ne savez pas ou refuser de répondre à toute question.** | | |
| M1 | Je pense que je me porte bien.<br><br>Avec quelle frequence pensez-vous de cette manière ? | 1=Jamais<br>2=Peu de temps<br>3=Quelques fois<br>4=Souvent<br>5=Tous le temps<br>888=Ne sait pas<br>999=Pas de réponse | |
| M2 | Je peux penser à plusieurs voies d'obtenir les choses qui sont les plus importantes pour moi dans la vie.<br><br>Avec quelle frequence pensez-vous de cette manière ? | 1=Jamais<br>2=Peu de temps<br>3=Quelques fois<br>4=Souvent<br>5=Tous le temps<br>888=Ne sait pas<br>999=Pas de réponse | |
| M3 | J'ai le meme capacite de reussir que les autres filles de mon âge.<br><br>Avec quelle frequence pensez-vous de cette manière ? | 1=Jamais<br>2=Peu de temps<br>3=Quelques fois<br>4=Souvent<br>5=Tous le temps<br>888=Ne sait pas<br>999=Pas de réponse | |
| M4 | Quand j'ai un problème je peux trouver plusieurs façons de le résoudre.<br><br>Avec quelle frequence pensez-vous de cette manière ? | 1=Jamais<br>2=Peu de temps<br>3=Quelques fois<br>4=Souvent<br>5=Tous le temps<br>888=Ne sait pas<br>999=Pas de réponse | |
| M5 | Je pense que les choses que j'ai fait dans le passé m'aideront dans le futur.<br><br>Avec quelle frequence pensez-vous de cette manière ? | 1=Jamais<br>2=Peu de temps<br>3=Quelques fois<br>4=Souvent<br>5=Tous le temps<br>888=Ne sait pas<br>999=Pas de réponse | |
| M6 | Même quand les autres veulent renoncer, je sais que je peux trouver les voies pour résoudre un problème.<br><br>Avec quelle frequence pensez-vous de cette manière ? | 1=Jamais<br>2=Peu de temps<br>3=Quelques fois<br>4=Souvent<br>5=Tous le temps<br>888=Ne sait pas<br>999=Pas de réponse | |
| **N. Questions de Fin** | **Merci pour avoir répondu à ces questions. Je sais que quelques-unes d'entre elles avaient été difficiles. Souvenez-vous que personne dans votre communauté ne saura jamais ce que vous avez répondu. C'est la fin d'utilisation de la tablette, s'il vous plaît appelez l'enquêteur et remets-le à** | | |

| Girl survey (DRC) | | | |
|---|---|---|---|
| **Question #** | **Question** | **Response options** | **Instructions** |
| | **eux.**<br><br>**(UNE FOIS L'ENFANT A TERMINE, REPRENEZ LA TABLETTE ACASI. VERIFIEZ SI TOUTES LES CHOSES SONT CORRECTES ET ALORS LIT LA DÉCLARATION EN DESSOUS:)**<br><br>**Merci pour avoir répondu aux questions. Vous avez fait un bon travail en utilisant l'ordinateur et en répondant au sujet des questions difficiles.**<br><br>**Je vais maintenant vous poser quelques questions plus faciles au sujet de ce que vous aimez faire.** | | |
| N1 | Quelle est votre activité préférée à faire dans votre village? | | |
| **O. Questions de Conclusion Pour l'Enquêtée** | **Nous avons presque terminé. Avant de terminer notre discussion, j'ai quelques autres questions pour vous sur comment vous vous êtes sentie pendant le questionnaire.** | | |
| O1 | Est-ce que les questions auxquelles vous avez déjà répondu étaient… | 1=Très facile à comprendre<br>2=Assez Facile à comprendre<br>3=Difficile à comprendre<br>4=Tres difficile à comprendre<br>888=Ne sait pas<br>999=Pas de réponse | |
| O2 | En général, avec quel niveau d'honnêteté diriez-vous que vous avez répondu à ces questions ? | 1=Pas du tout honnête<br>2=Un peu honnête<br>3=Assez honnête<br>4=Tres honnête<br>5= Complètement honnête<br>888=Ne sait pas<br>999=Pas de réponse | Choisir une |
| O3 | Y'a-t-il d'autres choses que vous voudriez ajouter ou des questions à me poser? | | |
| O6 | Pour finir cette interview, as-tu préféré donner des réponses directement à l'intervieweur ou en utilisant le tablet | 1=Donner des réponses directement à l'enquêtrice<br>2=Utilisant le tablet<br>3=Pas de préférence<br>888=Ne sais pas<br>999=Pas de réponse | |
| | **(FAIRE UN DEBRIEF AVEC LA FILLE : LA REMERCIER POUR SA DISPONIBILITE ET SON HONNETETE POUR REPONDRE AUX QUESTIONS. LUI DIRE QUE VOUS COMPRENEZ QUE LES QUESTIONS ÉTAIENT DIFFICILES ET QU'Y REPONDRE N'ETAIT PAS FACILE. LUI ASSURER QUE SES RÉPONSES SONT CONFIDENTIELLES. L'INFORMER QU'ELLE PEUT CONTACTER L'ÉQUIPE DE RECHERCHE N'IMPORTE QUAND POUR DES QUESTIONS ET DES PRÉOCCUPATIONS.)**<br><br>**LIRE CE QUI SUIT: L'endroit où nous sommes actuellement appartient à l'OCB (NOM DE L'OCB) dans laquelle il y a des femmes qui fournissent le soutien psychosocial. Une des ces femmes est juste a cote maintenant et je peux vous conduire directement à elle si vous voulez ; à part ça, vous pouvez rentrer ici n'importe quand pour parler avec une de ces femmes, ou je peux vous** | | |

| Girl survey (DRC) | | | |
|---|---|---|---|
| **Question #** | **Question** | **Response options** | **Instructions** |
| | **donner son numéro de téléphone.**<br><br>**(DONNER LA LISTE DE CONTACT DES SERVICES. SI ELLE NE VEUT PAS PRENDRE LA LISTE INFORMEZ-LA QU'ELLE PEUT CONTACTER L'EQUIPE DE RECHERCHE N'IMPORTE QUAND POUR L'ASSISTANCE**<br><br>**DEMANDEZ-LUI SI ELLE A D'AUTRES QUESTIONS)** | | |
| **P. Questions pour l'enqueter** | **ENQUETEUR: MERCI DE REPONDRE AUX QUESTIONS SUIVANTES AVANT DE TERMINER L'INTERVIEW** | | |
| P1 | L'interviewée semblait-elle comprendre les questions? | 1= Tout le temps<br>2<br>3= De temps en temps<br>4<br>5= Jamais | Choisir entre 1 et 5 |
| P2 | L'interviewée semblait-elle répondre aux questions de façon aléatoire? | 1= Tout le temps<br>2<br>3= De temps en temps<br>4<br>5= Jamais | Choisir entre 1 et 5 |
| P3 | L'interviewée semblait-elle réfléchir a chaque réponse avant de répondre ? | 1= Tout le temps<br>2<br>3= De temps en temps<br>4<br>5= Jamais | Choisir entre 1 et 5 |
| P4 | Y'avait-il quelqu'un d'autre présent au moment de l'interview ? | 1=Oui<br>2=Non | Si "Oui", procéder à P4b et P4c<br>Si "Non", passer à P5 |
| P4b | Qui était cette personne? | ________ | Ecrire seulement la relation avec l'interviewée |
| P4c | Jusqu'à quel niveau sentez-vous que la présence de cette personne influençait les réponses données par l'interviewée? | 1= Beaucoup<br>2= Un peu<br>3= Très peu<br>4= Pas du tout | |
| P5 | L'interview a-t-elle été interrompue pour une quelconque raison? | 1=Oui<br>2=Non | Si "Oui", procéder à P5b et P5c<br>Si "Non", passer à P6 |
| P5b | Pourquoi l'interview a-t-elle été interrompue? | ______________ | |
| P5c | Selon vous, cela a-t-il affecté l'interview? | 1=Oui<br>2=Non | |
| P6 | Quel était le contexte dans lequel l'interview a eu lieu? | 1= Calme, privé<br>2= Quelque bruit, presque privé<br>3= Trop de bruit, des gens tout autour | |
| P7 | Comment évalueriez-vous la capacité de l'interviewée de comprendre la plupart des questions? | 1= N'a pas beaucoup compris<br>2= A compris un peu<br>3= A compris modérément<br>4= A beaucoup compris<br>5= A compris énormément | |

| **Girl survey (DRC)** | | | |
|---|---|---|---|
| **Question #** | **Question** | **Response options** | **Instructions** |
| P8 | Quelles questions ont semblé plus difficile à comprendre pour l'interviewée ? | | S'il vous plait, lister les questions |
| P9 | En général, comment évaluerez-vous le nivaur d'intérêt que l'interviewée portait à l'interview? | 1= Vraiment haut<br>2= Au dessus de la moyenne<br>3= Moyenne<br>4= En dessous de la moyenne<br>5= Vraiment bas | |
| P10 | Comment avez-vous trouvez l'utilisation de la tablette par la fille? | | |
| P11 | L'interviewée a-t-elle besoin d'une référence à un prestataire de services quelconque? | | Si oui, décrire s'il vous plait. |
| | **FIN DU QUESTIONNAIRE, TRES BON TRAVAIL!** | | |
