# Supplementary material for: Gender-equitable caregiver attitudes and education and safety of adolescent girls in South Kivu, DRC: A secondary analysis from a randomized controlled trial
Source: PLoS Med. 2021 Sep 28;18(9):e1003619. doi: 10.1371/journal.pmed.1003619 (PMC8478225; doi:10.1371/journal.pmed.1003619)
Supplement: S2 Questionnaire — (PDF) [file pmed.1003619.s005.pdf]

Girls' survey- Mashi

DRC questionnaires quantitatifs

Amadoso g'abananyere - MASHI			
**Q#**	**Madoso**	**Mashuzo**	**Instructions**
**A.**	**Questions administratives**		
A1	Izino ly'olugo/Nom du village	_____ _____ _____	
A2	Cishagala/karatye/Quartier	_____ _____ _____	
A3	Murhundu /Sous village	_____ _____ _____	
A4	Ecimanyiso c'omushonderezi/Code chercheur	_____ _____	
A5	Olusiku lwene/lwe anketi/Date	________/________/_________	jj/mm/aaaa
A6	Kasanzi kukurhangira e anketi/Heure du debut	___________: ___________	24 heures
A7	Akasanzi kokumala/kuyusa/Heure de la fin	___________: ___________	24 heures
A8	Echimanyiso ch'Omubusi	________________	
A9	Echimanyiso ch'Omunyere/Mwananyere	_________________	
A10	Emyaka y'Omunyere		
**B.**	**Informations Démographiques/Ebiyerekire akalamo**		

Rhwamacibi lamusa na koko kandi,okubuganana kili aganadoso,nakasimire oku bakengeza ebirhwazindigi ganira ko.Rhwabadosa kushimbana na kalomo kinyu,na k'omulala,n'emasomo(akaba onasome)n'omurhulan'oku mulama n'abandi banyere n'abalume kandi n'ebi muchifinja omu nsiku zaisha.Chiru ankaba nta bunguke kuli mwe mwakano kasanzi ka madoso,murhanahabwe enfranga erhi kandi kantu ,rhwakulesa ebi mwarhubwira omu kuyinjihya omukolo haguma na bananyere omu cihugo cirhu RDC,na cikebirwe oku mwarhimanya n'obushinganyanya omu kusuza.

Murhasibwi okurhuganiza,mwanarhubwira oku murhalonzizi okurhuganiza rhunagaye.Kandi mwanaleka okushuza oku ngasi idosomurhalonzizi nka mwakayunva lirhabasimisizi,na ntabyo byakuhikira,rhwana gendekera n'idoso likulikire

Byoshi mwarhubwira bya kolesibwa oku busagasi bonene,na ntaye(ciru omumulala gwawe,mwira erhi wundi y'omulugo)wayishi manya ebi wadesire.Byoshi warhubwira lyaba ihwe,Kandi rhwakolesa amashini lyo mumanya oku ciru na nani hali amashuzo ginyu ntamanye.

Embere rhurhondere nalonza manye erhi muciyunvire bwinja na kandi mwayunvirhe ebirhwaganira ko.Ka mugwerhe idoso ?Ka muyemire oku nganirira ene ?
Kwinja.Rhwarhondera na amadoso malembu oku bibayerekire.

Girls' survey- Mashi

Amadoso g'abananyere - MASHI			
**Q#**	**Madoso**	**Mashuzo**	**Instructions**
B2	Ka orhasagi ja ciru e masomo?	1= Neci<br>2= Nanga<br>888 = Arhamanyiri<br>999 = Nt'ishuzo	
B3	Isomo lihi lizinda wayusize ? Omu kanga wagalukire ?	_____ _____<br>888 = Arhamanyiri<br>999 = Nt'ishuzo	
B4	Kawa yandisibwe omumasomo muli ogu mwaka gwa (2015-2016)?	1= Neci<br>2= Nanga<br>888 = Arhamanyiri<br>999 = Nt'ishuzo	
B5	Bici bya rhumaga orhaciyandisa e masomo muli ogu mwaka gwa amasomo gwa (2015-2016)?	1=omulala gurhagwerhe birugu (buhashe),<br>2=aheruka,<br>3=mikolo minji minji ya omunyumpa,<br>4=amasomo gali kuli bwenene, ntamasomo gali olunta lwa e mwirhu,<br>5=omulala gurhayemeraga bulya ntabunguke gwabwine,<br>6=harhali (ntebe) nafasi, amasomo gali gayunjwire,<br>7=ali izimi,<br>8=kuyusa amasomo,<br>9=kudubula e masomo,<br>10=ebindi<br>888 = Arhamanyiri<br>999 = Nt'ishuzo	
B6	Omugobe muzinda gw'amasomo ka hali olusiku wagosize ? Akaba neci kanga?	0=0 (busha),<br>1=1 (ciguma),<br>2=2 (bibihi),<br>3=3 (bisharhu),<br>4=4 (bini),<br>5=5 (birhanu)<br>6=6 (ndarhu)<br>888 = Arhamanyiri<br>999 = Nt'ishuzo	

Girls' survey- Mashi

Amadoso g'abananyere - MASHI			
**Q#**	**Madoso**	**Mashuzo**	**Instructions**
B7	Olusiku luzinda wagosagya a masomo, bici byarhumaga ?	1=mulwala ;<br>2=agwarha emikolo minji yaha ka (ya omu nyumpa) ya kujirwa ;<br>3=kugwarha omwana erhi kulanga mwene wirhu mulwala ;<br>4=kugendi jira omukolo lyo mbona olusaranga ;<br>5=kurhama bwenene ;<br>6=milali na abira (ma programme) ;<br>7=kurhalonza okuja emasomo ;<br>8=kuja omu mugongo (kubona abazungu) ;<br>9=ebindi.<br>888 = Arhamanyiri<br>999 = Nt'ishuzo	Akaba bindi oderhe
B8	Kuleka emikolo ya aha ka (ya omunyumpa), ka okola okolire e nsaranga erhi kandi yindi mikolo ya kuhanirwa nsaranga ? yakulyulwa	1= Neci<br>2= Nanga<br>888 = Arhamanyiri<br>999 = Nt'ishuzo	
B9	Yindi mikolo yagurhi ya nsaranga wajijire erhi yindi yakulyulwa muli iyi mwezi ikumi nibirhi ya geraga?	1=ntayo,<br>2=mukolo gwa omu lwitera (butiki),<br>3= mwananyere mulezi wabana omu yindi milala,<br>4=kulera abana oku gundi mulala,<br>5=mukolo g'obuhinzi ,<br>6=bubasi,<br>7=bulenga bwa amaboko,<br>8=kushenya,<br>9=birhimbuzi<br>10= bindi<br>888 = Arhamanyiri<br>999 = Nt'ishuzo	Orhole ngasi byosshi biribyajirika
B10	Kuleka e mikolo ya haka (ya omunyumpa) ka okola okozire omukolo barhakulyulaga ko ? Barhakulipaga ?	1=ntago,<br>2=mukolo gwa lwitera (butiki),<br>3=mwananyere o kola emikolo ya aha ka (omunyumpa) oku yindi milala, 4=bulezi bwa abana ba ogundi mulala,<br>5= Kuhinga/travaux de champ,<br>6= Kuyubaka/construction,<br>7= Bindi/autre<br>888 = Arhamanyiri<br>999 = Nt'ishuzo	Orhole ngasi byosshi biribyajirika

Girls' survey- Mashi

Amadoso g'abananyere - MASHI			
**Q#**	**Madoso**	**Mashuzo**	**Instructions**
B11	Nabuno nkola nakudosa madoso maguma namaguma gayerekire ababusi bawe ba omubiri, ababusi bawe ba okunali bakuburhaga. Mubusi wawe w'omubiri ohi olamire nawe ?	1= larha<br>2= nyama<br>3=Rhwe mbi<br>4=Ntayo<br>888 = Arhamanyiri<br>999 = Nt'ishuzo	
**C.**	**Lugenda borhere na ababusi kandi nabandi bantu bakulu**<br><br>**Oli wajira omukolo mwinja!oku madoso abirhi gakulikire na derha kantu kaguma ,kankasima mumbwire erhi kobinali,kobili hitya erhi birhakuli bwenene.Ntakadali nko orhagwerhi isuzo.Kamuyunvirhe?**		
C1	Haba omuntu mukulu omukalamo kawe okuhanula.<br>kawanaderha oku oko	1= Birhakuli bwenene<br>2= Bikuli hitya<br>3= Kobinali<br>888 = Arhamanyiri<br>999 = Nt'ishuzo	
C2	Hali omuntu mukulu omukalamo kawe orhola nka cirongozi<br><br>Cirongozi, nalonza okuderha omuntu okuhugula, ola uku ha e ntimanya, akuhe na mahano minja ga kurhabala ukaja irenge omukalamo.<br><br>Kawana derha oku ogu mulongo guli…	3= Kobinali<br>2= Bikuli hitya<br>1= Birhakuli bwenene<br>888 = Arhamanyiri<br>999 = Nt'ishuzo	
C[illegible]	Ndi orhola nka cirongozi cawe?	1=nyama,<br>2=larha,<br>3=mugaka,<br>4=shakulu,<br>5=mwali,<br>6=mushinja,<br>7=nyamashenge,<br>8=nyamalume,<br>9=iba,<br>10=mwiralume<br>11=bandi bakulu,<br>12=Mukulu w'ecigamba cha abanyere<br>888 = Arhamanyiri<br>999 = Nt'ishuzo	

Girls' survey- Mashi

Amadoso g'abananyere - MASHI			
**Q#**	**Madoso**	**Mashuzo**	**Instructions**
C4	Lugenda bici muganira ko mweshi na cirongozi cawe ?	1=majambere,<br>2=mazibu gamagala nkana,<br>3=nyigirizo za masomo,<br>4=abirhu aha masomo,<br>5=mazibu ga milala,<br>6=ntambala (kadali) rhwene ababusi birhu erhi abarhurhabala,<br>7=ntambala (kadali) mweshi na bali, mwiralume<br>, erhi bira ba balume,<br>8= ntambala na abira kandi na abalungu,<br>9= ntambala na bali birhu na bashinja bani,<br>10= ntambala e bwa mukolo,<br>11= mihigo yokurhindika,<br>12= mihigo minja ya mikolo,<br>13= mihigo minja mugwerhe mwene omwira wawe mwiralume erhi balo<br>888 = Arhamanyiri<br>999 = Nt'ishuzo	
C5	**Kandi nakukosa gurhi muciyunva omu kuganira emyanzi miguma na miguma ya kalamo mwene ababusi bawe/bimangizi bali mwechi chigabi? Nalonza mumbwirw erhi munachiyunve Bwinja erhi kubi nkamulimwaganira n'ababusi binyu.Ntakadali akaba orhamanyiri erhi akaba orhalonzizi okushusa.koyunvirhe ?**		
C5a	K'Onaciyunve bwaja erhi kubi omu kuderha ebiyerekire amasomo gawe n'ababusi erhi abimangizi bawe ?	1=Bwinja<br>2=Kubi<br>888 = Arhamanyiri<br>999 = Nt'ishuzo	
C5b	NAKushimbana na gurhi wakayishi bano akalamo kinja omu nsiku zayisha.	1=Bwinja<br>2=Kubi<br>888 = Arhamanyiri<br>999 = Nt'ishuzo	
C5c	N'oku biyerekire obuhya n'amango ababusi binyu babarheganyize okuheruka	1=Bwinja<br>2=Kubi<br>888 = Arhamanyiri<br>999 = Nt'ishuzo	
C5d	Omushuko (puberté)/ mpindulo zicibonesa oku mwana amango akola aja kula)	1=Bwinja<br>2=Kubi<br>888 = Arhamanyiri<br>999 = Nt'ishuzo	

Girls' survey- Mashi

Amadoso g'abananyere - MASHI			
**Q#**	**Madoso**	**Mashuzo**	**Instructions**
C5e	Konaciyunve bwinja erhi kubi omukushambala ebiyerekire Ebirumbu by'obuhya /esexe n'ababusi erhi bimangizi bawe	1=Bwinja<br>2=Kubi<br>888 = Arhamanyiri<br>999 = Nt'ishuzo	
C5f	Kushimbana na Gurhi mwakajira lyo muyaka okuheka izimi	1=Bwinja<br>2=Kubi<br>888 = Arhamanyiri<br>999 = Nt'ishuzo	
C5g	Okubiyerekire Olya VIH/ SIDA erhi ezindi ndwala zigwarha omukujira echijiro ch'obuhiya	1=Bwinja<br>2=Kubi<br>888 = Arhamanyiri<br>999 = Nt'ishuzo	
**D.**	**Obulanzi**	**Agamadoso gakulikire ga shimbulire oku rhimanyize enyanya y'obulanzi buawe omu kalamo kawe omu lugo lwawe n'aha ociyunva omu murhula.**	
D1	K'onaciyunve omumurhula ahamwinyu	1=Neci<br>2=Nanga<br>888 = Arhamanyiri<br>999 = Nt'ishuzo	
D2	K'onaciyunve omumurhula ahamasomo ?	1=Neci<br>2=Nanga<br>888 = Arhamanyiri<br>999 = Nt'ishuzo	Akaba badesire nanga oku idoso B2 orhadosagya er'idoso.
D3	K'onaciyunve omumurhula ahamwabo omwira wawe?	1=Neci<br>2=Nanga<br>888 = Arhamanyiri<br>999 = Nt'ishuzo	
D4	Kob'oyunvirhe oherhe omurhula aha m'omulungu	1=Neci<br>2=Nanga<br>888 = Arhamanyiri<br>999 = Nt'ishuzo	
D5	Kahali ahandi hantu omu lugo hakaba ociyunvirhe omu omurhula kuleka amasomo, ahaka ahawaka ciyunva ogandazize omukushambala bwinja nabandi banyere ?	1=Neci<br>2=Nanga<br>888 = Arhamanyiri<br>999 = Nt'ishuzo	
	**Buno buno, nkola nakudosa madoso maguma maguma enyanya z'abira bawe**	**Okasimisibwa, ombwire eri oyemire kobinali, erhi orhayemiri ,arhali kobinali.Ntakadali akaba orhamanyiri erhi orhalonza okushuza. Koyunvirhe**	

Girls' survey- Mashi

Amadoso g'abananyere - MASHI			
**Q#**	**Madoso**	**Mashuzo**	**Instructions**
D6	Mperhe abira nshambala nabo emianzi ya obulagirire bwenene *ko yemire erhi orhayemiri*	1= Nyemire<br>2= Ntayemiri<br>888 = Arhamanyiri<br>999 = Nt'ishuzo	
D7	Mperhe abira ncikubagira bakangwarhirira.	1= Nyemire<br>2= Ntayemiri,<br>888 = Arhamanyiri<br>999 = Nt'ishuzo	
D8	Kojiraga abira babakazi/banyere b'emiaka yawe aharhali omu mulala ?	1= Neci<br>2= Nanga<br>888 = Arhamanyiri<br>999 = Nt'ishuzo	Si Non ou Ne sait pas/refus, sauter A la question D10.Akaba nanga erhi ntamanyiri oje ouku D10
D9	Bira banga babakazi /banyere ojira ?	0 =0<br>1 = 1-3<br>2 = 4 -10 bira<br>3 = Kulusha 10 bira<br>888 = Arhamanyiri<br>999 = Nt'ishuzo	
D10	Kuleka omuntu womumwinyu, kahali owindi womu lugo womu kazi oyujibwira kanji kanji amazibu gawe	1=Neci<br>2=Nanga<br>888 = Arhamanyiri<br>999 = Nt'ishuzo	
**E.**	**Bulonza bwa kuyorha**	**Bunola nkola nakudosa kushimbana n'ebihikira omunyere omu kalamo kage,lwiganyo masomo,buhya**	
E1	Omu isomo lya kanga/Kuhika oku cigabi cihi camasomo omwananyere akayunjuza (akabumba) embere aleke amasomo ?	___________(Cigabi ca amasomo)<br>888 = Arhamanyiri<br>999 = Nt'ishuzo	
E2	OKU MWAKA INGA/Kuhika myaka inga omwananyere akaherukira	____________(mwaka)<br><br>888 = Arhamanyiri<br>999 = Nt'ishuzo	
E3	Okumyaka inga orhimanyizeko omwananyere akaba na akabonjo KABURHANZI (mwana) ?	___________(mwaka)<br><br>888 = Arhamanyiri<br>999 = Nt'ishuzo	
**G**	**Okujigubagira**		

Girls' survey- Mashi

Amadoso g'abananyere - MASHI			
**Q#**	**Madoso**	**Mashuzo**	**Instructions**
Oyegerere /karibu okukolesa amashini/ACASI. Izino lyani niye… .... nkola na kusomera amadoso.Okengere oku wanahamagala owakurhabala ngasi kasanzi wakaba n'amadoso erhi nkahali ebirhagendekiri bwija omukukolesa amashini/Tablette<br><br>Burhangiriza nakusomera bintu ikumi (10) nkaba byankakuyerekera.Bikusimise okumbwira gurhi wakahamiriza kuli ngasi kantu,Ankaba ohemire bwenene,ohemire,orhahemiri erhi orhakayemera ciru ne hitya.Nta bubi akaba orhamanyiri erhi akaba orholonzizi okushuza. Koyunvirhe?			
G1	Na nkaciyunva oku nani nyumanine n'abandi	4= Kuyemera bwenene<br>3=Kuyemera<br>2=Ntakuyemera<br>1=Ciru ntakuyemera<br>888 = Arhamanyiri<br>999 = Nt'ishuzo	
G2	Nyunvirhe okunjira nka ngeso nyinja hofinga.	4= Kuyemera bwenene<br>3=Kuyemera<br>2=Ntakuyemera<br>1=Ciru ntakuyemera<br>888 = Arhamanyiri<br>999 = Nt'ishuzo	
G3	Nankaciyunva oku mpimirwe na ntabyo nakahasha	4= Kuyemera bwenene<br>3=Kuyemera<br>2=Ntakuyemera<br>1=Ciru ntakuyemera<br>888 = Arhamanyiri<br>999 = Nt'ishuzo	
G4	Nanajira ebintu nkokuhofi abandi bantu bakabijira	4= Kuyemera bwenene<br>3=Kuyemera<br>2=Ntakuyemera<br>1=Ciru ntakuyemera<br>888 = Arhamanyiri<br>999 = Nt'ishuzo	
G5	Nyunvurhe okugwerhe enkwa yokusimiswa oku nandi	4= Kuyemera bwenene<br>3=Kuyemera<br>2=Ntakuyemera<br>1=Ciru ntakuyemera<br>888 = Arhamanyiri<br>999 = Nt'ishuzo	

Girls' survey- Mashi

Amadoso g'abananyere - MASHI			
**Q#**	**Madoso**	**Mashuzo**	**Instructions**
G6	Ncigwerhe ko oborhere bwinja	4= Kuyemera bwenene<br>3=Kuyemera<br>2=Ntakuyemera<br>1=Ciru ntakuyemera<br>888 = Arhamanyiri<br>999 = Nt'ishuzo	
G7	Nchisimire nkoku nandi	4= Kuyemera bwenene<br>3=Kuyemera<br>2=Ntakuyemera<br>1=Ciru ntakuyemera<br>888 = Arhamanyiri<br>999 = Nt'ishuzo	
G8	Nankaciyunva oku chikenzire,Ngwerhe bukenge buji kuli niene	4= Kuyemera bwenene<br>3=Kuyemera<br>2=Ntakuyemera<br>1=Ciru ntakuyemera<br>888 = Arhamanyiri<br>999 = Nt'ishuzo	
G9	Nankaciyunva oku nani ndi muntu wa bulagirire	4= Kuyemera bwenene<br>3=Kuyemera<br>2=Ntakuyemera<br>1=Ciru ntakuyemera<br>888 = Arhamanyiri<br>999 = Nt'ishuzo	
G10	Nchiyunvirhe oku nandi muntu okwanine/olinganine	4= Kuyemera bwenene<br>3=Kuyemera<br>2=Ntakuyemera<br>1=Ciru ntakuyemera<br>888 = Arhamanyiri<br>999 = Nt'ishuzo	
**H.**	**Histoire sexuelle :**		
Omusagasi : Nkolaga nakudosa madoso maguma na maguma gayerekire oku olama n'abalume. Okengere harhali amashuzo minja erhi mabi n'okundi obe n'obulangalire oku ntaye wa kushobeka. Oshubikengera oku Amashuzo gawe garhayandikwe k'izino lyawe garhanayerekanwe oku bandi bantu kulusha abalibajira obusagasi<br>Na bulya oli wakolesa aga mashini,n'oyu musagasi arhananye bici washuzize.Kandi wanaderha oku orhamanyiri erhi oku orhalonzizi kushuza na ntabyo byakuhikira			
I	**Kanji kanji omulume agaya erhi abe burhe n'ebi mukage ali ajira. Oku bwawe, k'omulume akwanine okushurha mukage mwago mango ? Nakusomera biguma biguma nawe ombwire erhi kushimbana n'oko oyunvirhe oku omulnme akwanine okushurha mukage.**			

Girls' survey- Mashi

Amadoso g'abananyere - MASHI			
**Q#**	**Madoso**	**Mashuzo**	**Instructions**
I1	Kakuyemerirwe omulume ashurhe mukage Erhi akarhenga ahaka buzira okumubwira ?	1= Neci<br>2= Nanga<br>888 = Arhamanyiri<br>999 = Nt'ishuzo	
I2	Kakuyemerirwe omulume ashurhe mukage Erhi akaba arhashibirira bwinja abana bage?	1= Neci<br>2= Nanga<br>888 = Arhamanyiri<br>999 = Nt'ishuzo	
I3	Kakuyemerirwe omulume ashurhe mukage Nka akalongola n'iba?	1= Neci<br>2= Nanga<br>888 = Arhamanyiri<br>999 = Nt'ishuzo	
I4	Kakuyemerirwe omulume ashurhe mukage Nka akalahirira iba icijiro ch'obuhya?	1= Neci<br>2= Nanga<br>888 = Arhamanyiri<br>999 = Nt'ishuzo	
I5	Kakuyemerirwe omulume ashurhe mukage Nka akasiriza ebiryo ?	1= Neci<br>2= Nanga<br>888 = Arhamanyiri<br>999 = Nt'ishuzo	
**J**	**Chemisi comubiri okubulisibwa omurhula n'akanwa kabi**		
	Enumérateur : Amadoso gakulikire gashimbine n'ebintu byankabahikira.Rhuyunvirhe oku madoso maguma na maguma gayerekire omuntu yenene gananarhuma achiyunva kubi,Cikoki rhucikebirwe oku mwarhubwira byoshi mumanyire lyo rhuhashi yunva amalagirier gabandi bananyere bali akinyu/nkamwe.Omanye oku izimo lyawe lirhayandikwe hantu,na bulya oili wakolesa amashini/Tablette Ntaye yeshi yeshi wamanya amashuzo gawe.Onakengere oku ngasi kantu koshi bakola bakujirire lirhali igosa lyawe. Ociyunve bwinja okushuza nkoku bishinganine buzira kufulika akantu,Orhanasezibwi okushuza oku idoso nka orhaciyunvirhi bwinja."		
J1	Muli eyi mwezi 12 yageraga Kahali okola okushurhire, akuha oluhi, erhi alonza okukuyagaza ?	1= Neci<br>2= Nanga<br>888 = Arhamanyiri<br>999 = Nt'ishuzo	Si non ou ne sait pas passez a J3
J2	Indi wakuyagazize ntyo/oko?	1= mwiralume / mshangizi/ ibanie wene<br>2=Mubusi/mulanzi<br>3= wundi w'omumulala<br>4= Mwira erhi mulungu<br>5= Yomucigamba cha baheka embunduzi<br>6=Mukulu mulebe/AUTORITE<br>7= Bandi/AUTRES<br>999 = Nt'ishuzo	(cochez toute réponse qui convient) OYANDIKE NGASI EBISHINGANINE

Girls' survey- Mashi

Amadoso g'abananyere - MASHI			
**Q#**	**Madoso**	**Mashuzo**	**Instructions**
J3	Omu miezi 12 egezire/ehwire /yinjo, kahali owakufokeraga/owakukankamiraga/ wakuyamiraga, alonza okukukolera amaligo ?	1= Neci<br>2= Nanga<br>888 = Arhamanyiri<br>999 = Nt'ishuzo	SI NON OU NE SAIS PASSER A J5. Akaba naga erhi ntamanyiri oje oku J5
J4	Indi wakukankamiraga/ wakuyamire nizu linene erhi alonza okukoler'amaligo ?	1= mwiralume / mshangizi/ ibanie wene<br>2=Mubusi/mulanzi<br>3= wundi w'omumulala<br>4= Mwira erhi mulungu<br>5= Yomucigamba cha baheka embunduzi<br>6=Mukulu mulebe/AUTORITE<br>7= Bandi/AUTRES<br>999 = Ntaishuzo	(cochez toute réponse qui convient) OYANDIKE NGASI EBISHINGANINE
J5	Mweyi mwezi 12 egezire, kanga omuntu mulebe akujacire, akubwira ebibi, erhi akuheherera ?	1= Neci<br>2= Nanga<br>888 = Arhamanyiri<br>999 = Nt'ishuzo	SI NON OU NE SAIS PAS PASSEZ A J7
J6	Indi wakujacire, akubwira ebibi, erhi akuheherera?	1= mwiralume / mshangizi/ ibanie wene<br>2=Mubusi/mulanzi<br>3= wundi w'omumulala<br>4= Mwira erhi mulungu<br>5= Yomucigamba cha baheka embunduzi<br>6=Mukulu mulebe/AUTORITE<br>7= Bandi/AUTRES<br>999 = Nt'ishuzo	(cochez toute réponse ce qui convient) OYANDIKE NGASI EBISHINGANINE
J7	Mweyi myezi 12 ezindigera, ka wayunvirhe nko muntu walikwanine okukushibirira, ali kulekerire ?	1= Neci<br>2= Nanga<br>888 = Arhamanyiri<br>999 = Nt'ishuzo	Si NON OU NE SAIS PAS PASSER ALA SECCTION K. Akaba naga erhi ntamanyiri oje oku madoso ga K
J8	Mweyimwezi 12 egezire, kanga wayunvirhe Ntaye okushibirire ?	1= Kanji<br>2= ligumaliguma<br>3= Ntaliguma<br>888 = Arhamanyiri<br>999 = Nt'ishuzo	
K.	**Violence Sexuelle /OKUGWARHWA CIRIMISI**		

Girls' survey- Mashi

Amadoso g'abananyere - MASHI			
**Q#**	**Madoso**	**Mashuzo**	**Instructions**
	**Koko okushuza kwaga madoso oku bintu walamire.Manyire oku okushaza oku madoso ga ntyo kurhalikulembu.Ebikulikire nabyo bigwerhe amadoso mazibu,cikone rhucikebirwe oku omu kukolesa amashini/Tablette wanachiyunva bwinja omu kurhubwira ibi okola ogezire mo.**<br><br>**Byanahikira Abanyere n'abakazi oku buganana mubiri oku gundi buzira bulonza bwabo n'abantu bamanyire,nkakula mwira w'okumurhima,muntu w'omumulala erhi mwira,kandi erhi oyu barhamanyiri.Aga madoso gayerekire omuntu yenene gananarhuma omuntu ajiyunva kubi omu kushuza,cikone warhurhabala okumunya gurhi abantu bayunva omukubuganana mubiri oku gundi buzirha buloza erhi luhusa lwabo.Amashuzo gawe liri ihwe kandi wanarhalukira ngasi idoso orhalonzizi okushuza ko.Kandi okengere oku orhasambisibwe,n'oku harhali ishuzo linja erhi libi**<br><br>**Okengere oku ngasi kantu wajirirwe n'omuntu mukulu buzira luhusa lwawe,lirhali igosa lyawe**		
K1	Kahali omuntu akola okuhumire ko nkakukulonza ko ejijiro c'obuhya ?	1= Neci<br>2= Nanga<br>888 = Arhamanyiri<br>999 = Nt'ishuzo	Si non ou ne sais pas passer a K4
K2	Ndi oyo muntu wajiraga ntyo ?	1= mwiralume / mshangizi/ ibanie wene<br>2=Mubusi/mulanzi<br>3= wundi w'omumulala<br>4= Mwira erhi mulungu<br>5= Yomucigamba cha baheka embunduzi<br>6=Mukulu mulebe/AUTORITE<br>7= Bandi/AUTRES<br>999 = Nt'ishuzo	(cochez toute réponse ce qui convient)<br>OYANDIKE NGASI EBISHINGANINE
K3a	Muli eyi mwezi 12 egezire hali omuntu wakuhumire ko nkowakulonza ko ejijiro c'obuhya buzira bulonza/luhusa bwawe.	1= Neci<br>2=Nanga<br>888= Ntamanyiri<br>999 = Nt'ishuzo	Si Non passer a K4<br>AKABA NANGA OGENDEKERE NA K4
K3	Kanga muli eyi mwezi 12 egezire omuntu akola okuhumire ko nkowakulonza ko ejijiro c'obuhya buzira bulonza bwawe	……………………………………<br><br>888 = Arhamanyiri<br>999 = Nt'ishuzo	

Girls' survey- Mashi

Amadoso g'abananyere - MASHI			
**Q#**	**Madoso**	**Mashuzo**	**Instructions**
K4	Kahali omuntu wakolesize erhi alonzize okukolesa emisi erhi obuhashe bwage kushimbana oku ali mukulu erhi ayimangirwe lyo mugwishira mwenaye ?<br><br>Lwiganyo aderhe mpu akuha ehalama mbi emasomo, erhi Akujira kubi?	1=Neci<br>2= nanga<br>888 = Arhamanyiri<br>999 = Nt'ishuzo	Si non ou ne sais pas paser a K7
K5	Ndi oyo muntu wajiraga ntyo ?	1= mwiralume<br>/ mshangizi/ ibanie wene<br>2=Mubusi/mulanzi<br>3= wundi w'omumulala<br>4= Mwira erhi mulungu<br>5= Yomucigamba cha baheka embunduzi<br>6=Mukulu mulebe/AUTORITE<br>7= Bandi/AUTRES<br>999 = Nt'ishuzo	(cochez toute réponse qui convient)<br>OYANDIKE NGASI EBISHINGANINE
K6a	Omu miezi ikumi n'ibiri mizinda /yazindigi gera kawahushire n'omuntu bulya akusezize n'okukolesa obuhashe bwage, mpu lyo muhusha mwenaye	1=Neci<br>2= nanga<br>888 = Arhamanyiri<br>999 = Nt'ishuzo	If "No or "Don't know" skip to question K7
K6	Omu miezi ikumi n'ibiri mizinda /yazindigi gera KANGA wahushire n'omuntu bulya akusezize n'okukolesa obuhashe bwage, mpu lyo muhusha mwenaye	_____ _____<br><br>888 = Arhamanyiri<br>999 = Nt'ishuzo	
K7	K'okola ojijir'obugonyi n'omulume mulama haguma lyo abona enfaranga,erhi bindi n' ebyokulya ?	1= Neci<br>2 =Nanga<br>888 = Arhamanyiri<br>999 = Nt'ishuzo	Si non, passer a K9<br>Akaba nanga oje oku K9
K8	K'ebi Byakuhikire/byabire muli eyi miezi ikumi n'ibiri mizinda ?	1= Neci<br>2 =Nanga<br>888 = Arhamanyiri<br>999 = Nt'ishuzo	
K9	Kahali omuntu okol'obahir'enfaranga, ebiryo, amatabishi/oluhembo erhi kandi kantu lyo mujira obugonyi mweshi ?	1= Neci<br>2 =Nanga<br>888 = Arhamanyiri<br>999 = Nt'ishuzo	Si non ou ne sais pas passer a K11. Akaba nanga erhi ntamanyiri oje oku K11

Girls' survey- Mashi

Amadoso g'abananyere - MASHI			
**Q#**	**Madoso**	**Mashuzo**	**Instructions**
K10	Kebi byabire muli eyi emiezi ikumi n'ibiri mizinda/YAZINDIGI GERA ?	1= Neci<br>2 =Nanga<br>888 = Arhamanyiri<br>999 = Nt'ishuzo	
	Koko ombwirwe erhi oyemire nici orhayemiri, kuli ibinkola na kudosa buno Kandi wanaderha oku orhamanyiri erhi oku orhalonzizi kushuza na ntabyo byakuhikira		
K11	Omulala gwani gwani gwanapana nkanasezibwa okujira obugonyi ntanalonzizi.	1=Nyemire<br>2 =Ntayemiri<br>888 = Arhamanyiri<br>999 = Nt'ishuzo	
K12	Omulala gwani,Abashamuka/olugo bana nseza mpu mperuke n'omulume erhi akanseza omu kujira obugonyi naye…	1=Nyemire<br>2 =Ntayemiri<br>888 = Arhamanyiri<br>999 = Nt'ishuzo	
K13	Ngwerhe omuntu omulugo oyu nchikubagira bwenene nakabwira erhi nakajir'obugonyi bw'okusezibwa.	1=Nyemire<br>2 =Ntayemiri<br>888 = Arhamanyiri<br>999 = Nt'ishuzo	
**L**	**Mikolo/ABARHABAZI**		
	**Koko okukushuza kuli aga madoso mazibu,okuzire akasi kija bwenene.Nkumanyisize oku harhacibe amadoso gamene ago gokushimbina n'ibi omuntu alamire,erhi ebyahikire omuntu.**<br>**Bunola nkola nadosa madoso maguma maguma enyanya lyaha mwakamanya abanyere bali nka mwe bakarhabalwa nka bagwerhe mazibu malebe**		
L1	Amongo muntu mulebe akagwarha erhi akolonza okugwarha omunyere ciri buzira bulonza bo munyere, Ka mumanyire ahantu omuntu akalibirhira /ankaja oku rhabalwa?	1=Neci<br>2=Nanga<br>888 = Arhamanyiri<br>999 = Nt'ishuzo	
L2	Kamumanyire ahantu omunyere akalibirhira amongo hali omuntu wamushusire?	1=Neci<br>2=Nanga<br>888 = Arhamanyiri<br>999 = Nt'ishuzo	
**M.**	**Obulangalire n'obwererekezi bw'irhindo/Ensiku zayisha**		
	**Bunola nkola nabasomera ebindi kushimbana n'oku mugerereza kuli mwene.Nalonza ombwire kananga ogerereza ntyo kuderha okuntakasanzi,erhi orhagerereza ntyo,erhi kasanzi kasungunu,erhi kasanzi kaguma kaguma,Kasanzi kanji,kandi erhi kasanzi kanji bwenene**<br><br>**Ngasi mango okakengera oku harhali ishuzo linja erhi libi,kandi wanaderha oku orhamanyiri erhi oleke okushuza oku idoso orhalonzizi**		

Girls' survey- Mashi

Amadoso g'abananyere - MASHI			
**Q#**	**Madoso**	**Mashuzo**	**Instructions**
M1	Nankagerereza okunzibuhirhe bwenene. /Oku ebintu byosho bigendekire byinja kuli niye. Kanga ogerereza nyo ?	1= Ntakasanzi<br>2= Kasanzi kasungunu<br>3= Kasanzi Kaguma kaguma<br>4= Kasanzi Kanji<br>5= Kasanzi kanji bwenene<br>888 = Arhamanyiri<br>999 = Nt'ishuzo	
M2	Nakagerereza njira nyinji y'okubona bintu binji byobulagirire kuli niye omu kalamo kani. Kanga ogerereza nyo ?	1= Ntakasanzi<br>2= Kasanzi kasungunu<br>3= Kasanzi Kaguma kaguma<br>4= Kasanzi Kanji<br>5= Kasanzi kanji bwenene<br>888 = Arhamanyiri<br>999 = Nt'ishuzo	
M3	Ngwerhe obulyo bokuhasha akabandi banyere b'emiaka yani. Kanga ogerereza nyo ?	1= Ntakasanzi<br>2= Kasanzi kasungunu<br>3= Kasanzi Kaguma kaguma<br>4= Kasanzi Kanji<br>5= Kasanzi kanji bwenene<br>888 = Arhamanyiri<br>999 = Nt'ishuzo	
M4	Amango ngwerhrwe n'amabizibu, nana bona oku naka cihangana kuligo lyompata ishuzo. Kanga ogerereza nyo ?	1= Ntakasanzi<br>2= Kasanzi kasungunu<br>3= Kasanzi Kaguma kaguma<br>4= Kasanzi Kanji<br>5= Kasanzi kanji bwenene<br>888 = Arhamanyiri<br>999 = Nt'ishuzo	
M5	Ngererize oku ebi najiraga mira byanacintabala omu nsiku zaisha Kanga ogerereza nyo ?	1= Ntakasanzi<br>2= Kasanzi kasungunu<br>3= Kasanzi Kaguma kaguma<br>4= Kasanzi Kanji<br>5= Kasanzi kanji bwenene<br>888 = Arhamanyiri<br>999 = Nt'ishuzo	
M6	Cirhw'ankaba abandi bamayabirwa, manyirire oku nanaba n'enjira y'okulonza ishuzo oku mazibu. Kanga ogerereza nyo ?	1= Ntakasanzi<br>2= Kasanzi kasungunu<br>3= Kasanzi Kaguma kaguma<br>4= Kasanzi Kanji<br>5= Kasanzi kanji bwenene<br>888 = Arhamanyiri<br>999 = Nt'ishuzo	

Girls' survey- Mashi

Amadoso g'abananyere - MASHI			
**Q#**	**Madoso**	**Mashuzo**	**Instructions**
N Question de fin	Koko okubona washuzize okumadoso.Manyire oku hali maguma na maguma gali mazibu.Oshibi kengera oku ntaye muno lugo loshi wamanya ebi washuzire.Rhwamayusa okukola na mashini/Tablette,Bikusimise ohamadale omusagasi n'okumuhereza ago mashini.<br>(UNE FOIS L'ENFANT A TERMINE, REPRENEZ LA TABLETTE ACASI. VERIFIEZ SI TOUTES LES CHOSES SONT CORRECTES ET ALORS LIT LA DÉCLARATION EN DESSOUS:)<br>Koko okubona washuzize oku madoso girhu. Wakozire akasi kinja omu kukolesa amashini n'okushuza oku madoso mazibu.<br>Nkolaga nakudosa amadoso malembu kashimbana n'ebi osima okujira.		
N1	Mukolo guhi osima okujira omulugo ?	……………………………..	
**O**	**Amadoso ga buzinda,Question de conclusion**		
	**Rhukola rhwayusa.**<br>**Embere rhuyuse enshambalo zirhu, ngwerhe madoso maguma na maguma nakudosa kushimbana n'oku wakagi ciyunva omu kushuza oku madoso.**		
O1	Gurhi wayunvirhe aga madoso washuzagya ko?	1=malembu bwenene okuyunva<br>2=malembu okuyunva<br>3=mazibu okuyunva<br>4= mazibu bwenene kuyunva<br>888 = Arhamanyiri<br>999 = Nt'ishuzo	
O2	Kulusha byoshi, okuli kwawe gurhi washuzize n'obushinganyanya oku madoso ?	1=arhali okuli bwenene<br>2= arhali okuli<br>3= okuli hitya<br>4= neci okuli<br>5= okuli loshiloshi<br>888 = Arhamanyiri<br>999 = Nt'ishuzo	
O3	Kahali ebindi bintu wakalonzize okuyushula erhi madoso ga ku ndosa ?		
O6	Omukumala eyishambalo y'amadoso, kawasimisibwe n'okuhana amashuzo dubaduba oku musagasi nisi erhi omukukolesa etablet.	1= Okuha omusagasi amashuzo honaho.<br>2= Omukukolesa etablet<br>3= Ntalyo lyokusimisibwa<br>888= Arhamanyiri<br>999= Nt'ishuzo	

Girls' survey- Mashi

Amadoso g'abananyere - MASHI			
**Q#**	**Madoso**	**Mashuzo**	**Instructions**
	**FAIRE UN DEBRIEF AVEC LA FILLE : LA REMERCIER POUR SA DISPONIBILITE ET SON HONNETETE POUR REPONDRE AUX QUESTIONS. LUI DIRE QUE VOUS COMPRENEZ QUE LES QUESTIONS ÉTAIENT DIFFICILES ET QU'Y REPONDRE N'ETAIT PAS FACILE. LUI ASSURER QUE SES RÉPONSES SONT CONFIDENTIELLES. L'INFORMER QU'ELLE PEUT CONTACTER L'ÉQUIPE DE RECHERCHE N'IMPORTE QUAND POUR DES QUESTIONS ET DES PRÉOCCUPATIONS.**<br><br>**LIRE CE QUI SUIT: Hano rhuli hali aha cigamba cha bene OCB (IZINO LYE OCB) Muba abakazi bakarhabala abantu okuntimanya/Kimafikiri.Muguma muli boa li hano nankolonzise nana kuhisa ahali,kandi ntamango orhakagaluka hano nkolonzize oku ganira abo bakazi/ba maman,erhi nkolonzize nanakuha enomero yabo ye telephone**<br><br>**(DONNER LA LISTE DE CONTACT DES SERVICES. SI ELLE NE VEUT PAS PRENDRE LA LISTE INFORMEZ-LA QU'ELLE PEUT CONTACTER L'EQUIPE DE RECHERCHE N'IMPORTE QUAND POUR L'ASSISTANCE<br>DEMANDEZ-LUI SI ELLE A D'AUTRES QUESTIONS)**		
**P**	**Questions pour l'enqueteur : Merci de Repondre aux questions suivantes avant de cloturer l'interview**		
P1	L'interviewée semblait-elle comprendre les questions?	1= Tout le temps<br>2<br>3= De temps en temps<br>4<br>5= Jamais	Choisir entre 1 et 5
P2	L'interviewée semblait-elle répondre aux questions de façon aléatoire?	1= Tout le temps<br>2<br>3= De temps en temps<br>4<br>5= Jamais	Choisir entre 1 et 5
P3	L'interviewée semblait-elle réfléchir a chaque réponse avant de répondre ?	1= Tout le temps<br>2<br>3= De temps en temps<br>4<br>5= Jamais	Choisir entre 1 et 5
P4	Y'avait-il quelqu'un d'autre présent au moment de l'interview ?	1=Oui<br>2=Non	Si "Oui", procéder à P4b et P4c<br>Si "Non", passer à P5
P4b	Qui était cette personne?	_________	Ecrire seulement la relation avec l'interviewée
P4c	Jusqu'à quel niveau sentez-vous que la présence de cette personne influençait les réponses données par l'interviewée?	1= Beaucoup<br>2= Un peu<br>3= Très peu<br>4= Pas du tout	
P5	L'interview a-t-elle été interrompue pour une quelconque raison?	1=Oui<br>2=Non	Si "Oui", procéder à P5b et P5c<br>Si "Non", passer à P6
P5b	Pourquoi l'interview a-t-elle été interrompue?	_______________	

Girls' survey- Mashi

Amadoso g'abananyere - MASHI			
**Q#**	**Madoso**	**Mashuzo**	**Instructions**
P5c	Selon vous, cela a-t-il affecté l'interview?	1=Oui<br>2=Non	
P6	Quel était le contexte dans lequel l'interview a eu lieu?	1= Calme, privé<br>2= Quelque bruit, presque privé<br>3= Trop de bruit, des gens tout autour	
P7	Comment évalueriez-vous la capacité de l'interviewée de comprendre la plupart des questions?	1= N'a pas beaucoup compris<br>2= A compris un peu<br>3= A compris modérément<br>4= A beaucoup compris<br>5= A compris énormément	
P8	Quelles questions ont semblé plus difficile à comprendre pour l'interviewée ?		S'il vous plait, lister les questions
P9	En général, comment évaluerez-vous le nivaur d'intérêt que l'interviewée portait à l'interview?	1= Vraiment haut<br>2= Au dessus de la moyenne<br>3= Moyenne<br>4= En dessous de la moyenne<br>5= Vraiment bas	
P10	Comment avez-vous trouvez l'utilisation de la tablette par la fille?		
P11	L'interviewée a-t-elle besoin d'une référence à un prestataire de services quelconque?		Si oui, décrire s'il vous plait.
	**FIN DU QUESTIONNAIRE, TRES BON TRAVAIL!**		
