# Supplementary material for: Gender-equitable caregiver attitudes and education and safety of adolescent girls in South Kivu, DRC: A secondary analysis from a randomized controlled trial
Source: PLoS Med. 2021 Sep 28;18(9):e1003619. doi: 10.1371/journal.pmed.1003619 (PMC8478225; doi:10.1371/journal.pmed.1003619)
Supplement: S3 Questionnaire — (PDF) [file pmed.1003619.s006.pdf]

Horoza ya maulizo kwa wabinti - SWAHILI			
**Q#**	**Ulizo**	**Jibu**	**Kanuni za kufuata**
**A**	**Ulizo za usimamizi**		
	Jina la kijiji / mahali	_____ ______ ______	
A2	Kata	_____ ______ ______	
A3	Kijiji kidogo cha kata	_____ ______ ______	
A4	Kitambulisho cha mutafiti		
A5	Tariki ya utafuti	____/____/______	jj/mm/aaaa
A6	Saa ya mwanzo ya utafiti	____ _____ _____	Saa 24
A7	Saa ya mwisho ya utafiti	____ _____ _____	Saa 24
A8	Kitambulisho cha muzazi		
A9	Kitambulisho cha binti		
A10	Myaka ya binti		
**B. Habari za idadi ya watu**	**Jambo tena, na asante kwa kukutana nasi kwa mazungumuzo haya. Ningependa kukumbusha kuhusu yale tuliyo ongeya siku ya mwisho ambayo tulizungumuza. Tutaku uliza ma ulizo kuhusu maisha yako na yale unayo yapitia, pamoja na hayo, jamaa yako, masomo yako (kama unaendaka ku masomo), usalama wako, uhusiyano wako na wengine wabinti na wavulana, na matumaini yako kuhusu maisha ya kesho. Hata kama hauta pata kitu binafsi kwa kushiriki kwa mazungumuzo haya, hauta pata pesa ama zingine faida za vitu, tunakusanya taarifa hizi kwa kuboresha mipangilio kwa faida ya wabinti katika Jamuhuri ya Kidemokrasia ya Congo (RDC), na tunatumaini ya kama utakuwa ukifikiri na kupana majibu ya uaminifu.**<br><br>**Haushurtishwe kwa kutuongelesha siku ya leo. Unaweza sema kama haupendi kutuongelesha leo na hatuta kwazika. Unaweza piya kataa kujibu kwa kila ulizo ambayo haupendi jibu kama unajisikiya vibaya ao hauko huru na hakuna kitu mbaya ambayo itakufikiya; tutapita tu kwa ulizo lifwatalo.**<br><br>**Habari ambayo utatupatiya itatumikishwa tu kwa ajili ya ukomo wa utafiti, na akuna mtu (mukiwamo jamaa lako, warafiki zako, ama watu wengine ndani ya jamii) atakayejuwa yale uliyo yasema. Yale yote unayo tuambiya yatawekwa kwa siri, na tutatumikisha ordinateur kwa kuonyesha ya kama hata mimi, sitajuwa majibu zako funali fulani**<br><br>**Mbele tuanze, ningependa nijuwe kama unajisikiya huru na kama unaelewa yote kuhusu yale tuliyo ongeya. Je, una maulizo? Unakubali kuzungumuza nami kwa siku ya leo?**<br>**Vizuri. Tuna anza na maulizo nyepesi na yanayo kuelekeya**		
B2	Ulishaka wayi kwenda kwenyi shule?	1 = Ndiyo,<br>2 = Apana<br>888 = hayuwe<br>999 = Akuna jibu	Kama apana, pita kwenyi ulizo B8
B3	Ni ngazi gani ya juu uliyo ifikiya kwenyi shule ?	_____ _____<br>888 = hayuwe<br>999 = Pas de réponse	
B4	Umeandikwa kwenyi shule mwaka wa mwisho wa masomo (2015-2016)?	1 = Ndiyo, 2 = Apana<br>888 = hayuwe<br>999 = Akuna jibu	Kama apana, uliza B5<br>Kama ndiyo pita kwenyi B6

Horoza ya maulizo kwa wabinti - SWAHILI			
**Q#**	**Ulizo**	**Jibu**	**Kanuni za kufuata**
B5	Ni sababu gani ya muhimu ambayo ilituma haunge wezeshwa kuandikwa kwenyi shule wakati wa myaka karibuni ya masomo (2015-2016)?	1 = jamaa haikukuwa na uwezo<br>2 = akaolewa<br>3 = majukumu mengi nyumbani<br>4 = shule lilikuwa mbali zaidi/ hapakukuwa shule kandokando<br>5 = jamaa haikukubali/ haikuona faida<br>6 = hapakukuwa nafasi yenyi kuwa huru kwenye shule (shule lilikuwa limejaa)<br>7 = alikuwa muja muzito<br>8 = kumaliza shule<br>9 = kushindwa kwenyi shule<br>10 = ingine<br>888 = hayuwe<br>999 = akuna jibu	Pita kwenye ulizo B8
B6	Wakati wa juma la mwisho la shule, uliwayi kosa masiku? Kama ndiyo, siku ngapi?	0 = 0,<br>1 = 1,<br>2 = 2,<br>3 = 3,<br>4 = 4,<br>5 = 5,<br>6 = 6,<br>888 = hayuwe<br>999 = akuna jibu	
B7	Mara ya mwisho ambayo haukufika kwenye shule, sababu gani haukuenda?	1 = mgonjwa,<br>2 = ningepashwa fanya kazi za nyumbani,<br>3 = ningepashwa jiusisha na mtoto ao mgonjwa,<br>4 = ningepashwa kuenda kutumika kusudi nipate maisha,<br>5 = nilikuwa nimechoka sana,<br>6 = nilikuwa na mipango pamoja na marafiki,<br>7 = sikutaka kuenda huko,<br>8 = siku za damu za kawaida kwa mwanamke,<br>9 = ingine<br>888 = hayuwe<br>999 = akuna jibu	Kama ni ingine, tambulisha
B8	Zaidi ya kazi zako za nyumbani za kurudiliya, umewai kutumika kwa kupata pesa ao aina nyingine ya malipo?	1 = Ndiyo<br>2 = Apana<br>888 = hayuwe<br>999 = Akuna jibu	Kama apana ao hajuwi, pita kwenyi B12
B9	Ni kazi ya namna gani ume fanya mu myezi 12 zilizo pita kwa kupata pesa ao malipo ingine?	1 = akuna,<br>2 = kazi ndani ya duka/zembule,<br>3 = kazi za nyumbani kwa jamaa ingine,<br>4 = kuji husisha na mtoto wa jamaa ingine,<br>5 = kazi za shamba,<br>6 = ujenzi,<br>7 = kutengeza vyombo,<br>8 = kukusanya kuni za kuwasha moto,<br>9 = uchuruzi ndogo,<br>10 = ingine<br>888 = hayuwe<br>999 = akuna jibu	Chaguwa yote inayo tendeka

Horoza ya maulizo kwa wabinti - SWAHILI			
**Q#**	**Ulizo**	**Jibu**	**Kanuni za kufuata**
B10	Kutosha kazi zako za nyumbani za kawaida, umefanya kazi ingine ndani ya myezi 12 zilizo pita ambayo hawakukulipa?	1 = akuna,<br>2 = kazi ndani ya duka/zembule,<br>3 = kazi za nyumbani kwa jamaa ingine,<br>4 = kuji husisha na mtoto wa jamaa ingine,<br>5 = kazi za shamba,<br>6 = ujenzi,<br>7 = ingine<br>888 = hayuwe<br>999 = akuna jibu	Chaguwa yote inayo tendeka
B11	Sasa, nilitaka ni kuulize maswali fulani kuhusu wazazi wako wa kimwili, nikusema wazazi kawaida walio kuzaa.<br>Ni yupi kati wazazi wako wakukuzaa ana ishi nawe ?	1=baba<br>2=mama<br>3=sisi wa wili<br>4=Akuna mutu<br>888 = hayuwe<br>999=Hakuna jibu	
**C. Uhusiano na wazazi pia na wakubwa wengine.**	**Umefanya kazi nzuri sana!**<br><br>**Kwa maulizo mbili zinazo fwata, nitasema kitu moja na ningependa uniambiye kama ni "hakika kweli", "kweli kidogo" ama "siyo kweli sana". Ni vizuri kama haujuwi ama kama hauna jibu. Je, nasikiya?**		
C1	Kuna mutu mzima ndani ya maisha yako anakupa mashauri.<br><br>***Unaweza sema kama hii msemwa ni …***	1 = siyo kweli kwa sana<br>2 = kweli kidogo,<br>3 = hakika kweli,<br>888 = hayuwe<br>999 = akuna jibu	
C2	Kuna mutu muzima mumoja ndani ya maisha yako ambaye unaye mukamata kama muongozi ao mushauri<br>Kwa muongozi / mushauri nataka kusema mutu anaye kuongoza, ku sapoti/kusaidiya kimafikiri, na anakupa mashauri mazuri yanayo kusaidiya kupata ushindi/kufaulu ndani ya maisha.<br><br>***Unaweza sema kama hii msemwa ni …***	3 = hakika kweli,<br>2 = kweli kidogo,<br>1 = siyo kweli kwa sana<br>888 = hayuwe<br>999 = akuna jibu	Kama "siyo kweli kwa sana" ao "hajuwi", pita kwa ulizo C5
C3	Nani unayekamata sawa muongozi / mushauri ?	1=mama,<br>2=baba<br>3=tate mwanamuke,<br>4=tate mwanaume,<br>5=dada,<br>6=kaka,<br>7=shangazi,<br>8=mujomba,<br>9=mume,<br>10= Kijana mpenzi,<br>11=mwengene mutu muzima,<br>12=kiongozi wa mpango pamoja na wa binti.<br>888 = hayuwe<br>999 = akuna jibu	

Horoza ya maulizo kwa wabinti - SWAHILI			
**Q#**	**Ulizo**	**Jibu**	**Kanuni za kufuata**
C4	Kuhusu nini muna jadiliyana/munaongeya pamoja na muongozi wako?	1=miradi ya usoni,<br>2=shida ya afia,<br>3=somo la shule,<br>4=wenzenu ku masomo<br>5=shida za jamaa,<br>6=kutosikilizana na wazazi/wasimamizi,<br>7=kutosikilizana na mume wako, mwanaume, ao kijana mpenzi,<br>8=kutosikilizana na warafiki na wajirani,<br>9= kutosikilizana na wakaka na wadada,<br>10 = kutosikilizana ku kazi,<br>11=mpango ya pesa/akiba,<br>12 = mpangilio wa kazi,<br>13 = mpangilio unayo na kijana mpenzi ao na mume wako<br>888 = hayuwe<br>999 = akuna jibu	Apana kusoma jibu zote, chagua majibu zote zinazo oneshwa na mwenyi kujibu.
C5	**Kiisha hapo, nitakuuliza namna gani unajisikia kwa kuzungumuzia jambo zifuatazo na wazazi/wasimamizi wako wenyi kuwa mwa hii kikundi. Kwa kila jambo ninalo taja, ninapenda uniambiye kama unajisikiya vizuri ama haujisikiye vizuri kwa kuya ongeya. Ni vizuri kama haujuwi ama kama haupendi kujibu. Je, unasikiya ?**		
C5a	Je, unajisikiya vizuri ama vibaya kwa kuongeya kuhusu masomo yako pamoja na wazazi/wasimamizi wako?	1 = vizuri<br>2 = vibaya<br>888 = hayuwe<br>999 = akuna jibu	
C5b	Na kuhusu Namana gani utapata/utafaulu maisha siku zijazo ?	1 = vizuri<br>2 = vibaya<br>888 = hayuwe<br>999 = akuna jibu	
C5c	Na kuhusu ndoa na wakati wazazi wanatarajia kama unaweza olewa?	1 = vizuri<br>2 = vibaya<br>888 = hayuwe<br>999 = akuna jibu	
C5d	Na kuhusu mabadiliko yanayo onekana kwa watoto wanapo anza kukomaa?	1 = vizuri<br>2 = vibaya<br>888 = hayuwe<br>999 = akuna jibu	
C5e	Je unajisikiya vizuri ama vibaya kwa kuongeya na wazazi/wasimamizi wako kuhusu viungo vya uzazi?	1 = vizuri<br>2 = vibaya<br>888 = Hayuwe<br>999 = akuna jibu	
C5f	Na kuhusu ginsi ya kufanya kwa kuhepuka kubeba mimba?	1 = vizuri<br>2 = vibaya<br>888 = hayuwe<br>999 = akuna jibu	
C5g	Na kuhusu VIH/SIDA (ukimwi) ao magonjwa mengine ya kuambukiya kwa njia ya ngono (MST)	1 = vizuri<br>2 = vibaya<br>888 = hayuwe<br>999 = akuna jibu	
**D. Usalama**	**Maulizo yafuatayo zina lingana na nia zako kuhusu usalama wako katika maisha yako, katika jamii yako na mahali ambapo unajisikiya ukiwa katika usalama**		
D1	Je, unajisikiya katika usalama nyumbani kwenu?	1 = ndiyo<br>2 = apana<br>888 = hayuwe<br>999 = akuna jibu	

Horoza ya maulizo kwa wabinti - SWAHILI			
**Q#**	**Ulizo**	**Jibu**	**Kanuni za kufuata**
D2	Je, unajisikiya katika usalama ku masomo ?	1 = ndiyo<br>2 = apana<br>888 = hayuwe<br>999 = akuna jibu	Kama walisema ndiyo ku swali B2, usi ulize swali hiyi.
D3	Je, unajisikiya katika usalama ku makao ya rafiki ?	1 = ndiyo<br>2 = apana<br>888 = hayuwe<br>999 = akuna jibu	
D4	Je, unajisikiya katika usalama ku makao ya jirani ?	1 = ndiyo<br>2 = apana<br>888 = hayuwe<br>999 = akuna jibu	
D5	Je, kuna nafasi ndani ya jamii ambapo unajisikiya vizuri kwa kukutana na wengine mabinti?	1 = ndiyo<br>2 = apana<br>888 = hayuwe<br>999 = akuna jibu	
	**Sasa, nilitaka nikuulize maswali fulani kuhusu marafiki zako. Nitasema misemwa Fulani Fulani, kwa kila moja, asante kwa kuniambiya kama unakubali ao kama haukubali. Ni sawa kama haujuwi ao kama haupendi jibu. Je unasikiya ?**		
D6	Nina marafiki ambao tunaweza zungumuza pamoja mambo ya lazima. Unakubali ama haukubali?	1 = nakubali<br>2 = sikubali<br>888 = hayuwe<br>999 = akuna jibu	
D7	Nina marafiki ambao ninaweza tegemea kwa musaada wa kimafikiri	1 = nakubali<br>2 = sikubali<br>888 = hayuwe<br>999 = akuna jibu	
D8	Una marafiki wa kike wa umri yako wa inje ya jamaa?	1 = nakubali<br>2 = sikubali<br>888 = hayuwe<br>999 = akuna jibu	Kama hapana ao hajuwi, enda kwa ulizo D10.
D9	Una warafiki wa kike wangapi?	0 = hakuna,<br>1= moja mpaka tatu,<br>2 = ine mpaka kumi,<br>3 = kumi na kupita,<br>888 = hayuwe<br>999 = hakuna jibu	
D10	Kutosha mutu fulani wa jamaa lako, kuna mutu mwanamuke mwenyi unaweza kwenda kwake mara kwa mara kueleza shida zako ?	1 = ndiyo,<br>0 = hapana,<br>888 = hayuwe<br>999 = akuna jibu	
**E. Utarajio / hali**	**Sasa, nitakuuliza maulizo Fulani fulani kuhusu yale yanayo fikiya binti katika maisha yake, mfano masomo, ndoa**		
E1	Ngazi gani ya masomo binti anapashwa eneza mbele ya kutoka kwenye shule?	__________(ngazi ya masomo)<br>888 = hayuwe<br>999 = akuna jibu	
E2	Kwa umri gani unafikiri binti anaweza kuolewa ?	__________(mwaka)<br>888 = hayuwe<br>999 = akuna jibu	
E3	Kwa umri gani unafikiri kwamba binti anaweza kuzaa mtoto wake wa kwanza ?	___________(mwaka)<br>888 = hayuwe<br>999 = akuna jibu	
**G. Kujitumainia**	**Karibu kwa kutumikisha tablette ACASI. Ninaitwa............................na nitakusomeya maulizo. Kumbuka kama utaweza ita mtafiti (enquêteur) haizuru saa gani kama una maulizo ama matatizo na ordinateur/tablette**<br><br>**Kwa kuanza, nitakusomea misemwa kumi ambazo zitaweza kukuelekeya saa zimoja. Tafazali, niambiye kwa kiasi gani unakubali hiyo msemwa : kama unakubali sana, unakubali, haukubali ao haukubali sana. Ni sawa kama haujuwi ao kama haupendi jibu. Je, unasikiya ?**		

Horoza ya maulizo kwa wabinti - SWAHILI			
**Q#**	**Ulizo**	**Jibu**	**Kanuni za kufuata**
G1	Ninajisikiya kuwa kwa ngazi sawa sawa na wengine	4 = Nakubali sana zaidi<br>3 = Nakubali<br>2 = Sikubali<br>1 = Sikubali sana<br>888 = hayuwe<br>999 = Akuna jibu	
G2	Ninajisikiya kuwa na tabiya nzuri sana	4 = Nakubali sana zaidi<br>3 = Nakubali<br>2 = Sikubali<br>1 = Sikubali sana<br>888 = hayuwe<br>999 = Akuna jibu	
G3	Niko tayari kujisikiya ya kwamba mimi mina shindwa	4 = Nakubali sana zaidi<br>3 = Nakubali<br>2 = Sikubali<br>1 = Sikubali sana<br>888 = hayuwe<br>999 = Akuna jibu	
G4	Nina uwezo wa kufanya vitu muzuri kushinda watu wengine	4 = Nakubali sana zaidi<br>3 = Nakubali<br>2 = Sikubali<br>1 = Sikubali sana<br>888 = hayuwe<br>999 = Akuna jibu	
G5	Najisikiya kuwa na sheriya ya kujivuna (kama nina samani)	4 = Nakubali sana zaidi<br>3 = Nakubali<br>2 = Sikubali<br>1 = Sikubali sana<br>888 = hayuwe<br>999 = Akuna jibu	
G6	Ninakuwa na hali ya kujitumainia mwenyewe	4 = Nakubali sana zaidi<br>3 = Nakubali<br>2 = Sikubali<br>1 = Sikubali sana<br>888 = hayuwe<br>999 = Akuna jibu	
G7	Ninatosheka na mimi peke yangu	4 = Nakubali sana zaidi<br>3 = Nakubali<br>2 = Sikubali<br>1 = Sikubali sana<br>888 = hayuwe<br>999 = Akuna jibu	
G8	Nina heshima nyingi kwangu mwenyewe	4 = Nakubali sana zaidi<br>3 = Nakubali<br>2 = Sikubali<br>1 = Sikubali sana<br>888 = hayuwe<br>999 = Akuna jibu	
G9	Najisikia kuwa mtu wa kutumainiya	4 = Nakubali sana zaidi<br>3 = Nakubali<br>2 = Sikubali<br>1 = Sikubali sana<br>888 = hayuwe<br>999 = Akuna jibu	
G10	Nina waza kama niko mtu wa samani (wa maana)	4 = Nakubali sana zaidi<br>3 = Nakubali<br>2 = Sikubali<br>1 = Sikubali sana<br>888 = hayuwe<br>999 = Akuna jibu	

Horoza ya maulizo kwa wabinti - SWAHILI			
**Q#**	**Ulizo**	**Jibu**	**Kanuni za kufuata**
**I. mawazo kuhusu ujehuri wa nyumbani**	**Mara na mara mume ana jizuru ao kuuzika na vitendo vya muke wake. Nitakusomeya misemwa fulani fulani. Kwa kila hali, ningependa uniambiye kama mume iko na sheria ya kumpiga muke wake. Kumbuka ya kwamba akuna jibu nzuri ao mbaya na kama utaweza sema haujuwi ao haupendi jibia haizuru wakati gani.**		
I1	Je, inakubaliwa kwa mume kumpiga mke wake kama anatoka nyumbani bila kumwambiya ?	1 = Ndiyo<br>2 = Apana<br>888 = hayuwe<br>999 = Akuna jibu	
I2	Je, inakubaliwa kwa mume kumpiga mke wake kama hashurulikiye watoto wake vizuri ?	1 = Ndiyo<br>2 = Apana<br>888 = hayuwe<br>999 = Akuna jibu	
I3	Je, inakubaliwa kwa mume kumpiga mke wake kama anafanya ubishi naye ?	1 = Ndiyo<br>2 = Apana<br>888 = hayuwe<br>999 = Akuna jibu	
I4	Je, inakubaliwa kwa mume kumpiga mke wake kama anakataa kufanya kitendo cha ndoa naye ?	1 = Ndiyo<br>2 = Apana<br>888 = hayuwe<br>999 = Akuna jibu	
I5	Je, inakubaliwa kwa mume kumpiga mke wake kama anaunguza chakula ku moto ?	1 = Ndiyo<br>2 = Apana<br>888 = hayuwe<br>999 = Akuna jibu	
**J. Ujehuri wa mwili, kupitisha kipimo kwa kujeuri kimafikiri na kwa kusema**	**Kipindi kifuatacho kya maulizo, inahusu vitu vingine ambavyo vinaweza kuwa vilishaka kufikia. Tunasikia ya kwamba ulizo moja moja ni za kipekee na zinaweza tuma unajisikiya vibaya, lakini tuna matumaini ya kwamba unachangia habari na sisi kusudi tujuwe mahitaji za wabinti kama wewe. Juwa ya kwamba jina lako halipatikani ndani ya utafiti na ginsi unatumikisha ordinateur hii, akuna mtu atakaye juwa majibu zako. Kumbuka ya kwamba yote yanayotendeka kwako siyo kosa lako. Ujisikiye huru kwa kujibu kwa wazi na kwa unaminifu, na haulazimishwe kupana jibu kama haujisikiye huru.**		
J1	Katika myezi 12 iliyopita, mtu fulani amekwisha kukupiga, kukupiga kofi kusudi ya kukuumiza ku mwili ?	1 = Ndiyo<br>2 = Apana<br>888 = hayuwe<br>999 = Akuna jibu	Kama « apana » ao « hajuwi », pita kwenye ulizo J3
J2	Nani ambaye alikuumiza kwa hali kama hii ?	1 = kijana mpenzi ao bwana<br>2 = Mzazi ao msimamizi<br>3 = Mtu mwengine wa jamaa<br>4 = Rafiki ao jirani<br>5 = Mwanamemba wa kikundi kinacho miliki silaha<br>6 = Mkuu wa mgini (kiongozi)<br>7 = Mutu mwengine<br>999 = Akuna jibu	Cochez tout ce qui convient
J3	Kwa miezi 12 iliyo pita, kumekuwa mtu fulani ambaye aliweza kuku hamakia kwa sauti kubwa ao kwa ushambulizi ?	1 = Ndiyo<br>2 = Apana<br>888 = hayuwe<br>999 = Akuna jibu	Kama « apana » ao « hajuwi », pita kwenye ulizo J5
J4	Nani ambaye aliweza kuku hamakia kwa sauti kubwa ao kwa ushambulizi?	1 = kijana mpenzi ao bwana<br>2 = Mzazi ao msimamizi<br>3 = Mtu mwengine wa jamaa<br>4 = Rafiki ao jirani<br>5 = Mwanamemba wa kikundi kinacho miliki silaha<br>6 = Mkuu wa mgini (kiongozi)<br>7 = Autre<br>999 = Akuna jibu	Cochez tout ce qui convient

**Horoza ya maulizo kwa wabinti - SWAHILI**			
**Q#**	**Ulizo**	**Jibu**	**Kanuni za kufuata**
J5	Kwa miezi 12 iliyo pita, mtu fulani aliweza kutukana, alikuambiya mambo mabaya, ao alikulainia?	1 = Ndiyo<br>2 = Apana<br>888 = hayuwe<br>999 = Akuna jibu	Kama « apana » ao « hajuwi », pita kwenye ulizo J7
J6	Nani ambaye aliweza kukutukana, alikuambiya mambo mabaya, ao alikulainia?	1 = kijana mpenzi ao bwana<br>2 = Mzazi ao msimamizi<br>3 = Mtu mwengine wa jamaa<br>4 = Rafiki ao jirani<br>5 = Mwanamemba wa kikundi kinacho miliki silaha<br>6 = Mkuu wa mgini (kiongozi)<br>7 = Autre<br>999 = Akuna jibu	Cochez tout ce qui convient
J7	Ndani ya myezi 12 iliyopita, umewai kujisikia kwamba mtu ambaye anapashwa kushurulikiya hakukushurulikia ?	1 = Ndiyo<br>2 = Apana<br>888 = hayuwe<br>999 = Akuna jibu	Kama « apana » ao « hajuwi », pita kwenye Kipindi "K"
J8	Ndani ya myezi 12 iliopita, umesikia kama hawa jiusishi na wewe?	1 = Mara nyingi<br>2 = Mara moja moja<br>3 = Hata mara moja<br>888 = hayuwe<br>999 = Akuna jibu	
**K. Ujehuri wa viungo vya uzazi**	**Asante kwa kujibu kwa hayo maulizo kuhusu vitu ambavyo ulipitiya. Najuwa ya kwamba kujibu kwa hayo mitindo ya maulizo haiko rahisi. Kipindi cha kufwata kinapatikana pia na maulizo ngumu, lakini tunatumaini kama kwa kutumikisha ordinateur/tablette utajisikiya huru kwa kutuambiya yale uliyo yapitiya.**<br><br>**Wabinti na wanawake wanaweza patwa na kukutana kimapenzi ambayo hawa taki kutokana na watu ambao wanafahamu vizuri, kama mpendwa, mwanamemba wa jamaa ao rafiki, ao mtu ambaye hajulikani. Maswali ndani ya hiki kipindi niza kipekee, na zingeweza tuma haujisikiye vizuri kwaku jibia, lakini majibu yako yata tusaidia kwakuelewa yale wengine walipitia kufuatana nakukutana kimapenzi bila ruhusa yao. Majibu yenu yanakuwa ya siri na muna weza ruka maulizo zenye hamutaki kujibia. Pia mukumbuke kwamba hamuta hukumiwa, na hakuna majibu mazuri ao mabaya.**<br><br>**Kumbuka ya kwamba yote yaliyo tendeka kwako na mtu mkubwa bila kutaka ao ruhusa yako siyo kosa lako**		
K1	Je, mutu fulani amekwisha kugusa kwa hali ya kimapenzi bila ruhusa yako ?<br>Kugusa kwa hali ya kimapenzi na kuongeza kupapasa, kufinya, kuyambiliya	1 = Ndiyo<br>2 = Apana<br>888 = hayuwe<br>999 = Akuna jibu	Kama « apana » ao « hajuwi », pita kwenye ulizo K4
K2	Nani allitenda vile ?	1 = kijana mpenzi ao bwana<br>2 = Mzazi ao msimamizi<br>3 = Mtu mwengine wa jamaa<br>4 = Rafiki ao jirani<br>5 = Mwanamemba wa kikundi kinacho miliki silaha<br>6 = Mkuu wa mgini (kiongozi)<br>7 = Mutu mwengine<br>999 = Akuna jibu	Cochez tout ce qui convient
K3a	Kwa muda ya miezi 12 ilio pita, kuna mutu aliku gusa kwa hali yaki mapenzi bila ruhusa yako ?	1 = Ndio<br>2 = Apana<br>888 = hayuwe<br>999 = Akuna jibu	

Horoza ya maulizo kwa wabinti - SWAHILI			
**Q#**	**Ulizo**	**Jibu**	**Kanuni za kufuata**
K3	Katika miezi 12 iliyopita, ni mara ngapi mtu amekwisha kugusa kwa hali ya kimapenzi bila ruhusa yako ?	__________<br>888 = hayuwe<br>999 = Akuna jibu	
K4	Kunamutu alisha wai kukushurtisha kutenda kitendo cha ndoa pamoja naye, kwa kutumikisha madaraka, ao kukuogopesha? kwa mufano, kwa kusema kwamba atakushindisha ku masomo ao kwamba atakufanyiya kitu ingine mbaya ?	1 = Ndiyo<br>2 = Apana<br>888 = hayuwe<br>999 = Akuna jibu	Kama « apana » ao « hajuwi », pita kwenye ulizo K7
K5	Nani alitenda vile ?	1 = kijana mpenzi ao bwana<br>2 = Mzazi ao msimamizi<br>3 = Mtu mwengine wa jamaa<br>4 = Rafiki ao jirani<br>5 = Mwanamemba wa kikundi kinacho miliki silaha<br>6 = Mkuu wa mgini (kiongozi)<br>7 = Mutu mwengin<br>999 = Akuna jibu	Cochez tout ce qui convient
K6a	Katika myezi 12 iliyopita ilikufiia kutenda kitendo cha ndoa na mutu fulani kwa sababu aliku shurutisha kwa maneno makali wala kwaku tumikisha ukubwa wake ?	1 = Ndiyo<br>2 = Apana<br>888 = hayuwe<br>999 = Akuna jibu	
K6	Katika myezi 12 iliyopita mara ngapi umetenda kitendo cha ndoa na mutu fulani kwa sababu aliku shurutisha kwa maneno makali wala kwaku tumikisha ukubwa wake ?	__________<br>888 = hayuwe<br>999 = Akuna jibu	
K7	Umewai tayari kuwa na kitendo cha ndoa pamoja na mwanamume ambae munaishi naye kwa sababu upate pesa ao vingine vitu kama chakula ao pesa?	1 = Ndiyo<br>2 = Apana<br>888 = hayuwe<br>999 = Akuna jibu	Kama « apana » ao « hajuwi », pita kwenye ulizo K9
K8	Haya yame kufikiya ndani ya myezi 12 iliyopita ?	1 = Ndiyo<br>2 = Apana<br>888 = hayuwe<br>999 = Akuna jibu	
K9	Kuna mutu amewai kukupa chakula, zawadi ao haizuru fazili gani nyingine kwa ubadilishaji kwa kutenda kitendo cha ndoa pamoja naye ?	1 = Ndiyo<br>2 = Apana<br>888 = hayuwe<br>999 = Akuna jibu	Kama « apana » ao « hajuwi », pita kwenye ulizo K11
K10	Haya yame kufikiya ndani ya myezi 12 iliyopita?	1 = Ndiyo<br>2 = Apana<br>888 = hayuwe<br>999 = Akuna jibu	
	**Ukipendezwa uniambiye kama unakubali ao haukubali misemwa haya. Kumbuka ya kuwa akuna jibu nzuri ao mbaya na kama unaweza sema kama haujuwi ao kama haupendi jibia.**		
K11	Nitakaripiwa na jamaa langu kama ninge lazimishwa kufanya kitendo cha ndoa	1 = Nakubali<br>2 = Sikubali<br>888 = hayuwe<br>999 = Akuna jibu	
K12	Jamii itaweza nilazimisha niolewe na mwanaume kama alinilazimisha kutenda kitendo cha ndoa naye	1 = Nakubali<br>2 = Sikubali<br>888 = hayuwe<br>999 = Akuna jibu	

Horoza ya maulizo kwa wabinti - SWAHILI			
**Q#**	**Ulizo**	**Jibu**	**Kanuni za kufuata**
K13	Nina mutu fulani ndani ya jamii ambaye ninaweza tumainia na ambaye nitaweza zungumuzia kama nimejikuta kulazimishwa kufanya kitendo cha ndoa	1 = Nakubali<br>2 = Sikubali<br>888 = hayuwe<br>999 = Akuna jibu	
**L. Huduma**	**Asante kwakuweza jibu kwa maulizo haya ngumu, ulifanya kazi nzuri sana. Ninaku ahidiya kama akutakuwa tena maulizo kama na yale kuhusu yale uliyo yapitiya. Na sasa, nita kuuliza maulizo kuhusu huduma ambazo zinaweza patikana kwa wabinti kama wewe.**		
L1	Kama mtu fulani ali tenda wala alijaribu kutenda kitendo cha ndoa na binti fulani bila kutaka kwake, unajuwa mahali ambako anaweza enda kupata msaada ao maelekezo?	1 = Ndiyo<br>2 = Apana<br>888 = hayuwe<br>999 = Akuna jibu	
L2	Je, unajuwa mahali binti anaweza jielekeza kama kuna mtu aliyemupiga ?	1 = Ndiyo<br>2 = Apana<br>888 = hayuwe<br>999 = Akuna jibu	
**M. Matumaini na maelekeo ya siku zijazo**	**Sasa nitasoma misemwa zinazohusu ginsi gani unawaza ju yako mwenyewe. Ninapenda uniambiye kwa kila moja ni kwa kiasi gani unawaza vile – kama hauwaze hata vile, muda kidogo, wakati fulani fulani, wakati nyingi ao wakati nyingi sana**<br><br>**Kama kawaida, kumbuka kama akuna jibu nzuri ao mbaya na utaweza sema kama haujuwi ao kukataa kujibia kwa kila ulizo**		
M1	Nafikiri kama niko vizuri (vitu vinaendeka kwangu)<br><br>Ni kwa kiasi gani unawaza hivi ?	1 = Akuna muda<br>2 = Muda kidogo sana<br>3 = Wakati fulani fulani<br>4 = Wakati nyingi<br>5 = Wakati nyingi sana<br>888 = hayuwe<br>999 = Akuna jibu	
M2	Naweza fikiriya njia nyingi za kupata vitu vilivyo vya lazima kwangu ndani ya maisha<br><br>Ni kwa kiasi gani unawaza hivi ?	1 = Akuna muda<br>2 = Muda kidogo sana<br>3 = Wakati fulani fulani<br>4 = Wakati nyingi<br>5 = Wakati nyingi sana<br>888 = hayuwe<br>999 = Akuna jibu	
M3	Niko na uwezo wa kufaulu (kuweza) sawa sawa na wengine watoto wa umri yangu<br><br>Ni kwa kiasi gani unawaza hivi ?	1 = Akuna muda<br>2 = Muda kidogo sana<br>3 = Wakati fulani fulani<br>4 = Wakati nyingi<br>5 = Wakati nyingi sana<br>888 = hayuwe<br>999 = Akuna jibu	
M4	Wakati niko na shida naweza pata njia nyingi zakupata jibu<br><br>Ni kwa kiasi gani unawaza hivi ?	1 = Akuna muda<br>2 = Muda kidogo sana<br>3 = Wakati fulani fulani<br>4 = Wakati nyingi<br>5 = Wakati nyingi sana<br>888 = hayuwe<br>999 = Akuna jibu	
M5	Nawaza kama mambo niliyo fanya hapo zamani yata nisaidiya siku zijazo<br><br>Ni kwa kiasi gani unawaza hivi ?	1 = Akuna muda<br>2 = Muda kidogo sana<br>3 = Wakati fulani fulani<br>4 = Wakati nyingi<br>5 = Wakati nyingi sana<br>888 = hayuwe<br>999 = Akuna jibu	

Horoza ya maulizo kwa wabinti - SWAHILI			
**Q#**	**Ulizo**	**Jibu**	**Kanuni za kufuata**
M6	Hata kama wengine wana tarajiya kuacha, najua kama nitaweza pata njia za suluhisho kwa shida<br><br>Ni kwa kiasi gani unawaza hivi ?	1 = Akuna muda<br>2 = Muda kidogo sana<br>3 = Wakati fulani fulani<br>4 = Wakati nyingi<br>5 = Wakati nyingi sana<br>888 = hayuwe<br>999 = Akuna jibu	
**N. ulizo za mwisho**	**Asante kwa kujibu kwa maulizo haya. Najuwa kama zimoja zimoja zilikuwa nguvu. Kumbuka kama akuna mtu wa jamii yako ambaye atajuwa yale uliyojibu. Ni mwisho wa kutumikisha ordinateur/tablette, ukipendezwa, uite mtafiti na umpatiye ayo.**<br>(UNE FOIS L'ENFANT A TERMINE, REPRENEZ LA TABLETTE ACASI. VERIFIEZ SI TOUTES LES CHOSES SONT CORRECTES ET ALORS LIT LA DÉCLARATION EN DESSOUS:)<br>**Asante kwa kujibu kwa maulizo. Ulifanya kazi nzuri kwa kutumikisha ordinateur na kwa kujibu kwenyi maulizo ngumu.**<br>**Nitakuuliza sasa maulizo nyepesi yenyi kuhusu yale unayopenda fanya**		
**N1**	Ni kazi gani unafurahiyaka kufanya ndani ya kijiji chako ?		
**O. Ulizo za mwisho wa utafiti**	**Tumekwisha karibuni kumaliza.**<br>**Mbele ya kumaliza mazungumuzo yetu, ningaliki na ulizo za kukuuliza kuhusu ginsi ulijisikiya wakati wa maulizo**		
O1	Ulizo ambazo umekwisha jibu zillikuwa …	1 = Nyepesi kwa kusikia<br>2 = Nyepesi kiyasi kusikia<br>3 = Ngumu kwa kusikia<br>4 = Kweli ngumu kwa kusikia<br>888 = hayuwe<br>999 = Akuna jibu	
O2	Kwa jumla, kwa ngazi gani ya uaminifu una weza sema ya kwamba ulijibu kwa maulizo ?	1 = Si kwa uaminifu zaidi<br>2 = Si kwa uaminifu<br>3 = Uaminifu kiyasi<br>4 = Kweli kwa uwaminifu<br>5 = Kwa uwaminifu kabisa	
O3	Kumekuwa mambo mengine ungependa ongeza ao ulizo ya kuniuliza ?		
O6	Kwakumaliza maswali ya maongezi hii, je, ulifurahiya kupana majibu hapohapo kwa muendesha uchunguzi ao kutumikisha tablet ?	1= Kupatiya muendesha uchunguzi majibu hapohapo<br>2= Akitumikisha Tablet<br>3= Hakuna yakufurahiya<br>888= Hayuwe<br>999=Hakuna jibu	

Horoza ya maulizo kwa wabinti - SWAHILI			
**Q#**	**Ulizo**	**Jibu**	**Kanuni za kufuata**
	**FAIRE UN DEBRIEF AVEC LA FILLE : : KUSHUKURU BINTI KWA MUDA NA UAMINIFU WAKE KUJIBU KU MAULZO. MUAMBIYE KWAMBA UNAELEWA KWAMBA MAULIZO ZILIKUWA NGUMU KWA KUJIBU HAIKUKUWA KWEPESI. UMUHAKIKISHIYE KWAMBA MAJIBU ZITAWEKWA KWA SIRI. MUJULISHE KWAMBA ANAWEZA WASILIANA NA KIKUNDI CHA UTAFITI HAIZURU WAKATI GANI KWA MAULIZO NA SHURULI**<br><br>**KUSOMA HAYA: Mahali hapa tunapopatikana ni nyumba ya OCB (NOM DE L'OCB) ambamo munapatikana wanawake wenye kupana huduma ya mazungumuzo za kipekee. Mmoja kati ya wanawake hao anapatikana karibu na nitaweza kukuongoza kwake saa hii kama unapenda ; kutosha hayo, unaweza rudi hapa wakati wote kwa kuongeya na mmoja kati ya wanawake hao, ao nitaweza kupatiya namba ya simu (téléphone)**<br><br>(DONNER LA LISTE DE CONTACT DES SERVICES. SI ELLE NE VEUT PAS PRENDRE LA LISTE INFORMEZ-LA QU'ELLE PEUT CONTACTER L'EQUIPE DE RECHERCHE N'IMPORTE QUAND POUR L'ASSISTANCE<br><br>DEMANDEZ-LUI SI ELLE A D'AUTRES QUESTIONS)		
**P. Questions pour l'enquêteuse**	**ENQUETEUR : MERCI DE REPONDRE AUX QUESTIONS SUIVANTES AVANT DE TERMINER L'INTERVIEW**		
P1	L'interviewée semblait-elle comprendre les questions?	1= Tout le temps<br>2<br>3= De temps en temps<br>4<br>5= Jamais	Choisir entre 1 et 5
P2	L'interviewée semblait-elle répondre aux questions de façon aléatoire?	1= Tout le temps<br>2<br>3= De temps en temps<br>4<br>5= Jamais	Choisir entre 1 et 5
P3	L'interviewée semblait-elle réfléchir a chaque réponse avant de répondre ?	1= Tout le temps<br>2<br>3= De temps en temps<br>4<br>5= Jamais	Choisir entre 1 et 5
P4	Y'avait-il quelqu'un d'autre présent au moment de l'interview ?	1=Oui<br><br>2=Non	Si "Oui", procéder à P4b et P4c<br>Si "Non", passer à P5
P4b	Qui était cette personne?	________	Ecrire seulement la relation avec l'interviewée
P4c	Jusqu'à quel niveau sentez-vous que la présence de cette personne influençait les réponses données par l'interviewée?	1= Beaucoup<br>2= Un peu<br>3= Très peu<br>4= Pas du tout	
P5	L'interview a-t-elle été interrompue pour une quelconque raison?	1=Oui<br><br>2=Non	Si "Oui", procéder à P5b et P5c<br>Si "Non", passer à P6
P5b	Pourquoi l'interview a-t-elle été interrompue?	______________	
P5c	Selon vous, cela a-t-il affecté l'interview?	1=Oui<br><br>2=Non	
P6	Quel était le contexte dans lequel l'interview a eu lieu?	1= Calme, privé<br>2= Quelque bruit, presque privé<br>3= Trop de bruit, des gens tout autour	
P7	Comment évalueriez-vous la capacité de l'interviewée de comprendre la plupart des questions?	1= N'a pas beaucoup compris<br>2= A compris un peu<br>3= A compris modérément<br>4= A beaucoup compris<br>5= A compris énormément	
P8	Quelles questions ont semblé plus		S'il vous plait, lister les questions

	difficile à comprendre pour l'interviewée ?		
P9	En général, comment évaluerez-vous le nivaur d'intérêt que l'interviewée portait à l'interview?	1= Vraiment haut<br>2= Au dessus de la moyenne<br>3= Moyenne<br>4= En dessous de la moyenne<br>5= Vraiment bas	
P10	Comment avez-vous trouvez l'utilisation de la tablette par la fille?		
P11	L'interviewée a-t-elle besoin d'une référence à un prestataire de services quelconque?		Si oui, décrire s'il vous plait.
	**FIN DU QUESTIONNAIRE, TRES BON TRAVAIL!**		
