# Supplementary material for: Gender-equitable caregiver attitudes and education and safety of adolescent girls in South Kivu, DRC: A secondary analysis from a randomized controlled trial
Source: PLoS Med. 2021 Sep 28;18(9):e1003619. doi: 10.1371/journal.pmed.1003619 (PMC8478225; doi:10.1371/journal.pmed.1003619)
Supplement: S4 Questionnaire — (PDF) [file pmed.1003619.s007.pdf]

**Caregivers' survey- French**

**QUANTITATIF ADULTES /FRENCH**

| Caregiver Survey (DRC) | | | |
|---|---|---|---|
| **Question #** | **Question** | **Réponse options** | **Instructions** |
| **A. Questions administratives** | | | |
| A1 | Nom du village/site | ____________________________ _____________ | Record |
| A2 | Quartier | __________________ | Use codes given by listing |
| A3 | Sous village du quartier | __________________ | Use codes given by listing |
| A4 | Langue parlé | 1=Swahili<br>2=Mashi | |
| A5 | Code d'enquêteur | _____ _____ | |
| A6 | Date de l'enquête | _________/_________/__________ __ | jj/mm/aaaa |
| A7 | Heure du début de l'enquête | ____________: ____________ | 24 heures |
| A8 | Heure de la fin de l'enquête | ____________: ____________ | 24 heures |
| A9 | Code d'identification du parent | _______________________ | |
| A10 | Code d'identification de la fille | _______________________ | |
| A11 | Age du parent/responsable | | 888=Ne sait pas |

| Caregiver Survey (DRC) | | | |
|---|---|---|---|
| **Question #** | **Question** | **Réponse options** | **Instructions** |
| **B. Informations Démographiques** | **Bonjour encore, ça me fait plaisir de vous voir. Merci d'avoir été d'accord l'autre jour pour nous rencontrer à cette interview. J'aimerais vous rappeler au sujet de ce que nous avons discuté la dernière fois qu'on s'est parlé. Nous allons vous poser des questions au sujet de vous-même et vos relations avec votre fille qui est inscrite dans ce programme, aussi bien que vos opinions sur certains sujets. Bien que ni vous ni votre fille bénéficiera personnellement de votre participation dans cette interview, ni recevra de l'argent ou autres avantages matériels, nous récoltons cette information pour améliorer des programmes des filles en RDC, et nous espérons que vous serez réfléchi et honnête en répondant.**<br><br>**Il n'y a aucune pression sur vous pour nous parler aujourd'hui. Vous pouvez dire que vous ne voulez pas nous parler aujourd'hui et nous ne serons pas offensés. Vous pouvez également refuser de répondre à toute question à la quelle vous ne voulez pas répondre si vous vous sentez gêné ou mal à l'aise, et rien de mauvais ne vous arrivera ou la fille inscrite dans ce programme; nous passerons tout simplement à la question suivante.**<br><br>**L'information que vous nous donnerez sera utilisée exclusivement pour le but de la recherche, et personne (y compris la fille, votre famille, amis, ou autres personnes dans votre communauté) ne saura ce que vous avez dit. Tout ce que vous nous dites sera gardé en secret.**<br><br>**Avant que nous commencions, je veux m'assurer que vous êtes à l'aise et que vous comprenez tout au sujet de ce que nous avons parlé. Est-ce que vous avez des questions? Est-ce que vous consentez à me parler aujourd'hui?**<br><br>**Génial. Nous commençons avec des questions faciles au sujet de vous. Quand nous utilisons l'expression « votre fille », nous voulons dire la fille inscrite dans ce programme, pour qui vous êtes le parent ou responsable.** | | |
| B1 | Quel est votre âge? | _____________ Ans<br>888= Ne sait pas<br>999 = Pas de réponse | SI MOINS DE 18 ANS, STOP |
| B4 | Qui est la personne principale qui soutient financièrement le ménage de votre fille ? | 1=Mère<br>2=Père<br>3=Grand parent<br>4=Beau parent<br>5=Frère ou sœur<br>6=Tante/Oncle<br>7=Autre membre de la famille<br>8=Responsable qui n'est pas membre de la famille<br>9=Autre<br>888= Ne sait pas<br>999 = Pas de réponse | Cocher seulement une réponse<br><br>Si "Responsable qui n'est pas membre de la famille" ou "Autre", préciser |

| **Caregiver Survey (DRC)** | | | |
|---|---|---|---|
| **Question #** | **Question** | **Réponse options** | **Instructions** |
| **C. Les normes des rôles de genre** | **Je voudrais maintenant vous poser quelques questions en rapport avec votre point de vue sur les filles et les garçons. Les gens ont des opinions différentes sur ces choses et il n'y a pas de bonnes ou de mauvaises réponses. Sachez que vos réponses ne seront pas jugées et que vous pouvez refuser de répondre à une question à n'importe quel moment sans conséquences pour vous ou votre fille.** | | |
| C1 | Il est important que les garçons soient plus éduqués que les filles. | 1=Oui<br>2=Non<br>888=Ne sait pas<br>999 =Pas de réponse | |
| C2 | Les garçons devraient être envoyées à l'école seulement si on n'a pas besoin de leur aide à la maison. | 1=Oui<br>2=Non<br>888=Ne sait pas<br>999 =Pas de réponse | |
| C3 | La raison la plus importante qui fait que les garçons devraient avoir plus d'accès à l'éducation que les filles c'est afin que les garçons puissent mieux prendre en charge leurs parents quand ils vieillissent. | 1=Oui<br>2=Non<br>888=Ne sait pas<br>999 =Pas de réponse | |
| C4 | S'il y a une somme d'argent limitée à utiliser pour les études, cette somme devrait être affectée prioritairement aux garçons. | 1=Oui<br>2=Non<br>888=Ne sait pas<br>999 =Pas de réponse | |
| C5 | Les femmes devraient laisser la politique aux hommes | 1=Oui<br>2=Non<br>888=Ne sait pas<br>999 =Pas de réponse | |
| C6 | Une femme doit avoir un mari ou un garçon ou un autre responsable masculin qui puisse la protéger parce qu'elle n'est pas capable de se protéger elle-même. | 1=Oui<br>2=Non<br>888=Ne sait pas<br>999 =Pas de réponse | |
| C7 | La seule chose sur laquelle une femme puisse compter dans sa vieillesse ce sont ses garçons. | 1=Oui<br>2=Non<br>888=Ne sait pas<br>999 =Pas de réponse | |
| C8 | Une bonne femme ne doute jamais de l'opinion de son mari, même si elle n'est pas certaine qu'elle est d'accord avec lui. | 1=Oui<br>2=Non<br>888=Ne sait pas<br>999 =Pas de réponse | |

| **Caregiver Survey (DRC)** | | | |
|---|---|---|---|
| **Question #** | **Question** | **Réponse options** | **Instructions** |
| C9 | Les filles devraient avoir les mêmes opportunités de travailler hors de la maison que les garçons. | 2=Oui<br>1=Non<br>888=Ne sait pas<br>999 =Pas de réponse | |
| C10 | J'aimerais que ma fille soit en mesure de trouver un travail hors de la maison pour qu'elle puisse prendre soin d'elle-même et si nécessaire de sa famille. | 2=Oui<br>1=Non<br>888=Ne sait pas<br>999 =Pas de réponse | |
| **D. Éducation de l'enfant** | | | |
| D1 | Croyez-vous que, afin de bien éduquer les filles, il faut les punir physiquement? | 1=Oui<br>2=Non<br>888=Ne sait pas<br>999 =Pas de réponse | |
| D2 | Jusqu'à quel niveau cette assertion est vraie :<br>Il est important que vous sachiez où se trouve votre fille à tout moment. | 3=Très vrai,<br>2=Un peu vrai<br>1=Pas du tout vrai<br>888=Ne sait pas<br>999 =Pas de réponse | |
| | **Maintenant, je vais vous poser certaines questions sur l'avenir que vous aimeriez voir pour votre fille. Rappelez-vous que pour n'importe quelle question vous pouvez dire que vous ne savez pas ou que vous ne voulez pas répondre. Est-ce que vous comprenez?** | | |
| D3 | Votre fille est-elle actuellement inscrite à l'école ? | 1=Oui<br>2=Non<br>888=Ne sait pas<br>999 =Pas de réponse | Si "Non" ou "Ne sait pas", passer a la question D6 |
| D4 | Quel niveau scolaire espérez-vous que votre fille pourrait atteindre avant de quitter l'école?<br><br>PASSER CETTE QUESTION SI LA FILLE N'EST PAS A L'ÉCOLE. | ___________(niveau scolaire)<br><br>888=Ne sait pas<br>999 =Pas de réponse | |
| D5 | Jusqu'à quel âge voudriez-vous que votre fille reste à l'école?<br><br>PASSER CETTE QUESTION SI LA FILLE N'EST PAS A L'ÉCOLE. | ___________(ans) | Écrire 88 si "Ne sait pas"<br>Écrire 99 si "Pas de réponse" |

| Caregiver Survey (DRC) | | | |
|---|---|---|---|
| **Question #** | **Question** | **Réponse options** | **Instructions** |
| D6 | A quel âge espérez-vous que votre fille pourrait se marier? | ___________(ans) | Écrire 88 si "Ne sait pas"<br>Écrire 99 si "Pas de réponse" |
| D7 | A quel âge espérez-vous que votre fille pourrait accoucher de son premier enfant? | ___________(ans) | Écrire 88 si "Ne sait pas"<br>Écrire 99 si "Pas de réponse" |
| D8 | Si une fille vous a dit qu'elle venait de subir une violence sexuelle, vous sentirez-vous à l'aise de lui parler à propos des services disponibles? | 1=Oui<br>2=Non<br>888=Ne sait pas<br>999 =Pas de réponse | Si "Non" ou « Ne sait pas », passer à D8b |
| D8a | Si oui, lesquels? | 1=Services médicaux<br>2=Services juridiques<br>3=Services psychologiques<br>4=Services socio-économiques<br>5=Gestion de cas à l'OCB (y compris le counseling individuel, etc.)<br>6=Autre<br>888=Ne sait pas<br>999=Pas de réponse | Passer à E1 |
| D8b | Si non, pourquoi pas ? | 1= Je ne connais pas les services disponibles<br>2= Les services ne sont pas disponibles<br>3= Je m'en fiche, ce n'est pas ma responsabilité<br>4= Les services ne sont pas de bonne qualité<br>5=Les services existent mais ne sont pas accessibles (financièrement, géographiquement, etc.)<br>6=Autre<br>888=Ne sait pas<br>999=Pas de réponse | |
| **E. Comportement parental** | **Maintenant, je vais vous lire quelques assertions sur comment vous communiquez avec vos enfants. Je veux que vous me disiez pour chacune la fréquence avec laquelle c'est vrai: c'est presque toujours vrai, parfois vrai, rarement vrai, ou presque jamais vrai. Vous pouvez dire que vous ne savez pas ou vous pouvez refuser de répondre à une question à n'importe quel moment sans conséquence.** | | |

| Caregiver Survey (DRC) | | | |
|---|---|---|---|
| **Question #** | **Question** | **Réponse options** | **Instructions** |
| E1 | Je dis des bonnes choses concernant mon enfant. | 1=Presque toujours vrai<br>2=Parfois vrai<br>3=Rarement vrai<br>4=Presque jamais vrai<br>888=Ne sait pas<br>999=Pas de réponse | |
| E2 | Je ne prête pas attention à mon enfant. | 1=Presque toujours vrai<br>2=Parfois vrai<br>3=rarement vrai<br>4=Presque jamais vrai<br>888=Ne sait pas<br>999=Pas de réponse | |
| E3 | Je facilite mon enfant de se confier à moi. | 1=Presque toujours vrai<br>2=Parfois vrai<br>3=rarement vrai<br>4=Presque jamais vrai<br>888=Ne sait pas<br>999=Pas de réponse | |
| E4 | Je frappe mon enfant même quand il/elle ne le mérite pas. | 1=Presque toujours vrai<br>2=Parfois vrai<br>3=rarement vrai<br>4=Presque jamais vrai<br>888=Ne sait pas<br>999=Pas de réponse | |
| E5 | Je vois mon enfant comme une grande nuisance | 1=Presque toujours vrai<br>2=Parfois vrai<br>3=rarement vrai<br>4=Presque jamais vrai<br>888=Ne sait pas<br>999=Pas de réponse | |
| E6 | Je punis mon enfant quand je suis en colère. | 1=Presque toujours vrai<br>2=Parfois vrai<br>3=rarement vrai<br>4=Presque jamais vrai<br>888=Ne sait pas<br>999=Pas de réponse | |
| E7 | Je suis trop occupé par d'autres choses pour répondre aux questions de mon enfant. | 1=Presque toujours vrai<br>2=Parfois vrai<br>3=rarement vrai<br>4=Presque jamais vrai<br>888=Ne sait pas<br>999=Pas de réponse | |

| Caregiver Survey (DRC) | | | |
|---|---|---|---|
| **Question #** | **Question** | **Réponse options** | **Instructions** |
| E8 | J'éprouve du ressentiment contre mon enfant | 1=Presque toujours vrai<br>2=Parfois vrai<br>3=rarement vrai<br>4=Presque jamais vrai<br>888=Ne sait pas<br>999=Pas de réponse | |
| E9 | Je suis vraiment intéressé de ce que mon enfant fait. | 1=Presque toujours vrai<br>2=Parfois vrai<br>3=rarement vrai<br>4=Presque jamais vrai<br>888=Ne sait pas<br>999=Pas de réponse | |
| E10 | Je dis beaucoup de choses qui ne sont pas bonnes à mon enfant | 1=Presque toujours vrai<br>2=Parfois vrai<br>3=rarement vrai<br>4=Presque jamais vrai<br>888=Ne sait pas<br>999=Pas de réponse | |
| E11 | Je ne réagis pas quand mon enfant me demande de l'aide. | 1=Presque toujours vrai<br>2=Parfois vrai<br>3=rarement vrai<br>4=Presque jamais vrai<br>888=Ne sait pas<br>999=Pas de réponse | |
| E12 | Je m'assure que mon enfant se sente voulu et important | 1=Presque toujours vrai<br>2=Parfois vrai<br>3=rarement vrai<br>4=Presque jamais vrai<br>888=Ne sait pas<br>999=Pas de réponse | |
| E13 | Je prête beaucoup d'attention à mon enfant | 1=Presque toujours vrai<br>2=Parfois vrai<br>3=rarement vrai<br>4=Presque jamais vrai<br>888=Ne sait pas<br>999=Pas de réponse | |
| E14 | Je fais de la peine à mon enfant | 1=Presque toujours vrai<br>2=Parfois vrai<br>3=rarement vrai<br>4=Presque jamais vrai<br>888=Ne sait pas<br>999=Pas de réponse | |

| Caregiver Survey (DRC) | | | |
|---|---|---|---|
| **Question #** | **Question** | **Réponse options** | **Instructions** |
| E15 | Je fais que mon enfant se sente rejeté si il/elle se comporte mal. | 1=Presque toujours vrai<br>2=Parfois vrai<br>3=rarement vrai<br>4=Presque jamais vrai<br>888=Ne sait pas<br>999=Pas de réponse | |
| E16 | J'oublie les choses importantes que mon enfant pense que je devrais me souvenir. | 1=Presque toujours vrai<br>2=Parfois vrai<br>3=rarement vrai<br>4=Presque jamais vrai<br>888=Ne sait pas<br>999=Pas de réponse | |
| E17 | Je fais que mon enfant sente que ce qu'il fait est important. | 1=Presque toujours vrai<br>2=Parfois vrai<br>3=rarement vrai<br>4=Presque jamais vrai<br>888=Ne sait pas<br>999=Pas de réponse | |
| E18 | Lorsque mon enfant fait quelque chose de mal, je lui fais peur ou le menace. | 1=Presque toujours vrai<br>2=Parfois vrai<br>3=rarement vrai<br>4=Presque jamais vrai<br>888=Ne sait pas<br>999=Pas de réponse | |
| E19 | Je tiens compte de ce que mon enfant pense, et je l'encourage d'en parler. | 1=Presque toujours vrai<br>2=Parfois vrai<br>3=rarement vrai<br>4=Presque jamais vrai<br>888=Ne sait pas<br>999=Pas de réponse | |
| E20 | Je ressens que les autres enfants sont meilleurs que le mien | 1=Presque toujours vrai<br>2=Parfois vrai<br>3=rarement vrai<br>4=Presque jamais vrai<br>888=Ne sait pas<br>999=Pas de réponse | |
| E21 | Je fais savoir à mon enfant que je ne veux pas de lui/elle. | 1=Presque toujours vrai<br>2=Parfois vrai<br>3=rarement vrai<br>4=Presque jamais vrai<br>888=Ne sait pas<br>999=Pas de réponse | |

| **Caregiver Survey (DRC)** | | | |
|---|---|---|---|
| **Question #** | **Question** | **Réponse options** | **Instructions** |
| E22 | Je fais savoir à mon enfant que je l'aime | 1=Presque toujours vrai<br>2=Parfois vrai<br>3=rarement vrai<br>4=Presque jamais vrai<br>888=Ne sait pas<br>999=Pas de réponse | |
| E23 | Je ne prête pas attention à mon enfant tant qu'il /elle ne m'embête pas | 1=Presque toujours vrai<br>2=Parfois vrai<br>3=rarement vrai<br>4=Presque jamais vrai<br>888=Ne sait pas<br>999=Pas de réponse | |
| E24 | Je traite mon enfant avec gentillesse | 1=Presque toujours vrai<br>2=Parfois vrai<br>3=rarement vrai<br>4=Presque jamais vrai<br>888=Ne sait pas<br>999=Pas de réponse | |
| **F. Attitudes à propos de la discipline** | **Parfois, quand les parents ou la personne qui s'occupe des enfants est fâché ou énervée par certaines choses que font les enfants, ils frappent (fort) ces derniers. Les gens ont des opinions différentes sur les parents qui frappent les enfants. Selon vous, quand est-ce que les parents ont le droit de frapper leurs enfants?** | | |
| F1 | Ils ont raison de les frapper… si l'enfant est désobéissant. | 1=Oui<br>2=Non<br>888=Ne sait pas<br>999 =Pas de réponse | |
| F2 | Ils ont raison de les frapper… si l'enfant n'est pas d'accord avec le parent. | 1=Oui<br>2=Non<br>888=Ne sait pas<br>999 =Pas de réponse | |
| F3 | Ils ont raison de les frapper… si l'enfant fuit la maison. | 1=Oui<br>2=Non<br>888=Ne sait pas<br>999 =Pas de réponse | |
| F4 | Ils ont raison de les frapper… si l'enfant ne veut pas aller à l'école | 1=Oui<br>2=Non<br>888=Ne sait pas<br>999 =Pas de réponse | |
| F5 | Ils ont raison de les frapper… si l'enfant ne veut pas aller au travail. | 1=Oui<br>2=Non<br>888=Ne sait pas<br>999 =Pas de réponse | |
| F6 | Ils ont raison de les frapper… si l'enfant ne s'occupe pas de ses frères ou de ses sœurs. | 1=Oui<br>2=Non<br>888=Ne sait pas<br>999 =Pas de réponse | |

| **Caregiver Survey (DRC)** | | | |
|---|---|---|---|
| **Question #** | **Question** | **Réponse options** | **Instructions** |
| F7 | Ils ont raison de les frapper… si l'enfant est engagé dans la prostitution par un adulte | 1=Oui<br>2=Non<br>888=Ne sait pas<br>999 =Pas de réponse | |
| F8 | Ils ont raison de les frapper… si l'enfant pisse au lit | 1=Oui<br>2=Non<br>888=Ne sait pas<br>999 =Pas de réponse | |
| F9 | Ils ont raison de les frapper… si l'enfant vole | 1=Oui<br>2=Non<br>888=Ne sait pas<br>999 =Pas de réponse | |
| F10 | Ils ont raison de les frapper… si l'enfant consomme de la drogue ou de l'alcool | 1=Oui<br>2=Non<br>888=Ne sait pas<br>999 =Pas de réponse | |
| F11 | Ils ont raison de les frapper… si l'enfant refuse de se marier | 1=Oui<br>2=Non<br>888=Ne sait pas<br>999 =Pas de réponse | |
| **J Prise de décision et Place de la femme** | | | |
| **J1** | Qui prend les décisions finale au sujet de l'utilisation de l'argent que vous gagnez ? | 1 = Principalement vous<br>2= Principalement votre [mari/compagnon]/[femme/compagne]<br>3= Votre [mari/compagnon]/[femme/compagne] et vous de manière égale<br>4= Principalement une autre personne du ménage<br>5= Non applicable si le ménage n'est pas concerné par ce sujet | |

| Caregiver Survey (DRC) | | | |
|---|---|---|---|
| **Question #** | **Question** | **Réponse options** | **Instructions** |
| **J2** | Qui prend les décisions finales au sujet de l'utilisation de l'argent que votre partenaire gagne ? | 1 = Principalement vous<br>2= Principalement votre [mari/compagnon]/[femme/compagne]<br>3= Votre [mari/compagnon]/[femme/compagne] et vous de manière égale<br>4= Principalement une autre personne du ménage<br>5= Non applicable si le ménage n'est pas concerné par ce sujet | |
| **J3** | Qui prend les décisions finales au sujet de vos propres soins de santé ? | 1 = Principalement vous<br>2= Principalement votre [mari/compagnon]/[femme/compagne]<br>3= Votre [mari/compagnon]/[femme/compagne] et vous de manière égale<br>4= Principalement une autre personne du ménage<br>5= Non applicable si le ménage n'est pas concerné par ce sujet | |
| **J4** | Qui prend les déicions finales au sujet des achats importants pour le ménage ? | 1 = Principalement vous<br>2= Principalement votre [mari/compagnon]/[femme/compagne]<br>3= Votre [mari/compagnon]/[femme/compagne] et vous de manière égale<br>4= Principalement une autre personne du ménage<br>5= Non applicable si le ménage n'est pas concerné par ce sujet | |

| Caregiver Survey (DRC) | | | |
|---|---|---|---|
| **Question #** | **Question** | **Réponse options** | **Instructions** |
| **J5** | Qui prend les déicions finales au sujet des depenses mineures (courses quotidiennes) pour le ménage ? | 1 = Principalement vous<br>2= Principalement votre [mari/compagnon]/[femme/compagne]<br>3= Votre [mari/compagnon]/[femme/compagne] et vous de manière égale<br>4= Principalement une autre personne du ménage<br>5= Non applicable si le ménage n'est pas concerné par ce sujet | |
| **J6** | Qui prend les décisions finales au sujet des visites à votre famille ou parents ? | 1 = Principalement vous<br>2= Principalement votre [mari/compagnon]/[femme/compagne]<br>3= Votre [mari/compagnon]/[femme/compagne] et vous de manière égale<br>4= Principalement une autre personne du ménage<br>5= Non applicable si le ménage n'est pas concerné par ce sujet | |
| **J7** | Qui prend les déicions finales au sujet des dépenses pour l'école ou les études des enfants ? | 1 = Principalement vous<br>2= Principalement votre [mari/compagnon]/[femme/compagne]<br>3= Votre [mari/compagnon]/[femme/compagne] et vous de manière égale<br>4= Principalement une autre personne du ménage<br>5= Non applicable si le ménage n'est pas concerné par ce sujet | |
| **J8** | Selon vous, est-il justifié qu'un mari frappe ou batte sa femme si elle sort sans le lui dire ? | 1 = Oui<br>2 = Non | |
| **J9** | Selon vous, est-il justifié qu'un mari frappe ou batte sa femme si elle néglige les enfants ? | 1 = Oui<br>2 = Non | |

| Caregiver Survey (DRC) | | | |
|---|---|---|---|
| **Question #** | **Question** | **Réponse options** | **Instructions** |
| **J10** | Selon vous, est-il justifié qu'un mari frappe ou batte sa femme si elle argumente avec lui ? | 1 = Oui<br>2 = Non | |
| **J11** | Selon vous, est-il justifié qu'un mari frappe ou batte sa femme si elle refuse d'avoir des rapports sexuels avec lui ? | 1 = Oui<br>2 = Non | |
| **J12** | Selon vous, est-il justifié qu'un mari frappe ou batte sa femme si elle brûle la nourriture ? | 1 = Oui<br>2 = Non | |
| **J13** | Selon vous, est-il justifié qu'un mari frappe ou batte sa femme si elle lui désobéit ? | 1 = Oui<br>2 = Non | |
| **J14** | Selon vous, est-il justifié qu'un mari frappe ou batte sa femme s'il sait qu'elle a été infidèle ? | 1 = Oui<br>2 = Non | |
| **J15** | Selon vous, est-il justifié qu'une femme refuse d'avoir des rapports sexuels avec son mari quand elle sait que son mari a une infection sexuellement transmissible ? | 1 = Oui<br>2 = Non | |
| **J16** | Selon vous, est-il justifié qu'une femme refuse d'avoir des rapports sexuels avec son mari quand elle sait que son mari a des rapports sexuels avec une autre femme ? | 1 = Oui<br>2 = Non | |
| **J17** | Selon vous, est-il justifié qu'une femme refuse d'avoir des rapports sexuels avec son mari quand elle vient d'accoucher ? | 1 = Oui<br>2 = Non | |
| **J18** | Selon vous, est-il justifié qu'une femme refuse d'avoir des rapports sexuels avec son mari quand elle est fatiguée ou pas d'humeur ? | 1 = Oui<br>2 = Non | |

| **Caregiver Survey (DRC)** | | | |
|---|---|---|---|
| **Question #** | **Question** | **Réponse options** | **Instructions** |
| **J19** | Selon vous, est-il justifié qu'une femme refuse d'avoir des rapports sexuels avec son mari quand il est ivre ? | 1 = Oui<br>2 = Non | |
| **J20** | Selon vous, est-il justifié qu'une femme refuse d'avoir des rapports sexuels avec son mari quand il la maltraite ? | 1 = Oui<br>2 = Non | |
| **J21** | Selon vous, est-il justifié qu'une femme refuse d'avoir des rapports sexuels avec son mari quand il refuse d'utiliser des préservatifs ? | 1 = Oui<br>2 = Non | |
| **G. Questions de Conclusion** | **Merci pour avoir répondu à ces questions. Je sais que quelques-unes d'entre elles avaient été difficiles. Souvenez-vous que personne dans votre communauté ne saura jamais ce que vous avez répondu.**<br><br>**Merci pour avoir répondu aux questions. Vous avez fait un bon travail.**<br><br>**Nous avons presque terminé. Avant de terminer notre discussion, j'ai quelques autres questions pour vous.** | | |
| G1 | Est-ce que les questions auxquelles vous avez déjà répondu étaient… | 1=Très facile à comprendre<br>2=Assez Facile à comprendre<br>3=Difficile à comprendre<br>4=Vraiment difficile à comprendre<br>888=Ne sait pas<br>999=Pas de réponse | |
| G2 | En général, avec quel niveau d'honnêteté diriez-vous que vous avez répondu à ces questions ? | 1=Pas du tout honnête<br>2=Pas vraiment honnête<br>3=Assez honnête<br>4=Vraiment honnête<br>5= Complètement honnête<br>888=Ne sait pas<br>999=Pas de réponse | |
| G3 | Y'a-t-il d'autres choses que vous voudriez ajouter ou des questions à me poser? | | |
| G4 | Avez-vous une quelconque réflexion ou une réaction à propos de cette interview ? | | |

| **Caregiver Survey (DRC)** | | | |
|---|---|---|---|
| **Question #** | **Question** | **Réponse options** | **Instructions** |
| | **(LE/LA REMERCIER POUR SA DISPONIBILITÉ. LUI ASSURER QUE SES RÉPONSES SONT CONFIDENTIELLES. L'INFORMER QU'IL/ELLE PEUT CONTACTER L'ÉQUIPE DE RECHERCHE N'IMPORTE QUAND POUR DES QUESTIONS ET DES PRÉOCCUPATIONS.)**<br><br>**Faite la transition a l'enquête des filles si c'est possible** | | |
| **H. Post Interview Enumérateur -only Questions** | **ENQUÊTEUR: MERCI DE RÉPONDRE AUX QUESTIONS SUIVANTES AVANT DE TERMINER L'INTERVIEW** | | |
| H1 | La personne semblait-elle comprendre les questions? | 1= Tout le temps<br>2<br>3= De temps en temps<br>4<br>5= Jamais | Choisir entre 1 et 5 |
| H2 | La personne semblait-elle répondre aux questions de façon aléatoire? | 1= Tout le temps<br>2<br>3= De temps en temps<br>4<br>5= Jamais | Choisir entre 1 et 5 |
| H3 | La personne semblait-elle réfléchir aux réponses avant de répondre ? | 1= Tout le temps<br>2<br>3= De temps en temps<br>4<br>5= Jamais | Choisir entre 1 et 5 |
| H4 | Y'avait-il quelqu'un d'autre présent au moment de l'interview? | 1=Oui<br>2=Non | Si "Oui", procéder à H4b and H4c<br>Si "Non", passer à H5 |
| H4b | Qui était cette autre personne? | _________ | Écrire seulement la relation |
| H4c | Jusqu'à quel niveau sentez-vous que la présence de cette personne influençait les réponses données par la personne? | 1= Beaucoup<br>2= Un peu<br>3= Très peu<br>4= Pas du tout | |
| H5 | L'interview a-t-elle été interrompue pour une quelconque raison? | 1=Oui<br>2=Non | Si "Oui", procéder à H5b and H5c<br>Si "Non", passer à H6 |
| H5b | Pourquoi l'interview a-t-elle été interrompue? | _______________ | |
| H5c | Selon vous, cela a-t-il affecté l'interview? | 1=Oui<br>2=Non | |

| Caregiver Survey (DRC) | | | |
|---|---|---|---|
| **Question #** | **Question** | **Réponse options** | **Instructions** |
| H6 | Quel était le contexte dans lequel l'interview a eu lieu? | 1= Calme, privé<br>2= Quelque bruit, presque privé<br>3= Trop de bruit, des gens tout autour | |
| H7 | Comment évalueriez-vous la capacité de la personne de comprendre la plupart des questions? | 1= N'a pas beaucoup compris<br>2= A compris un peu<br>3= A compris modérément<br>4= A beaucoup compris<br>5= A compris énormément | |
| H8 | Quelles questions ont semblé plus difficiles à comprendre pour la personne? | | S'il vous plait, lister les questions |
| H9 | En général, comment évaluerez-vous l'intérêt que la personne portait à l'interview? | 1= Vraiment haut<br>2= Au dessus de la moyenne<br>3= Moyenne<br>4= En dessous de la moyenne<br>5= Vraiment bas | |
| H10 | La personne a-t-elle besoin d'une référence à un prestataire de services quelconque? | ____________________________<br>____________________________<br>____________________________<br>____________________________<br>____________________________<br>_____ | Si oui, décrire s'il vous plait. |
| | **FIN DU QUESTIONNAIRE, TRÈS BON TRAVAIL!** | | |
