# Supplementary material for: Gender-equitable caregiver attitudes and education and safety of adolescent girls in South Kivu, DRC: A secondary analysis from a randomized controlled trial
Source: PLoS Med. 2021 Sep 28;18(9):e1003619. doi: 10.1371/journal.pmed.1003619 (PMC8478225; doi:10.1371/journal.pmed.1003619)
Supplement: S5 Questionnaire — (PDF) [file pmed.1003619.s008.pdf]

Caregivers’ survey- Mashi

QUANTITATIF ADULTES /MASHI

**Amadoso g’ababusi**			
**Q#**	**Amadoso**	**Mashuzo**	**Instructions**
**A. Questions administratives**			
A1	Izino ly’ecishagala/lugo	_____ _____ _____	
A2	Cishagala/karatye	_____ _____ _____	
A3	Cishagala cisungunu (murhundu)	_____ _____ _____	
A4	Lulimi	-Mashi<br>-Swahili	
A5	Ecimanyiso c’omushonderezi	------------------------------------	
A6	Olusiku lw’ene	__<br>_________/_________/___________	jj/mm/aaaa
A7	Kasanzi : Nsaa zokurhangira	___________: ___________	24 heures
A8	Kasanzi : Nsaa zokuyusa	___________: ___________	24 heures
A9	Ecimanyiso c’omubusi	______________________	
A10	Ecimanyiso c’omunyere/o’mwananyere	______________________	
A11	Emyaka y’omubusi/omwimangizi		888=Ne sait pas

Caregivers' survey- Mashi

**Amadoso g'ababusi**			
**Q#**	**Amadoso**	**Mashuzo**	**Instructions**
**B**	**Rwamacibi lamusa,namana sima okukubona.Koko okubona wayemire lulya lundi lusiku okurhwabuganana mwezi nshambalo. Nakasimire okubakengeza ebi rhashambalagako omango rhwazindigi shambala..Rhwabadosa amadoso gabayerekire n'oku olama na mwali wawe rhwayandikaga muli ogu mukolo,n'entanya zawe kuli bintu biguma na biguma. Chiru ankaba oli we,oli nwali wawe,ntaye wahabwa akantu koshi koshi,muli ezi nshambalo,ciru nta lusaranga erhi kandi kantu, Ebi warhubwira byarhabala oku jira bwinja emikolo haguma nabanyere bomu CONGO, rhunacikebirwe oku wagerereza n'okushuza okubinali n'obushinganyanya oku madoso.**<br><br>**Orhasezibwi oku rhuganiza ene. Wanaderha oku orhasimiri okuganira nanirhu ene, birhanarhugayise. Kandi wanalahira okushuza oku ngasi idoso orhaciyunvirhi ko bwinja erhi eli orhalonzizi, na nta kantu kabi kakuhikira erhi kahikire mwali wawe oyandisirwe muli ogu mukolo; Rhwanagendekera oku idoso likulikire.**<br><br>**Byoshi warhubwira bya kolesibwa oku busagasi bonene,na ntaye yeshiyeshi(cirhu na mwali wawe,omulala gwawe,abira erhi undi muntu w'omulugo) wanamanye bici warhubwizire. Byoshi warhubwira byabikwa ihwe.**<br><br>**Embere rhurhangire nankasimire ombwire erhi ochiyunvirhe bwinja, nakandi erhi wayunvirhe kushimbana na byoshi rhwaganira ko.Kogwerhe amadoso ?koyemire okunganiza ene ?**<br><br>**Kwinja.Rhwarhondera n'amadoso malembu,kushimbana nebi kuyerekire. Erhi rwakaderha « Mwali wawe » rhwalonza okuderha omunyere rhwayandisire muli ogu mukolo,olya oli mubusi erhi mwimangizi wage.**		
B1	Ogwerhe miaka inga?	_____________ Ans<br><br>888=Ntamanyiri<br><br>999 = Ntishuzo	Nkarhayenezizi myaka 18 orhagendekeraga

Caregivers' survey- Mashi

**Amadoso g'ababusi**			
**Q#**	**Amadoso**	**Mashuzo**	**Instructions**
B4	Muntu ndi orhabala okubiyerekire olufaranga omunyumpa mwali wawe alama ?	1= Nyama,<br>2= Larha,<br>3= Shakulwe erhi mugaka,<br>4= Larhazala erhi nyamazala,<br>5= Mushinja wani/ mukulu wani,<br>6=Shenge/ nyamalume,<br>7= Windi womumulala,<br>8= Rhurhayimbanwa oli warhubuka,<br>9= wundi<br>888= Ntamanyiri<br>999= Ntishuzo	Cocher tout ce qui convient.<br><br>Si "Responsable qui n'est pas membre de la famille" ou "Autre", préciser
C. **Ebiyerekire abanyere n'abarhabana**	**Nalonza oku kudosa mwakakasanzi amadoso maguma maguma enyanya lyebi omanyire oku banyere n'abarhabana. Abantu barhayunva kuguma kuli ebi bintu, Nta mashuzo minja erhi mabi.Omanye oku amashuzo gawe garhasambisibwe,na kandi wanalahira okushuza oku idoso ngasi mango orhalonzizi,buzira kadali kuli we erhi kuli mwali wawe.**		
C1	Biri binja bwenene abana barhabana baj'emasomo kulusha abanyere	1 = Neci,<br>2 = Nanga<br>888= Ntamanyiri<br>999= Ntishuzo	
C2	Abanyere baligwasirwe barhumwe emasomo akaba ntabyo bajir'eka.	1 = Neci,<br>2 = Nanga<br>888= Ntamanyiri<br>999= Ntishuzo	
C3	Echirhumire bwenene abarhabana baj' emasomo kulusha abanyere bulya abarhabana bana lera bwinja ababusi babo amango bakola bashosi	1 = Neci,<br>2 = Nanga<br>888= Ntamanyiri<br>999= Ntishuzo	
C4	Akaba hali enfaranga nsungunu z'okusomesa abana, ezo nfaranga zigwasirwe zihabwe abanarhabana bonene.	1 = Neci,<br>2 = Nanga<br>888= Ntamanyiri<br>999= Ntishuzo	
C5	Abakazi baligwasirwe balekere abalume okurhambula ecihugo/ kujir'epolitiki.	1 = Neci,<br>2 = Nanga<br>888= Ntamanyiri<br>999= Ntishuzo	

Caregivers' survey- Mashi

Amadoso g'ababusi			
**Q#**	**Amadoso**	**Mashuzo**	**Instructions**
C6	Omukazi akwanine agwarhe iba erhi omugala muguma erhi windi mushamuka waka mulanga bulya arhakacilanga yene.	1 = Neci,<br>2 = Nanga<br>888= Ntamanyiri<br>999= Ntishuzo	
C7	Omuntu erhi abantu bonene omukazi akacikubagira amango akola mugikulu anali omugala erhi abagala.	1 = Neci,<br>2 = Nanga<br>888= Ntamanyiri<br>999= Ntishuzo	
C8	Omukazi mwinja arhalahira ebi iba adesire, ciru akaba amanyire oku ebi iba adesire birhali byokuli.	1 = Neci,<br>2 = Nanga<br>888= Ntamanyiri<br>999= Ntishuzo	
C9	Abanyere baligwasire bakakola embuga erhi kuli n'emwabo nka barhabana.	1 = Neci,<br>2 = Nanga<br>888= Ntamanyiri<br>999= Ntishuzo	
C10	Nana sima mwali wani apate akazi aharhali ahaka lyo achilera, nanka biankahashikana alere n'omulala gwage.	1 = Neci,<br>2 = Nanga<br>888= Ntamanyiri<br>999= Ntishuzo	
**D. Obulezi bwa abana**			
D1	Kamurhanyize oku okulera bwinja abanyere mugwasirwe mukabashurha ?	1= Nechi<br>2= Nanga,<br>888= Ntamanyiri<br>999= Ntishuzo	
D2	Kuhika oku ngazi nchi olwo luderho kwobinali ?<br><br>Biri byabulagirire okumanya aha mwali w'omuntu ali ngasi mango.	1= Kwobinali bwenene<br>2= Kwobinali hitya<br>3= Birhali ntyo<br>888= Ntamanyiri<br>999= Ntishuzo	
	**Buno nkolaga n'akudosa amadoso maguma maguma oku biyerekire oku oliwarhanyiza mwali wawe omu nsiku zayisha. Okengere oku,wanaderha oku orhamanyiri,irhi olahire oku orhashuza oku idoso amango arhalonzizi, Koyunvirhe ?**		

Caregivers' survey- Mashi

**Amadoso g'ababusi**			
**Q#**	**Amadoso**	**Mashuzo**	**Instructions**
D3	Ka mwali wawe ali asoma muli gano mango ?muli ezi nsiku ?	1=Nechi<br>2=Nanga<br>888= Ntamanyiri<br>999= Ntishuzo	
D4	Omu isimo lya kanga orhanyize mwali wawe agwasirwe ahikire embere aleke amasomo?<br>PASSER CETTE QUESTION SI LA FILLE N'EST PAS A L'ÉCOLE.	___________(Isomo lya kanga)<br>888= Ntamanyiri<br>999= Ntishuzo	
D5	Kuhik'oku miaka inga wakalonza mwali wawe abe aciri emasomo ?<br>PASSER CETTE QUESTION SI LA FILLE N'EST PAS A L'ÉCOLE.	____________(miaka)	Écrire 88 si "Ne sait pas"<br>Écrire 99 si "Pas de réponse"
D6	Okumyaka inga wakolonza mwali wawe aherukire?	___________(miaka)	Écrire 88 si "Ne sait pas"<br>Écrire 99 si "Pas de réponse"
D7	Okumiaka inga wakalonza mwali wawe aburhe omwana murhanzi ?	___________(miaka)	Écrire 88 si "Ne sait pas"<br>Écrire 99 si "Pas de réponse"
D8	Erhi omunyere akayishi kubwira oku bamushozire arhanasimiri/ bamugwarha n'emisi, kawana mubwira buzira nshonyi eniana ly'ebigamba bihali byakamurhabala ?	1=Nechi<br>2=Nanga<br>888=Ntamanyiri<br>999= Ntaishuzo	Si "Non" ou « Ne sait pas », passer à D8b

Caregivers' survey- Mashi

Amadoso g'ababusi			
**Q#**	**Amadoso**	**Mashuzo**	**Instructions**
D8a	Akaba neci, bigamba, bihi na bihi ?	1= Emwamunganga<br>2= Emwabazuzi/Emwa leta<br>3= Abayunvirhiza,kubuka mawazo<br>4=Ebigamba by'oburhidisi<br>5=Ebigamba by'abakazi OCB (bikayunvirhiza ababo, etc.)<br>6=Abandi<br>888=Ntamanyiri<br>999= Ntishuzo	Passer à E1
D8b	Akaba nanga, bulyagurhi ?	1= Ntayishi aha bankamurhabala<br>2= Ntaho ankarhabalirwa<br>3= Birhanyerekiri,gurhali mukologwani<br>4= Oburhabale bwabo burhakolwa bwinja<br>5=Ebigamba bahaba ci birhahikwa ko na ngasi ye (kubula enfranga,biba kuli n'olugo, etc.)<br>6=Ebindi<br>888=Ntamanyiri<br>999= Ntishuzo	
**E. Ebijiro b'yomubusi**	**Kawakabona n'okuyunva oku ababusi na abalezi bajira abana babo mango maguma maguma. Waliha olondole, erhi ebi bidesirwe « biri hofi ngasi mango kwobinali », « hali amango byankaba kwobinali », « birhalugiba kwobinali », erhi « hali nkahofi birhaba kwobinali ». Wanaderha okurhamanyiri,irhi olahire oku orhashuzaoku idoso amango arhalonzizi,orhanabe gurhi.**		
E1	Nanka derha ebintu ebinja oku mwana wani	1=Biri hofi ngasimango kwobinali<br>2=Hali amango byakaba kwobinali<br>3=Birhalugiba kwobinali<br>4=Hali nkahofi birhaba kwobinali<br>888= Ntamanyiri<br>999= Ntishuzo	
E2	Ntashibirira omwana wani	1=Biri hofi ngasimango kwobinali<br>2=Hali amango byakaba kwobinali<br>3=Birhalugiba kwobinali<br>4=Hali nkahofi birhaba kwobinali<br>888= Ntamanyiri<br>999= Ntishuzo	
E3	Nakacijira mulembu oku mwana wani lyo acinkubagira/abwira ebimuyerekire	1=Biri hofi ngasimango kwobinali<br>2=Hali amango byakaba kwobinali<br>3=Birhalugiba kwobinali<br>4=Hali nkahofi birhaba kwobinali<br>888= Ntamanyiri<br>999= Ntishuzo	

Caregivers' survey- Mashi

**Amadoso g'ababusi**			
**Q#**	**Amadoso**	**Mashuzo**	**Instructions**
E4	Hali amango nshura omwana wani arhanali wakushurwa	1=Biri hofi ngasimango kwobinali<br>2=Hali amango byakaba kwobinali<br>3=Birhalugiba kwobinali<br>4=Hali nkahofi birhaba kwobinali<br>888= Ntamanyiri<br>999= Ntishuzo	
E5	Nanka bona omwana wani nkali wakumbera irhwe	1=Biri hofi ngasimango kwobinali<br>2=Hali amango byakaba kwobinali<br>3=Birhalugiba kwobinali<br>4=Hali nkahofi birhaba kwobinali<br>888= Ntamanyiri<br>999= Ntishuzo	
E6	Nampane omwana wani nkandi burhe	1=Biri hofi ngasimango kwobinali<br>2=Hali amango byakaba kwobinali<br>3=Birhalugiba kwobinali<br>4=Hali nkahofi birhaba kwobinali<br>888= Ntamanyiri<br>999= Ntishuzo	
E7	Nankaba ngwerhe binji bwakujira, na ntakasanzi ko kushuza oku madoso go mwana wani	1=Biri hofi ngasimango kwobinali<br>2=Hali amango byakaba kwobinali<br>3=Birhalugiba kwobinali<br>4=Hali nkahofi birhaba kwobinali<br>888= Ntamanyiri<br>999= Ntishuzo	
E8	Ngwerhe akagayo oku mwana wani	1=Biri hofi ngasimango kwobinali<br>2=Hali amango byakaba kwobinali<br>3=Birhalugiba kwobinali<br>4=Hali nkahofi birhaba kwobinali<br>888= Ntamanyiri<br>999= Ntishuzo	
E9	Nanka yumva simisibwe bwenene n'ebi omwana wani ajira	1=Biri hofi ngasimango kwobinali<br>2=Hali amango byakaba kwobinali<br>3=Birhalugiba kwobinali<br>4=Hali nkahofi birhaba kwobinali<br>888= Ntamanyiri<br>999= Ntishuzo	
E10	Nanderhe bintu binji birhali binja oku mwana wani	1=Biri hofi ngasimango kwobinali<br>2=Hali amango byakaba kwobinali<br>3=Birhalugiba kwobinali<br>4=Hali nkahofi birhaba kwobinali<br>888= Ntamanyiri<br>999= Ntishuzo	

Caregivers' survey- Mashi

**Amadoso g'ababusi**			
**Q#**	**Amadoso**	**Mashuzo**	**Instructions**
E11	Ntahangaika nka omwana wani ampuna oburhabale (ntamushibirira)	1=Biri hofi ngasimango kwobinali<br>2=Hali amango byakaba kwobinali<br>3=Birhalugiba kwobinali<br>4=Hali nkahofi birhaba kwobinali<br>888= Ntamanyiri<br>999= Ntishuzo	
E12	Nankalonza manye erhi omwana wani aciyunvirhe okualonzibwe anali wa bulangirire	1=Biri hofi ngasimango kwobinali<br>2=Hali amango byakaba kwobinali<br>3=Birhalugiba kwobinali<br>4=Hali nkahofi birhaba kwobinali<br>888= Ntamanyiri<br>999= Ntishuzo	
E13	Nankashibirira bwenene omwana wani.	1=Biri hofi ngasimango kwobinali<br>2=Hali amango byakaba kwobinali<br>3=Birhalugiba kwobinali<br>4=Hali nkahofi birhaba kwobinali<br>888= Ntamanyiri<br>999= Ntishuzo	
E14	Nankagayisa omwana wani	1=Biri hofi ngasimango kwobinali<br>2=Hali amango byakaba kwobinali<br>3=Birhalugiba kwobinali<br>4=Hali nkahofi birhaba kwobinali<br>888= Ntamanyiri<br>999= Ntishuzo	
E15	Omwana akangayisa, namuyunvise okwali mulikirire	1=Biri hofi ngasimango kwobinali<br>2=Hali amango byakaba kwobinali<br>3=Birhalugiba kwobinali<br>4=Hali nkahofi birhaba kwobinali<br>888= Ntamanyiri<br>999= Ntishuzo	
E16	Nankayibagira ebintu byo bulangirire birya omwana wani akarhanya okunkwanine mbi kengere	1=Biri hofi ngasimango kwobinali<br>2=Hali amango byakaba kwobinali<br>3=Birhalugiba kwobinali<br>4=Hali nkahofi birhaba kwobinali<br>888= Ntamanyiri<br>999= Ntishuzo	
E17	Nankajira kuhika omwana wani aciyunve oku ebi aliajira biri byabulagirire	1=Biri hofi ngasimango kwobinali<br>2=Hali amango byakaba kwobinali<br>3=Birhalugiba kwobinali<br>4=Hali nkahofi birhaba kwobinali<br>888= Ntamanyiri<br>999= Ntishuzo	

Caregivers' survey- Mashi

**Amadoso g'ababusi**			
**Q#**	**Amadoso**	**Mashuzo**	**Instructions**
E18	Erhi omwana wani akajira ecijiro cibi namukalihire, n'okumuyobohya	1=Biri hofi ngasimango kwobinali<br>2=Hali amango byakaba kwobinali<br>3=Birhalugiba kwobinali<br>4=Hali nkahofi birhaba kwobinali<br>888= Ntamanyiri<br>999= Ntishuzo	
E19	Nankayunva entanya z'omwana wani noku muha emisi yo kuziderha	1=Biri hofi ngasimango kwobinali<br>2=Hali amango byakaba kwobinali<br>3=Birhalugiba kwobinali<br>4=Hali nkahofi birhaba kwobinali<br>888= Ntamanyiri<br>999= Ntishuzo	
E20	Nankayunva nka abandi bana bobinja kulusha owani	1=Biri hofi ngasimango kwobinali<br>2=Hali amango byakaba kwobinali<br>3=Birhalugiba kwobinali<br>4=Hali nkahofi birhaba kwobinali<br>888= Ntamanyiri<br>999= Ntishuzo	
E21	Nankayereka /kuyunvisa omwana wani oku ntamulonzizi	1=Biri hofi ngasimango kwobinali<br>2=Hali amango byakaba kwobinali<br>3=Birhalugiba kwobinali<br>4=Hali nkahofi birhaba kwobinali<br>888= Ntamanyiri<br>999= Ntishuzo	
E22	Nankayereka/kuyunvisa omwana wani oku musima	1=Biri hofi ngasimango kwobinali<br>2=Hali amango byakaba kwobinali<br>3=Birhalugiba kwobinali<br>4=Hali nkahofi birhaba kwobinali<br>888= Ntamanyiri<br>999= Ntishuzo	
E23	Ntayunvirhiza/ntashibirira omwana nka kuno yene arha mbezire irhwe	1=Biri hofi ngasimango kwobinali<br>2=Hali amango byakaba kwobinali<br>3=Birhalugiba kwobinali<br>4=Hali nkahofi birhaba kwobinali<br>888= Ntamanyiri<br>999= Ntishuzo	
E24	Nanka lera omwana wani n'obushagaluke	1=Biri hofi ngasimango kwobinali<br>2=Hali amango byakaba kwobinali<br>3=Birhalugiba kwobinali<br>4=Hali nkahofi birhaba kwobinali<br>888= Ntamanyiri<br>999= Ntishuzo	
**F. Attitudes à propos de la discipline .Kushimbana n'obuhane.**	**Hali amango ababusi erhi omuntu olera abana aba burhe erhi ebi abana balibajira byo birhuma, banabashurhe bwenene.Abantu barhayunva kuguma oku babusi bashurha abana Okubwawe mangachi ababusi bayemerirwe okushurha abana babo ?**		
F1	Bagwasirwe okushurha abana…Nka omwana arhayunva	1 = Neci<br>2 = Nanga<br>888= Ntamanyiri<br>999= Ntishuzo	

Caregivers' survey- Mashi

**Amadoso g'ababusi**			
**Q#**	**Amadoso**	**Mashuzo**	**Instructions**
F2	Bagwasirwe okushurha abana…Nka omwana arhayemiri ebi ababusi badesire	1 = Neci<br>2 = Nanga<br>888= Ntamanyiri<br>999= Ntishuzo	
F3	Bagwasirwe okushurha abana…Nka omwana akayaka ahaka	1 = Neci<br>2 = Nanga<br>888= Ntamanyiri<br>999= Ntishuzo	
F4	Bagwasirwe okushurha abana…Nka omwana arhalonzizi okuja emasomo	1 = Neci<br>2 = Nanga<br>888= Ntamanyiri<br>999= Ntishuzo	
F5	Bagwasirwe okushurha abana…Nka omwana arhalonza kuchikola	1 = Neci1<br>2 = Nanga<br>888= Ntamanyiri<br>999= Ntishuzo	
F 6	Bagwasirwe okushurha abana… Nka omwana arhaleziri bwinja bashinja bage erhi bali babo.	1 = Neci<br>2 = Nanga<br>888= Ntamanyiri<br>999= Ntishuzo	
F7	Bagwasirwe okushurha abana… Nka omwana ali ajira obugonyi bon'omuntu mukulu.	1 = Neci<br>2 = Nanga<br>888= Ntamanyiri<br>999= Ntishuzo	
F8	Bagwasirwe okushurha abana… Nka omwana anakashubala oku nchingo	1 = Neci<br>2 = Nanga<br>888= Ntamanyiri<br>999= Ntishuzo	
F9	Bagwasirwe okushurha abana… Nka omwana anakazimba	1 = Neci<br>2 = Nanga<br>888= Ntamanyiri<br>999= Ntishuzo	
F10	Bagwasirwe okushurha abana… Nka omwana anakanywa ecimogi erhi manvu	1 = Neci<br>2 = Nanga<br>888= Ntamanyiri<br>999= Ntishuzo	
F11	Bagwasirwe okushurha abana… Nka omwana akalahira okuheruka/okuja omubuhya	1 = Neci<br>2 = Nanga<br>888= Ntamanyiri<br>999= Ntishuzo	
**J. Decision-making and gender norms**			

Caregivers' survey- Mashi

**Amadoso g'ababusi**			
**Q#**	**Amadoso**	**Mashuzo**	**Instructions**
**J1**	Indi oyanka emihigo mizinda omukukolesa olugeshe okamarhaga ?	1 = Bwenene we / Wene ne<br>2 = Bwenene balo/ mulume mukawe/ muirakazi olama naye<br>3 = Balo/Mwiralume wawe na nawe kuguma<br>4 = Kanji kanji wundi muntu w'omu mulala<br>5 = Birhashinganini	
**J2**	Indi oyanka emihigo mizinda okukolesibwa k'olugeshe balo (mwira) wawe akamarhaga ?	1 = Bwenene we / Wene ne<br>2 = Bwenene balo/ mulume mukawe/ muirakazi olama naye<br>3 = Balo/Mwiralume wawe na nawe kuguma<br>4 = Kanji kanji wundi muntu w'omu mulala<br>5 = Birhashinganini	
**J3**	Indi oyanka emihigo mizinda omubiyerekire okubukwa kwawe	1 = Bwenene we / Wene ne<br>2 = Bwenene balo/ mulume mukawe/ muirakazi olama naye<br>3 = Balo/Mwiralume wawe na nawe kuguma<br>4 = Kanji kanji wundi muntu w'omu mulala<br>5 = Birhashinganini	
**J4**	Indi oyanka emihigo mizinda omubiyerekire eby'okugula b'yobulagirire ecambu b'yomumulala gwawe ?	1 = Bwenene we / Wene ne<br>2 = Bwenene balo/ mulume mukawe/ muirakazi olama naye<br>3 = Balo/Mwiralume wawe na nawe kuguma<br>4 = Kanji kanji wundi muntu w'omu mulala<br>5 = Birhashinganini	
**J5**	Indi oyanka emihigo mizinda omubiyerekire okuheza olugeshe lusungunu (okuj'eyi n'eyi) y'omulala?	1 = Bwenene we / Wene ne<br>2 = Bwenene balo/ mulume mukawe/ muirakazi olama naye<br>3 = Balo/Mwiralume wawe na nawe kuguma<br>4 = Kanji kanji wundi muntu w'omu mulala<br>5 = Birhashinganini	

Caregivers' survey- Mashi

**Amadoso g'ababusi**			
**Q#**	**Amadoso**	**Mashuzo**	**Instructions**
**J6**	Indi oyanka emihigo mizinda omubiyerekire okulambagirira omulala gwawe erhi ababusi ?	1 = Bwenene we / Wene ne<br>2 = Bwenene balo/ mulume mukawe/ muirakazi olama naye<br>3 = Balo/Mwiralume wawe na nawe kuguma<br>4 = Kanji kanji wundi muntu w'omu mulala<br>5 = Birhashinganini	
**J7**	Indi oyanka emihigo mizinda omubiyerekire'amaso mo erhi okusomesa abana ?	1 = Bwenene we / Wene ne<br>2 = Bwenene balo/ mulume mukawe/ muirakazi olama naye<br>3 = Balo/Mwiralume wawe na nawe kuguma<br>4 = Kanji kanji wundi muntu w'omu mulala<br>5 = Birhashinganini	
**J8**	Oku bwawe, ka biyunvikine, omulume ashurhe mukage bulya arhengire ahambuga buzira kumubwira ?	1 = Neci<br>2 = Nanga	
**J9**	Oku bwawe, ka biyunvikine, omulume ashurhe mukage bulya arhalembiri abana bwinja ?	1 = Neci<br>2 = Nanga	
**J10**	Oku bwawe, ka biyunvikine omulume ashurhe mukage bulya aly'azozanya naye ?	1 = Neci<br>2 = Nanga	
**J11**	Oku bwawe, ka biyunvikine, omulume ashurhe mukage bulya alahire okujira ecijiro c'obuhya naye ?	1 = Neci<br>2 = Nanga	

Caregivers' survey- Mashi

Amadoso g'ababusi			
**Q#**	**Amadoso**	**Mashuzo**	**Instructions**
**J12**	Oku bwawe, ka biyunvikine, omulume ashurhe mukage bulya amasiriza ebiryo ?	1 = Neci<br>2 = Nanga	
**J13**	Oku bwawe ka biyunvikine, omulume ashurhe mukage bulya aramuyumva ?	1 = Neci<br>2 = Nanga	
**J14**	Oku bwawe ka biyunvikine, omulume ashurhe mukage bulya ali wabalume banji ?	1 = Neci<br>2 = Nanga	
**J15**	Oku bwawe ka biyunvikine omukazi alahire okujira ecijiro c'obuhya n'iba bulya aherhe enkumbi ?	1 = Neci<br>2 = Nanga	
**J16**	Oku bwawe ka biyunvikine omukazi alahire okujira ecijiro c'obuhya n'iba bulya amanyire okw'iba aliajira ecijiro c'obuhya n'owindi mukazi ?	1 = Neci<br>2 = Nanga	
**J17**	Oku bwawe ka biyunvikine omukazi alahire okujira ecijiro c'obuhya n'iba amango arhengi burha ?	1 = Neci<br>2 = Nanga	
**J18**	Oku bwawe ka biyunvikine omukazi alahire okujira ecijiro c'obuhya n'iba amango arhamire erhi arhaciyunvirhi ?	1 = Neci<br>2 = Nanga	

Caregivers' survey- Mashi

**Amadoso g'ababusi**			
**Q#**	**Amadoso**	**Mashuzo**	**Instructions**
**J19**	Oku bwawe kabiyunvikine omukazi alahire okujira ecijiro c'obuhya n'iba amango iba alalusire ?	1 = Neci<br>2 = Nanga	
**J20**	Oku bwawe ka biyunvikine omukazi alahire okujira ecijiro c'obuhya n'iba amango amurhindibuzize ?	1 = Neci<br>2 = Nanga	
**J21**	Oku bwawe ka biyunvikine omukazi alahire okujira ecijiro c'obuhya n'iba amango alahire okuyambala ecipira (kapoti) ?	1 = Neci<br>2 = Nanga	
**G. Amadoso gokufundika**	**Koko oku kushuza kwaga madoso. Manyire oku hali maguma na maguma gali mazibu muli go.Okengere oku ntaye omu logo lwawe onamanye ebi washuzize.<br>Koko oku kushuza oku madoso,wakozire omukolo mwinja<br>Rhukola ebwakuyusa,embere rhuyuse ensambalo zirhu,ngwerhe gandi madoso maguma kuli we.**		
G1	Ka amadoso washuzize ko gali…	1=lilembu bwenene okuyunva<br>2=lilembu okuyunva<br>3=lizibu okuyunva<br>4=neci lizibu bwenene okuyunva<br>888=Ntamanyiri<br>999= Ntaishuzo	
G2	Kulusha byoshi,okuli kwawe ngahi kwali hikire ene okashuza kuli ago madoso ?	1=arhali okuli bwenene<br>2=nanga nta okuli<br>3=neci okuli hitya<br>4=neci okuli<br>5= okuli loshi loshi<br>888=Ntamanyiri<br>999= Ntaishuzo	
G3	kahali ebindi bintu wakalonzize okuyushula erhi madoso ga ku ndosa ?		

Caregivers' survey- Mashi

**Amadoso g'ababusi**			
**Q#**	**Amadoso**	**Mashuzo**	**Instructions**
G4	Ka ogwerhe iwazo liguma na liguma erhi kundi wadosa kuli ezi nshambalo(madoso) ?		
	**Omuvuge omunkwa oku kasanzi. Omuyunvise oku amashuzo gage gabera omu ihwe. Omubwire oku analonza abasagasi mamango goshi alonzize nka agwerhe amadoso erhi bindi kushimbana n'obusagasi**<br><br>**Faite la transition a l'enquête des filles si c'est possible :**		
**H. Post Interview Enumérateur-only Questions**	**ENQUÊTEUR: MERCI DE RÉPONDRE AUX QUESTIONS SUIVANTES AVANT DE TERMINER L'INTERVIEW**		
H1	La personne semblait-elle comprendre les questions?	1= Tout le temps<br>2<br>3= De temps en temps<br>4<br>5= Jamais	Choisir entre 1 et 5
H2	La personne semblait-elle répondre aux questions de façon aléatoire?	1= Tout le temps<br>2<br>3= De temps en temps<br>4<br>5= Jamais	Choisir entre 1 et 5
H3	La personne semblait-elle réfléchir aux réponses avant de répondre ?	1= Tout le temps<br>2<br>3= De temps en temps<br>4<br>5= Jamais	Choisir entre 1 et 5
H4	Y'avait-il quelqu'un d'autre présent au moment de l'interview?	1=Oui<br>2=Non	Si "Oui", procéder à H4b and H4c<br>Si "Non", passer à H5
H4b	Qui était cette autre personne?	_________	Écrire seulement la relation
H4c	Jusqu'à quel niveau sentez-vous que la présence de cette personne influençait les réponses données par la personne?	1= Beaucoup<br>2= Un peu<br>3= Très peu<br>4= Pas du tout	
H5	L'interview a-t-elle été interrompue pour une quelconque raison?	1=Oui<br><br>2=Non	Si "Oui", procéder à H5b and H5c<br>Si "Non", passer à H6
H5b	Pourquoi l'interview a-t-elle été interrompue?	_______________	
H5c	Selon vous, cela a-t-il affecté l'interview?	1=Oui<br><br>2=Non	
H6	Quel était le contexte dans lequel l'interview a eu lieu?	1= Calme, privé<br>2= Quelque bruit, presque privé<br>3= Trop de bruit, des gens tout autour	

Caregivers' survey- Mashi

**Amadoso g'ababusi**			
**Q#**	**Amadoso**	**Mashuzo**	**Instructions**
H7	Comment évalueriez-vous la capacité de la personne de comprendre la plupart des questions?	1= N'a pas beaucoup compris<br>2= A compris un peu<br>3= A compris modérément<br>4= A beaucoup compris<br>5= A compris énormément	
H8	Quelles questions ont semblé plus difficiles à comprendre pour la personne?		S'il vous plait, lister les questions
H9	En général, comment évaluerez-vous l'intérêt que la personne portait à l'interview?	1= Vraiment haut<br>2= Au dessus de la moyenne<br>3= Moyenne<br>4= En dessous de la moyenne<br>5= Vraiment bas	
H10	La personne a-t-elle besoin d'une référence à un prestataire de services quelconque?	________________________________<br>________________________________<br>________________________________<br>_______________	Si oui, décrire s'il vous plait.
	**FIN DU QUESTIONNAIRE, TRÈS BON TRAVAIL!**		
