# Supplementary material for: Gender-equitable caregiver attitudes and education and safety of adolescent girls in South Kivu, DRC: A secondary analysis from a randomized controlled trial
Source: PLoS Med. 2021 Sep 28;18(9):e1003619. doi: 10.1371/journal.pmed.1003619 (PMC8478225; doi:10.1371/journal.pmed.1003619)
Supplement: S6 Questionnaire — (PDF) [file pmed.1003619.s009.pdf]

Caregivers' survey- Swahili

QUANTITATIF ADULTES /SWAHILI

Caregiver Survey (DRC)			
**Question #**	**Question**	**Réponse options**	**Instructions**
**A. Questions administratives**			
A1	Jina la kijiji/sehemu	____________________________ ____________	Record
A2	Kata/Kartier	_____ _____ _____	Use codes given by listing
A3	Kijiji ndogo la Kata	_____ _____ _____	Use codes given by listing
A4	Luga	1=Swahili<br>2=Mashi	
A5	Kionyesho cha mtafiti	_____ _____	
A6	Tarehe ya siku ya leo	___ __ ___/_________/________	jj/mm/aaaa
A7	Saa ya mwanzo ya utafiti	____________: ____________	24 heures
A8	Saa ya mwisho ya utafiti	____________: ____________	24 heures
A9	Kitambulisho cha mzazi	_______________________	
A10	Kitambulisho cha binti/msika	_______________________	
A11	Myaka ya muzazi wala musimamizi wa binti		888=Ne sait pas
**B. Informations Démographiques**	**Jambo tena, nina furaha kuwa ona tena. Aksanti kwaku itika ile siku kukutana kwa iyi mazungumuzo. Nilitaka wakumbusha mambo tuliongeya siku zilizo pita. Tuta wa uliza ma ulizo Fulani juya nyinyi wenyewe, najuya usiano nawa binti wenu, wenye kwa iyo programme, tena kwa maoni yenu juya mada fulani. Atakama ata wewe, ata binti wako muta fahidika tu kwa kujiunga kwa iyi kiulizo, na hamuta faidika kamwe napesa wala vitu mbali mbaki, tuta kusanya izo jibu zote juyaku badirisha hali zawa binti mu inshi yetu ya Congo, natuna matumaini kama muta waza muzuri nakutu swali kwa ukweli baada yaku jibu.**<br><br>**Akuna kitu kinawa kaza wala kuwa sukuma kwaku jibu leo. Munaeza tuambia kama hamutaki sema leo, na hatuta kasirika kamwe. Mutaeza ata katala kutu jibu kwa ulizo yoyote yenye hamusikie kujibia kama inawa tia kwenye wasiwasi, na akuna kitu itaba fikia wala kitu ita fikia binti wako mwenye kua kwenye iyi programme; natuta pita kwa taratibu kwa ulizo yaku fwata.**<br><br>**Habari zote mutatu patiya zita tumikishiwa kwa sababu ya utafiti, na akuna mutu (ata binti wenu, ata jamaa yenu wala mutu mwengine wa kijiji/eneo yenu) hata juwa mulitu ambia nini. Yote mutatu ambia ita wekwa kwa siri.**<br><br>**Mbele ya yote, nataka itikisha kama muko vizuri, bila wasiwasi, nakama mume sikia yote juya mada tuta zungumuzia. Eti, muna ulizo mbalimbali? Eti, muna kubali kutu ongelesha leo?**<br><br>**Vizuri sana. Tuna anza na ulizo za tegetege ju yenu. Wakati tuta sema « binti wako » , manayake tunataka sema binti wako anaye kua kwenye ile programme, mwenye uko muzazi wala musimamizi wake.**		
B1	Myaka yako ni ngapi ?	_____________<br>888= Hajuwi<br>999 = Akuna jibu	Kama chini ya myaka 18, acha.

**Caregiver Survey (DRC)**			
**Question #**	**Question**	**Réponse options**	**Instructions**
B4	Ni mtu gani ambaye anasaidiya zaidi jamaa ya [BINTI] kipesa?	1= mama<br>2= baba<br>3= tate<br>4= baba mukwe / mama mukwe<br>5= kaka ao dada<br>6= shangazi/muyomba<br>7= mwanamemba mwengeni wa jamaa<br>8= msimamizi asiye wa jamaa<br>9= mwengine (umutambulishe)<br>888= Hajuwi<br>999 = Akuna jibu	Shagua jibu zote zakweli<br><br>Kama ''musimamizi mwenye haiko mwanamemba wa jamaa wala mutu mwengine'', usikose ku julisha
**C. Les normes des rôles de genre**	**Nataka sasa kukuuliza swali (ulizo) fulani fulani kuhusu na maelekeyo yako kuhusu wabinti na wavulana. Hakuna jibu nzuri ao mbaya. Jibu zako hazita hukumiwa.Ningependa unielezeye kama « ndiyo » ao « apana » unakubali misemwa.**		
C1	Ingekuwa ya muhimu wanaume wawe na urahisi kwa mafunzo kuliko mabinti [wasichana]	1= Ndio<br>2= Apana<br>888= Hajuwi<br>999 = Akuna jibu	
C2	Mabinti walipashwa enda shuleni kama hawana kazi nyumbani	1= Ndio<br>2= Apana<br>888= Hajuwi<br>999 = Akuna jibu	
C3	Sababu ya muhimu ambayo inatuma wavulana wanaenda shuleni kupita wabinti, ni sababu wana weza saidia vizuri wazazi wao wakisha zeheka.	1= Ndio<br>2= Apana<br>888= Hajuwi<br>999 = Akuna jibu	
C4	Kungekuwa pesa ndogo kwa matumizi ya masomo, pesa hizo zingetumikishwa kwanza kwa masomo ya wanaume	1= Ndio<br>2= Apana<br>888= Hajuwi<br>999 = Akuna jibu	
C5	Wanawake walipashwa achiya wanaume kufanya siyasa	1= Ndio<br>2= Apana<br>888= Hajuwi<br>999 = Akuna jibu	
C6	Mwanamke alipashwa kuwa na bwana ao mtoto wa kiume ao msimamizi mwengine wa kiume ambaye anaweza mulindiya usalama sababu hawezi mwenyewe kujikinga	1= Ndio<br>2= Apana<br>888= Hajuwi<br>999 = Akuna jibu	
C7	Kitu cha muhimu ambacho mwanamke anategemea ndani ya uzee wake ni watoto wake wa kiume	1= Ndio<br>2= Apana<br>888= Hajuwi<br>999 = Akuna jibu	
C8	Mwanamke mwema ni yule ambaye hana mashaka kwa wazo ya bwana yake hata angekuwa hakubali kwa kweli	1= Ndio<br>2= Apana<br>888= Hajuwi<br>999 = Akuna jibu	

**Caregiver Survey (DRC)**			
**Question #**	**Question**	**Réponse options**	**Instructions**
C9	Mabinti wangepata nao bahati ya kutumika inje ya jamaa sawa sawa na wavulana.	1= Ndio<br>2= Apana<br>888= Hajuwi<br>999 = Akuna jibu	
C10	Ningependa binti yangu apate kazi inje ya jamaa kusudi ajishurulikiye na ikiwazekana asaidiye jamaa	1= Ndio<br>2= Apana<br>888= Hajuwi<br>999 = Akuna jibu	
**D. Éducation de l'enfant**			
D1	Unawaza kama kwa kuadibisha wabinti, inafaa kuwaazibu kwa kuwapiga fimbo, kofi, …?	1= Ndio<br>2= Apana<br>888= Hajuwi<br>999 = Akuna jibu	
D2	Ku ngazi gani wazo hili ni la kweli ? Ni muhimu kujuwa mahali [BINTI] anapatikana kwa kila wakati	3 = Kweli sana<br>2 = Kweli kidogo<br>1 = Siyo kweli sana<br>888= Hajuwi<br>999 = Akuna jibu	
	**Sasa, ninataka kukuuliza ulizo fulani juu ya maisha ya kesho ungependa [BINTI] akuwe nayo**. **Nitakuuliza swali kuelekeya namna ya kuadibisha watoto. Ningependa nijuwe maoni yako ya kweli. Ukumbuke ya kwamba hakuna jibu nzuri ao mbaya.**		
D3	Binti wako ana andikishwa kwenye masomo kwa saa izi ?	1= Ndio<br>2= Apana<br>888= Hajuwi<br>999 = Akuna jibu	Kama ''Apana'' wala ''akuna jibu'', pita ku ulizo D6
D4	Unaweza penda [BINTI] afike ndani ya somo gani mbele ya kuacha kusoma ?<br><br>Ruka iyi ulizo kama binti haandikishwe ku masomo.	___________(Ngazi ya masomo)<br><br>888= Hajuwi<br>999 = Akuna jibu	
D5	Unaweza penda binti afikiliye miaka ngapi kwa kuendeleya kubakiya ku masomo ?<br><br>Ruka iyi ulizo kama binti haandikishwe ku masomo.	(miaka) ____________	Andika 88 kama ''sijuwi''<br>Andika 99 kama ''Akuna jibu''
D6	Unatumainia kama [BINTI] wakoanaweza olewa ku miaka ngapi ?	(miaka) ___________	Andika 88 kama ''sijuwi''<br>Andika 99 kama ''Akuna jibu''
D7	Unatumainia [BINTI] wakoanaweza zala mtoto wa kwanza ku miaka ngapi ?	(miaka) ___________	Andika 88 kama ''sijuwi''<br>Andika 99 kama ''Akuna jibu''

Caregiver Survey (DRC)			
**Question #**	**Question**	**Réponse options**	**Instructions**
D8	Kama binti alikuelezea ginsi alikuwa muhanga wa ubakaji, unaweza jisikia huru kwa kumuelezea kuhusu huduma zinayo kuwa tayari ? Kama ndiyo, huduma gani ? Kama apana, sababu gani ?	1= Ndio<br>2= Apana<br>888= Hajuwi<br>999 = Akuna jibu	Kama ''apana'' wala ''akuna jibu'', pita ku ulizo D8b
D8a	Kama ndiyo, ni gani ?	1= Huduma za afya kwa munganga<br>2= huduma za kisheriya<br>3= huduma za kimafikiri<br>4= huduma za kijamii na uchumi<br>5= huduma za kijamii na uchumi<br>6= huduma ingine ya kujulisha.<br>888= Hajuwi<br>999= Akuna jibu	Pita ku E1
D8b	Kama apana, sababu gani	I. Sijuwi huduma zilizo tayari<br>II. Huduma hazipatikani<br>III. Sijali, siyo jukumu langu<br>IV. Huduma si nzuri<br>V. Huduma, ni nguvu kuzifikilia<br>(kipesa, mbali)<br>VI. Kitu kingine (tambulisha).<br>888= Hajuwi<br>999= Akuna Jibu	
**E. Comportement parental**	**Hizi ni maelezo kuhusu desturi mbalimbali ambazo wazazi na mawasimamizi wanatumia wakati moja moja kwa watoto wao. Samahani, chaguwa, kwa maoni yako kama maelezo iko « karibu kweli siku zote », « mara na mara kweli », « mara haba kweli » ao « karibu kamwe kweli »**		
E1	Ninasema vitu vipendevu kuhusu mtoto wangu	[0] karibu siku zote kweli<br>[1] mara na mara kweli<br>[2] mara haba kweli<br>[3] karibu kamwe kweli<br>888= Hajuwi<br>999= Akuna	

**Caregiver Survey (DRC)**			
**Question #**	**Question**	**Réponse options**	**Instructions**
E2	Sijiusishe sana na mtoto wangu	[0] karibu siku zote kweli<br><br>[1] mara na mara kweli<br><br>[2] mara haba kweli<br><br>[3] karibu kamwe kweli<br>888= Hajuwi<br>999= Akuna jibu	
E3	Nina raisisha mtoto wangu kwa kunizungumuziya mambo kwa urahisi	[0] karibu siku zote kweli<br><br>[1] mara na mara kweli<br><br>[2] mara haba kweli<br><br>[3] karibu kamwe kweli<br><br>888= Hajuwi<br>999= Akuna jibu	
E4	Nina azibu mtoto wangu hata kama haistahili.	[0] karibu siku zote kweli<br><br>[1] mara na mara kweli<br><br>[2] mara haba kweli<br><br>[3] karibu kamwe kweli<br>888= Hajuwi<br>999= Akuna jibu	
E5	Ninaona mtoto wangu kama ni wakunizuru	[0] ni kweli karibu siku zote<br><br>[1] mara na mara ni kweli<br><br>[2] ni kweli mara haba<br><br>[3] ni kweli karibu kamwe<br><br>888= Hajuwi<br><br>999= Akuna jibu	
E6	Nikisirika, nina azibu mtoto wangu	[0] ni kweli karibu siku zote<br><br>[1] mara na mara ni kweli<br><br>[2] ni kweli mara haba<br><br>[3] ni kweli karibu kamwe<br><br>888= Hajuwi<br><br>999= Akuna jibu	

**Caregiver Survey (DRC)**			
**Question #**	**Question**	**Réponse options**	**Instructions**
E7	Nina fungwa na kazi nyingi pahali ya kujibu ku maulizo za mtoto wangu	[0] karibu siku zote kweli<br>[1] mara na mara kweli<br>[2] mara haba kweli<br>[3] karibu kamwe kweli<br>888= Hajuwi<br>999= Akuna jibu	
E8	Ninasikiya kuchukiya mtoto wangu	[0] karibu siku zote kweli<br>[1] mara na mara kweli<br>[2] mara haba kweli<br>[3] karibu kamwe kweli<br>888= Hajuwi<br>999= Akuna jibu	
E9	Nina shurulika na yote mtoto wangu anafanya	[0] karibu siku zote kweli<br>[1] mara na mara kweli<br>[2] mara haba kweli<br>[3] karibu kamwe kweli<br>888= Hajuwi<br>999= Akuna jibu	
E10	Ninasema vitu vingi visivyo vizuri mbele ya mtoto wangu	[0] karibu siku zote kweli<br>[1] mara na mara kweli<br>[2] mara haba kweli<br>[3] karibu kamwe kweli<br>888= Hajuwi<br>999= Akuna jibu	

**Caregiver Survey (DRC)**			
**Question #**	**Question**	**Réponse options**	**Instructions**
E11	Sijibu kwa ombi ya mtoto wangu	[0] karibu siku zote kweli<br>[1] mara na mara kweli<br>[2] mara haba kweli<br>[3] karibu kamwe kweli<br>888= Hajuwi<br>999= Akuna jibu	
E12	Ninahakika ya kwamba mtoto wangu anajua ya kwamba ni mwenyi mafaa na kuhitajika	[0] karibu siku zote kweli<br>[1] mara na mara kweli<br>[2] mara haba kweli<br>[3] karibu kamwe kweli<br>888= Hajuwi<br>999= Akuna jibu	
E13	Nina mchunguza sana kwamakini mtoto wangu	[0] karibu siku zote kweli<br>[1] mara na mara kweli<br>[2] mara haba kweli<br>[3] karibu kamwe kweli<br>888= Hajuwi<br>999= Akuna jibu	
E14	Ninamutendeya mototo wangu vibaya kwa kimafikiri	[0] karibu siku zote kweli<br>[1] mara na mara kweli<br>[2] mara haba kweli<br>[3] karibu kamwe kweli<br>888= Hajuwi<br>999= Akuna jibu	

Caregiver Survey (DRC)			
**Question #**	**Question**	**Réponse options**	**Instructions**
E15	Ninafanya ginsimtoto wangu ajisikiye ametupiliwa wakati anafanya kosa.	[0] karibu siku zote kweli<br>[1] mara na mara kweli<br>[2] mara haba kweli<br>[3] karibu kamwe kweli<br>888= Hajuwi<br>999= Akuna jibu	
E16	Ninasahabu vitu vya lazima vyenyi mtoto wangu anajua kama ni vya lazima kuvikumbuka.	[0] karibu siku zote kweli<br>[1] mara na mara kweli<br>[2] mara haba kweli<br>[3] karibu kamwe kweli<br>888= Hajuwi<br>999= Akuna jibu	
E17	Nafanya yote kusudi mtoto wangu ajisikiye ya kwamba kitu anayo fanya ni ya lazima sana.	0] karibu siku zote kweli<br>[1] mara na mara kweli<br>[2] mara haba kweli<br>[3] karibu kamwe kweli<br>888= Hajuwi<br>999= Akuna jibu	
E18	Mtoto wangu akifanya kosa fulani, nina muogopesha ao kumutisha.	[0] karibu siku zote kweli<br>[1] mara na mara kweli<br>[2] mara haba kweli<br>[3] karibu kamwe kweli<br>888= Hajuwi<br>999= Akuna jibu	

**Caregiver Survey (DRC)**			
**Question #**	**Question**	**Réponse options**	**Instructions**
E19	Nina kubali yote mtoto wangu anawaza na nina mutiya moyo kuyasema.	[0] karibu siku zote kweli<br><br>[1] mara na mara kweli<br><br>[2] mara haba kweli<br><br>[3] karibu kamwe kweli<br><br>888= Hajuwi<br>999= Akuna jibu	
E20	Nina waza ya kwamba watoto wengine ni wenyi kufaa kupita wangu.	[0] karibu siku zote kweli<br><br>[1] mara na mara kweli<br><br>[2] mara haba kweli<br><br>[3] karibu kamwe kweli<br><br>888= Hajuwi<br>999= Akuna jibu	
E21	Ninajulisha mtoto wangu ya kwamba simutaki.	[0] karibu siku zote kweli<br><br>[1] mara na mara kweli<br><br>[2] mara haba kweli<br><br>[3] karibu kamwe kweli<br><br>888= Hajuwi<br>999= Akuna jibu	
E22	Ninajulisha mtoto wangu ya kwamba nina mupenda	[0] karibu siku zote kweli<br><br>[1] mara na mara kweli<br><br>[2] mara haba kweli<br><br>[3] karibu kamwe kweli<br><br>888= Hajuwi<br>999= Akuna jibu	
E23	Sishurulikiye mtoto wangu kabla hajanisumbuwa.	[0] karibu siku zote kweli<br><br>[1] mara na mara kweli<br><br>[2] mara haba kweli<br><br>[3] karibu kamwe kweli<br><br>888= Hajuwi<br>999= Akuna jibu	

**Caregiver Survey (DRC)**			
**Question #**	**Question**	**Réponse options**	**Instructions**
E24	Ninashurulikiya mototo wangu kwa uzuri ili nimupendeze.	[0] karibu siku zote kweli<br><br>[1] mara na mara kweli<br><br>[2] mara haba kweli<br><br>[3] karibu kamwe kweli<br><br>888= Hajuwi<br>999= Akuna jibu	
**F. Mwenendo juu ya adibisho**	**Mara na mara, wakati wazazi ao mtu ambaye anahusika na watoto anasirika ao anafazaika kufwatana na vitendo fulani vya watoto, anawapiga. Kama ingekuwa wewe, wakati gani wazazi wanakuwa na haki ya kupiga watoto ?**		
F1	Wanaweza wapiga … kama mtoto si mtiifu	1= Ndio<br>2= Apana<br>888= Hajuwi<br>999 = Akuna jibu	
F2	Wanaweza wapiga … kama mtoto hakubaliane na mzazi	1= Ndio<br>2= Apana<br>888= Hajuwi<br>999 = Akuna jibu	
F3	Wanaweza wapiga … kama mtoto anakimbiya ku nyumba	1= Ndio<br>2= Apana<br>888= Hajuwi<br>999 = Akuna jibu	
F4	Wanaweza wapiga … kama mtoto hapendi enda shuleni	1= Ndio<br>2= Apana<br>888= Hajuwi<br>999 = Akuna jibu	
F5	Wanaweza wapiga … ama mtoto hapendi fanya kazi	1= Ndio<br>2= Apana<br>888= Hajuwi<br>999 = Akuna jibu	
F6	Wanaweza wapiga … ama mtoto hashurulikiye wakaka na wadada wake	1= Ndio<br>2= Apana<br>888= Hajuwi<br>999 = Akuna jibu	
F7	Wanaweza wapiga … ama mtoto anafanya ukahaba kwa kusukumwa na mtu mzima	1= Ndio<br>2= Apana<br>888= Hajuwi<br>999 = Akuna jibu	
F8	Wanaweza wapiga … ama mtoto anakojowa kitandani	1= Ndio<br>2= Apana<br>888= Hajuwi<br>999 = Akuna jibu	
F9	Wanaweza wapiga … ama mtoto ni mwizi	1= Ndio<br>2= Apana<br>888= Hajuwi<br>999 = Akuna jibu	

Caregiver Survey (DRC)			
**Question #**	**Question**	**Réponse options**	**Instructions**
F10	Wanaweza wapiga … ama mtoto ni mlevi ao mwenyi kutumiya vitu vya kulevya.	1= Ndio<br>2= Apana<br>888= Hajuwi<br>999 = Akuna jibu	
F11	Wanaweza wapiga … ama mtoto anakataa kuolewa.	1= Ndio<br>2= Apana<br>888= Hajuwi<br>999 = Akuna jibu	
**J. Decision-making and gender norms**			
**J1**	Nani ambaye anakamata maamuzi za mwisho kwa utumiyaji ya pesa ambayo unapataka ?	1 = Zaidi wewe / Wewe pekee<br>2 = Zaidi mume wako / mushiriki<br>3 = Mume wako / mushiriki na wewe sawa sawa<br>4 = Sana sana mtu mwengine wa mu jamaa<br>5 = Haiambatane kama jamaa haiusiki na hiyo mada	
**J2**	Nani ambaye anakamata maamuzi za mwisho kuhusu utumiyaji ya pesa yenye bwana yako anapataka ?	1 = Zaidi wewe / Wewe pekee<br>2 = Zaidi mume wako / mushiriki<br>3 = Mume wako / mushiriki na wewe sawa sawa<br>4 = Sana sana mtu mwengine wa mu jamaa<br>5 = Haiambatane kama jamaa haiusiki na hiyo mada	
**J3**	Nani ambaye anakamata maamuzi za mwisho kuhusu matunzo yako ?	1 = Zaidi wewe / Wewe pekee<br>2 = Zaidi mume wako / mushiriki<br>3 = Mume wako / mushiriki na wewe sawa sawa<br>4 = Sana sana mtu mwengine wa mu jamaa<br>5 = Haiambatane kama jamaa haiusiki na hiyo mada	
**J4**	Nani ambaye anakamata maamuzi za mwisho kuhusu vitu vya lazima vya kununua ndani ya nyumba?	1 = Zaidi wewe / Wewe pekee<br>2 = Zaidi mume wako / mushiriki<br>3 = Mume wako / mushiriki na wewe sawa sawa<br>4 = Sana sana mtu mwengine wa mu jamaa<br>5 = Haiambatane kama jamaa haiusiki na hiyo mada	

Caregiver Survey (DRC)			
**Question #**	**Question**	**Réponse options**	**Instructions**
**J5**	Nani ambaye anakamata maamuzi za mwisho kuhusu utumiyaji kidogo wa pesa (kutoka ya kawaida) kwa ajili ya jamaa?	1 = Zaidi wewe / Wewe pekee<br>2 = Zaidi mume wako / mushiriki<br>3 = Mume wako / mushiriki na wewe sawa sawa<br>4 = Sana sana mtu mwengine wa mu jamaa<br>5 = Haiambatane kama jamaa haiusiki na hiyo mada	
**J6**	Nani ambaye anakamata maamuzi za mwisho kuhusu kutembeleya jamaa ao wazazi wako?	1 = Zaidi wewe / Wewe pekee<br>2 = Zaidi mume wako / mushiriki<br>3 = Mume wako / mushiriki na wewe sawa sawa<br>4 = Sana sana mtu mwengine wa mu jamaa<br>5 = Haiambatane kama jamaa haiusiki na hiyo mada	
**J7**	Nani ambaye anakamata maamuzi za mwisho kuhusu utumiyaji wa pesa kwa ajili ya masomo ao kusomesha watoto?	1 = Zaidi wewe / Wewe pekee<br>2 = Zaidi mume wako / mushiriki<br>3 = Mume wako / mushiriki na wewe sawa sawa<br>4 = Sana sana mtu mwengine wa mu jamaa<br>5 = Haiambatane kama jamaa haiusiki na hiyo mada	
**J8**	Kwa mawazo yako, unawaza kama inafasiriwa mume apige bibi yake kama akitoka bila ku muambiya ?	1 = Ndiyo<br>2 = Hapana	
**J9**	kwa mawazo yako, inafasiriwa mume apige bibi yake kama hashurulikiye watoto ?	1 = Ndiyo<br>2 = Hapana	
**J10**	kwa mawazo yako, inafasiriwa mume apige bibi yake kama anabishana naye ?	1 = Ndiyo<br>2 = Hapana	
**J11**	Kwa mawazo yako, inafasiriwa mume apige bibi yake akikatala kufanya kitendo cha ndowa naye ?	1 = Ndiyo<br>2 = Hapana	
**J12**	Kwa mawazo yako, inafasiriwa mume apige bibi yake akilunguza cakula ?	1 = Ndiyo<br>2 = Hapana	
**J13**	Kwa mawazo yako, inafasiriwa mume apige bibi yake kama akikatala kumutii ?	1 = Ndiyo<br>2 = Hapana	

Caregiver Survey (DRC)			
**Question #**	**Question**	**Réponse options**	**Instructions**
**J14**	Kwa mawazo yako, inafasiriwa mume apige bibi yake akijuwa ya kwamba hakukuwa mwaminifu ?	1 = Ndiyo<br>2 = Hapana	
**J15**	Kwa mawazo yako, inafasiriwa mwanamke akatale kitendo cha ndoa na bwana yake wakati anajuwa ya kwamba bwana yake anaugonjwa malali ya ngono ?	1 = Ndiyo<br>2 = Hapana	
**J16**	Kwa mawazo yako, inafasiriwa mwanamke akatale kitendo cha ndoa na bwana yake wakati anajuwa ya kwamba bwana yake anakuwa na vitendo vya ndoa na mwengine mwanamuke ?	1 = Ndiyo<br>2 = Hapana	
**J17**	Kwa mawazo yako, inafasiriwa mwanamke akatale kitendo cha ndoa na bwana yake wakati anatoka kuzala?	1 = Ndiyo<br>2 = Hapana	
**J18**	Kwa mawazo yako, inafasiriwa mwanamke akatale kitendo cha ndoa na bwana yake wakati anachoka ao hajisikiye vizuri ?	1 = Ndiyo<br>2 = Hapana	
**J19**	Kwa mawazo yako, inafasiriwa mwanamke akatale kitendo cha ndoa na bwana yake, wakati huyu analewa ?	1 = Ndiyo<br>2 = Hapana	
**J20**	Kwa mawazo yako, inafasiriwa mwanamke akatale kitendo cha ndoa na bwana yake wakati huyu anamutesa ?	1 = Ndiyo<br>2 = Hapana	
**J21**	Kwa mawazo yako, inafasiriwa mwanamke akatale kitendo cha ndoa na bwana yake wakati huyu anakataa utumiyaji ya kapoti ?	1 = Ndiyo<br>2 = Hapana	
**G. Questions de Conclusion**	**Aksanti kuona uli jibu ulizo zote. Nina hakika ulizo zimoya zilikua ziumu. Uku kumbuke kama akuna mutu wa kijiji/eneo lako hata jua kamwe jibu zenye ulitu elezeya.**<br>**Aksanti tena kwaku jibu, uli fanya kazi muzuri sana.**<br>**Tumeisha maliza, alakini mbele yaku komesha mazungumuzo yetu, nina ulizo zingine tena kwa ajili yako.**		
G1	Ulizo zilizo pita, eti zilikua namuna gani ?	1= Rahisi sana ku sikia<br>2= Rahisi kidogo ku sikia<br>3= Ngumu ku sikia<br>4=Ngumu zaidi ku sikia<br>888= Hajuwe<br>999= Akuna jibu	
G2	Kwa ujumla, kwa ngazi gani uli sema ukweli kwa ulizo zote ?	1= Akuna ukweli kamwe<br>2= Akuna kabisa ukweli<br>3= Kwa ukweli kidogo<br>4= Kwa ukweli kabisa<br>5= kwa ukweli sana<br>888= Hajuwi<br>999= Akuna jibu	

Caregiver Survey (DRC)			
**Question #**	**Question**	**Réponse options**	**Instructions**
G3	Kuna jambo fulani unaweza kuongeza wala ulizo uakuni uliza ?		
G4	Una wazo fulani wala mawazo fulani juya iyi kiulizo ?		
	**(LE/LA REMERCIER POUR SA DISPONIBILITÉ. LUI ASSURER QUE SES RÉPONSES SONT CONFIDENTIELLES. L'INFORMER QU'IL/ELLE PEUT CONTACTER L'ÉQUIPE DE RECHERCHE N'IMPORTE QUAND POUR DES QUESTIONS ET DES PRÉOCCUPATIONS.)**<br><br>**Faite la transition a l'enquête des filles si c'est possible**		
**H. Post Interview Enumérateur-only Questions**	**ENQUÊTEUR: MERCI DE RÉPONDRE AUX QUESTIONS SUIVANTES AVANT DE TERMINER L'INTERVIEW**		
H1	La personne semblait-elle comprendre les questions?	1= Tout le temps<br>2<br>3= De temps en temps<br>4<br>5= Jamais	Choisir entre 1 et 5
H2	La personne semblait-elle répondre aux questions de façon aléatoire?	1= Tout le temps<br>2<br>3= De temps en temps<br>4<br>5= Jamais	Choisir entre 1 et 5
H3	La personne semblait-elle réfléchir aux réponses avant de répondre ?	1= Tout le temps<br>2<br>3= De temps en temps<br>4<br>5= Jamais	Choisir entre 1 et 5
H4	Y'avait-il quelqu'un d'autre présent au moment de l'interview?	1=Oui<br>2=Non	Si "Oui", procéder à H4b and H4c<br>Si "Non", passer à H5
H4b	Qui était cette autre personne?	_________	Écrire seulement la relation
H4c	Jusqu'à quel niveau sentez-vous que la présence de cette personne influençait les réponses données par la personne?	1= Beaucoup<br>2= Un peu<br>3= Très peu<br>4= Pas du tout	
H5	L'interview a-t-elle été interrompue pour une quelconque raison?	1=Oui<br>2=Non	Si "Oui", procéder à H5b and H5c<br>Si "Non", passer à H6
H5b	Pourquoi l'interview a-t-elle été interrompue?	_______________	
H5c	Selon vous, cela a-t-il affecté l'interview?	1=Oui<br>2=Non	
H6	Quel était le contexte dans lequel l'interview a eu lieu?	1= Calme, privé<br>2= Quelque bruit, presque privé<br>3= Trop de bruit, des gens tout autour	

Caregiver Survey (DRC)			
**Question #**	**Question**	**Réponse options**	**Instructions**
H7	Comment évalueriez-vous la capacité de la personne de comprendre la plupart des questions?	1= N'a pas beaucoup compris<br>2= A compris un peu<br>3= A compris modérément<br>4= A beaucoup compris<br>5= A compris énormément	
H8	Quelles questions ont semblé plus difficiles à comprendre pour la personne?		S'il vous plait, lister les questions
H9	En général, comment évaluerez-vous l'intérêt que la personne portait à l'interview?	1= Vraiment haut<br>2= Au dessus de la moyenne<br>3= Moyenne<br>4= En dessous de la moyenne<br>5= Vraiment bas	
H10	La personne a-t-elle besoin d'une référence à un prestataire de services quelconque?	____________________<br>____________________<br>____________________<br>____________________<br>____________________	Si oui, décrire s'il vous plait.
	**FIN DU QUESTIONNAIRE, TRÈS BON TRAVAIL!**		
